# The Anti-sigma Factor RsiV Is a Bacterial Receptor for Lysozyme: Co-crystal Structure Determination and Demonstration That Binding of Lysozyme to RsiV Is Required for σ^V^ Activation

**DOI:** 10.1371/journal.pgen.1006287

**Published:** 2016-09-07

**Authors:** Jessica L. Hastie, Kyle B. Williams, Lindsey L. Bohr, Jon C. Houtman, Lokesh Gakhar, Craig D. Ellermeier

**Affiliations:** 1 Department of Microbiology, Carver College of Medicine, University of Iowa, Iowa City, Iowa, United States of America; 2 Department of Biochemistry & Protein Crystallography Facility, Carver College of Medicine, University of Iowa, Iowa City, Iowa, United States of America; A*STAR, SINGAPORE

## Abstract

σ factors provide RNA polymerase with promoter specificity in bacteria. Some σ factors require activation in order to interact with RNA polymerase and transcribe target genes. The Extra-Cytoplasmic Function (ECF) σ factor, σ^V^, is encoded by several Gram-positive bacteria and is specifically activated by lysozyme. This activation requires the proteolytic destruction of the anti-σ factor RsiV via a process of regulated intramembrane proteolysis (RIP). In many cases proteases that cleave at site-1 are thought to directly sense a signal and initiate the RIP process. We previously suggested binding of lysozyme to RsiV initiated the proteolytic destruction of RsiV and activation of σ^V^. Here we determined the X-ray crystal structure of the RsiV-lysozyme complex at 2.3 Å which revealed that RsiV and lysozyme make extensive contacts. We constructed RsiV mutants with altered abilities to bind lysozyme. We find that mutants that are unable to bind lysozyme block site-1 cleavage of RsiV and σ^V^ activation in response to lysozyme. Taken together these data demonstrate that RsiV is a receptor for lysozyme and binding of RsiV to lysozyme is required for σ^V^ activation. In addition, the co-structure revealed that RsiV binds to the lysozyme active site pocket. We provide evidence that in addition to acting as a sensor for the presence of lysozyme, RsiV also inhibits lysozyme activity. Thus we have demonstrated that RsiV is a protein with multiple functions. RsiV inhibits σ^V^ activity in the absence of lysozyme, RsiV binds lysozyme triggering σ^V^ activation and RsiV inhibits the enzymatic activity of lysozyme.

## Author Summary

The exposed cell wall of Gram-positive bacteria renders them particularly susceptible to the innate immune defense enzyme lysozyme. Several Gram-positive bacteria activate lysozyme resistance via a signal transduction system, σ^V^, which is induced by lysozyme. Here we report the co-structure of lysozyme with its bacterial receptor the anti-σ factor RsiV. In the absence of lysozyme, RsiV inhibits activity of σ^V^. In the presence of lysozyme, RsiV is destroyed via proteolytic cascade. We demonstrate that binding of lysozyme to RsiV triggers the proteolytic destruction of the anti-σ factor RsiV and thus activation of σ^V^. In addition, we demonstrate that RsiV also acts as an inhibitor of lysozyme activity. Thus, the anti-σ factor RsiV allows for the cell to sense lysozyme and inhibit its activity as well as inducing additional lysozyme resistance mechanisms.

## Introduction

In order to survive in rapidly changing environmental conditions, bacteria use signal transduction systems to transmit information from outside the cell across the membrane to alter transcriptional responses. In bacteria, Extra-Cytoplasmic Function (ECF) σ factors are one class of signal transduction system capable of responding to extracellular signals. ECF σ factors represent the largest and most diverse group of σ factors [1]. However, one common feature of many ECF σ factors is that they are sequestered in an inactive state by an anti-σ factor and must be activated in order to interact with RNA polymerase. In many cases the signals that induce activity of these ECF σ factors and the molecular mechanisms controlling activation are not well understood. The anti-σ factor is responsible for inhibiting ECF σ factor activity by blocking its association with RNA polymerase in the absence of signal. Activation of ECF σ factors occurs via modification of the anti-σ factor, leading to release of the ECF σ factor or transition the ECF σ factor to an active state, allowing interaction with RNA polymerase.

The activation of several ECF σ factors occurs via a mechanism termed Regulated Intramembrane Proteolysis (RIP), which results in the sequential cleavage of the anti-σ factor in response to extracellular stress [2–4]. RIP is initiated by a cleavage event at site-1 of the anti-σ factor and this initial cleavage event usually occurs on an extracellular domain of the anti-σ factor. Following site-1 cleavage a second protease cuts within the transmembrane domain of the anti-σ factor at site-2. The remaining cytosolic portion of the anti-σ factor is then destroyed by cytosolic proteases [2–4].

The *B*. *subtilis* ECF σ factor, σ^V^, belongs to the ECF30 subfamily of ECF σ factors, which are found almost exclusively in Firmicutes (low GC Gram-positive bacteria) [1]. The activity of a subset of the ECF30 σ factor homologs are inhibited by anti-σ factors homologous to RsiV. C-type lysozyme activates σ^V^ in *B*. *subtilis* [5,6] and in other bacteria encoding homologous systems including *C*. *difficile* and *E*. *faecalis* [7–10]. σ^V^ is activated by RIP mediated degradation of the transmembrane anti-σ factor RsiV in response to C-type lysozyme [11,12]. In each of these organisms free σ^V^ then interacts with RNA polymerase to transcribe genes required for lysozyme resistance [5,6,8,9,13,14]. In *B*. *subtilis* this includes *oatA* which encodes a peptidoglycan O-acetylase that adds an acetyl group to peptidoglycan [15] which increases resistance to lysozyme [5,6]. σ^V^ is also required for lysozyme inducible expression of *dltABCDE* which encode enzymes responsible for the addition of D-alanine (D-ala) to teichoic acids of the cell wall [6,16]. Increasing the positive charge of the teichoic acids presumably repels the positively charged lysozyme [14,17–22]. Activation of σ^V^ in *C*. *difficile* results in a large increase in expression of the operon encoding σ^V^ and RsiV (*pdaVprsA2csfVrsiVcd1560*) [8] and to a lesser degree the *dltABCDE* operon [14].

The activity of σ^V^ is inhibited by RsiV. In order to activate σ^V^ RsiV must be degraded in a RIP-dependent manner. Previous work from our laboratory has shown signal peptidase is responsible for the initial site-1 cleavage of RsiV, which removes the extracellular domain of RsiV [12]. Following site-1 cleavage, the truncated form of RsiV is cleaved by the site-2 protease RasP [11]. In other ECF σ factor systems RIP is initiated by the site-1 protease which is thought to be responsible for sensing the inducing signal and initiating RIP of the anti-σ [2,3]. In the case of *E*. *coli*, σ^E^ activation is initiated by the binding of unfolded outer membrane proteins to the site-1 protease DegS [23,24]. Interestingly RseB a negative regulator of site-1 cleavage has also been implicated in sensing envelope stress by directly binding lipid A fragments [25]. In the case of *B*. *subtilis* σ^W^ activation, it is less clear how PrsW controls site-1 cleavage in response to cell stress, but evidence suggests that it can act as a sensor since constitutively active mutants have been isolated that alter PrsW activity [26].

Since the activity of signal peptidase is not known to be controlled by extracellular signals, it raised the question of how site-1 cleavage of RsiV is initiated in the presence of lysozyme [27,28]. Interestingly signal peptidase could cleave RsiV at site-1 *in vitro* but only in the presence of lysozyme [12]. We found the anti-σ factor RsiV directly binds to lysozyme [12]. In addition, we found that lysozyme but not the structurally distinct muramidase mutanolysin could activate and bind RsiV. Thus we proposed a model where activation of σ^V^ is controlled by the binding of lysozyme to the anti-σ factor RsiV.

Here we demonstrate that the binding of RsiV to lysozyme is required for activation of the *B*. *subtilis* ECF σ factor σ^V^ in response to lysozyme. We determined the x-ray co-crystal structure of the RsiV-hen egg white (HEW) lysozyme at 2.3 Å. Using the structure as a guide we identify residues required for the binding of RsiV to lysozyme and demonstrate that this binding is essential to induce proteolysis of RsiV and thus σ^V^ activation. Taken together this demonstrates that RsiV is a receptor for lysozyme and that binding of RsiV to lysozyme is required for initiating RIP and σ^V^ activation in *B*. *subtilis*. The structure also revealed RsiV binds to the active site of lysozyme and we show that RsiV can inhibit activity of lysozyme *in vitro* and RsiV mutants that are unable to bind lysozyme are also unable to inhibit its activity.

## Results

### Co-crystal structure of RsiV and lysozyme

**Fig 1:**
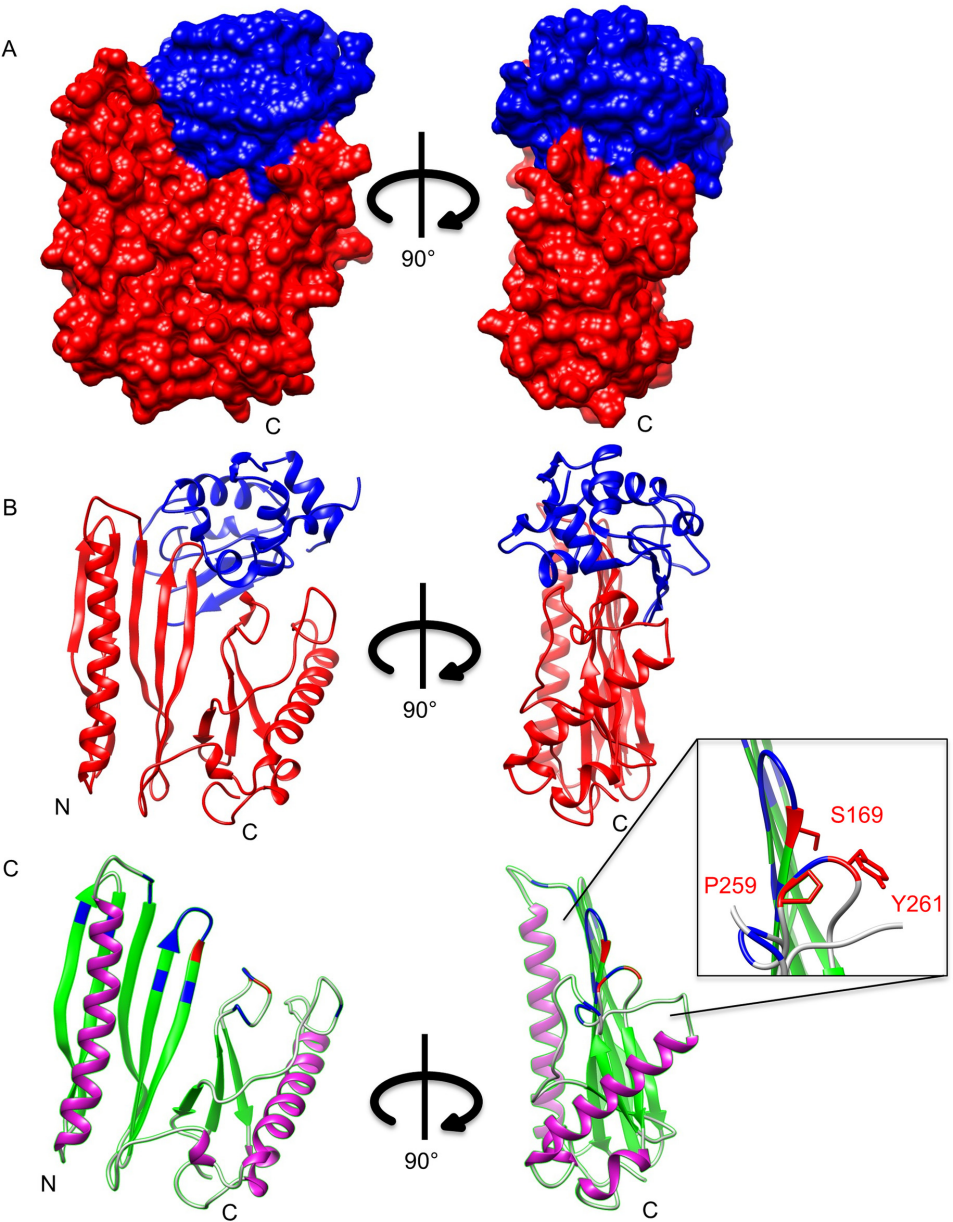
Co-crystal structure of RsiV and lysozyme. Structure of the RsiV-hen egg white (HEW) lysozyme complex at 2.3 Å. **A.** The space fill model of the RsiV-lysozyme complex. The determined structure of RsiV encompasses amino acids 76–285. RsiV is shown in red and lysozyme is shown in blue. The image on the right has been rotated 90° clockwise. **B.** Cartoon diagram of RsiV-lysozyme complex. RsiV is shown in red and lysozyme is shown in blue. On the right RsiV-lysozyme complex cartoon diagram rotated 90° clockwise. **C.** Cartoon diagram of RsiV with lysozyme removed. Image on the right rotated 90° clockwise. The α-helices are shown in purple, β-sheets in green and loops in gold. Amino acids of RsiV that interact with lysozyme are shown in blue. The critical residues required for binding of RsiV to lysozyme are shown in red and magnified in the inset.

We previously found the extracellular domain of RsiV (RsiV^58-285^) was able to bind C-type lysozyme both *in vitro* and *in vivo* [12]. To better understand this interaction we determined the x-ray crystal structure of the RsiV-lysozyme complex at 2.3 Å (PDB ID: 5JEN; Fig 1A and Table 1). The RsiV-lysozyme complex crystallized as a heterodimer and the crystal structure revealed extensive contacts between RsiV and lysozyme (Fig 1A). Based on PDBePISA analysis RsiV has a surface area of 12184 Å^2^ and lysozyme has a surface area of 6503 Å^2^ and they share an interface of 1407.3 Å^2^ [29] further demonstrating the large interaction interface between the two proteins.

**Table 1:** Crystallography statistics.

Data Collection	
Space group	P2_1_2_1_2
Unit cell parameters (Å)	a = 45.21, b = 130.33, c = 136.71
Resolution (Å)	60.53–2.30 (2.38–2.30)
R_merge_	10.9 (89.9)
R_pim_	6.5 (54.9)
Unique Reflections	36905 (3585)
<I/σ(I)>	14.0 (2.6)
Completeness (%)	100.0 (99.9)
Multiplicity	7.2 (7.0)
Anomalous completeness (%)	98.9 (94.0)
Anomalous multiplicity	3.7 (3.4)
**Refinement**	
Resolution (Å)	47.2–2.3
No. reflections	69289
No. reflections (non-anomalous)	36828
R_work_/R_free_	18.1/23.7
No. total atoms	5912
**B factors**	
Wilson (Å^2^)	30.4
Average (Å^2^)	
Chain A	36.0
Chain B	39.3
Chain C	34.9
Chain D	35.8
Waters	33.4
**Rms deviations**	
Bond lengths (Å)	0.014
Bond angles (°)	1.159
**Molprobity statistics**	
Ramachandran favored (%)	96.38
Allowed (%)	3.62
Outliers (%)	0.00
All-atom clashscore	2.98
Solvent content (%)	44.1%

RsiV is made up of two domains of unknown function (DUF4163 and DUF3298) that are often located within the same protein (S1 Fig). The crystal structure revealed that the two DUF domains are tightly associated (S1 Fig). The amino acids (79–162) of RsiV make up DUF4163 and the amino acids (188–270) encompass DUF3298 (S1 Fig) [30]. The N-terminal domain of the RsiV structure which encompasses DUF4163 shows structural similarity to β-grasp domain containing protein Rv1980c from *M*. *tuberculosis* (PDB:2HHI) (S2 Fig) [31–33]. Using Vast+ we determined the best structural match to RsiV in the PDB is 3CYG an uncharacterized protein from *Fervidobacterium nodosum* rt17 (S3 Fig) [34]. There is also similarity to two other proteins of unknown function BF2082 from *Bacteroides fragilis* NCTC 9343 (PDB:3S5T) and PA4972 from *Pseudomonas aeruginosa* PA01 (PDB:4E72) (S2 Fig) [34]. However, none of these proteins are encoded near ECF σ factors and in some cases (PA4972) do not appear to contain a transmembrane domain. In addition the amino acid homology between these structures is very limited (S3 Fig). To determine if lysozyme binding was a feature retained by this entire class of proteins we obtained a clone of 6xHis-Rv1980c used to determine the structure of Rv1980c [31]. We tested the ability of Rv1980c to bind lysozyme using co-purification. We found that when 6xHis-Rv1980c was bound to a nickel column lysozyme did not co-elute with Rv1980c (S4 Fig). This suggests that not all proteins containing DUF4163 and DUFM3298 bind lysozyme.

### Identification of RsiV residues involved in binding lysozyme

**Fig 2:**
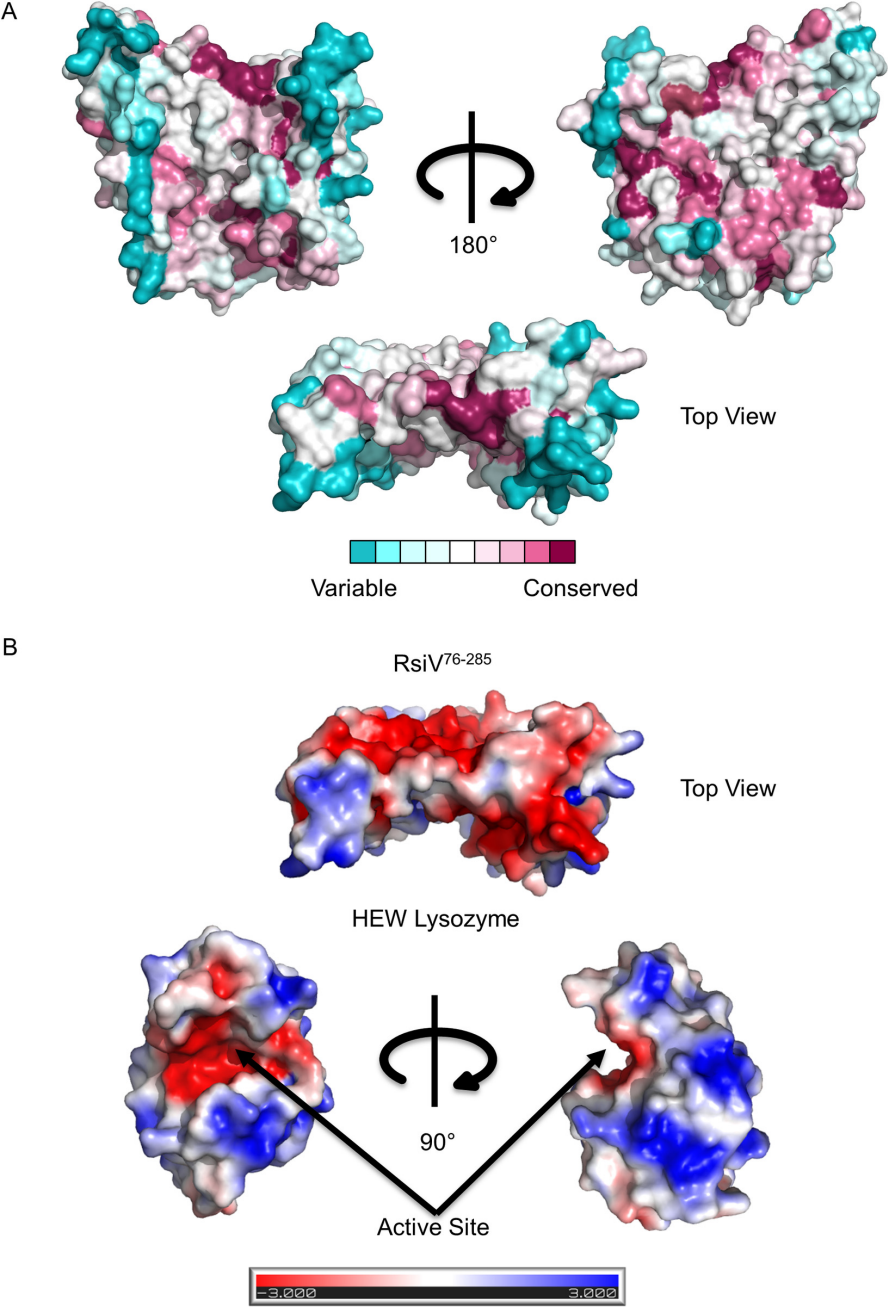
Regions of highest homology mapped on the structure of RsiV. **A.** Space fill model of RsiV with lysozyme removed. The amino acid residues of RsiV are colored according to degree of conservation with 898 other RsiV homologs using ClustalW [36] (S1 Table). The ClustalW homology was overlaid on the RsiV structure using ConSurf [37,38]. The darker maroon color indicates higher conservation while white is neutral and the darker blues are the least conserved amino acid residues. The image on the right has been rotated 180° clockwise. Below is a top view of the lysozyme binding region of RsiV. **B.** Electrostatics and surface potentials were determined through modeling biomolecular solvation with the Adaptive Poisson-Boltzmann Solver (APBS) module with PyMOL. ±3 kT/e electrostatic potential is plotted on the solvent accessible surface of both RsiV76-285 and HEW lysozyme [99]. The top portion is a top down view of RsiV showing the lysozyme binding region. Below is the surface potential of lysozyme showing the active site and the region that interacts with RsiV.

In order to determine the biological significance of RsiV binding lysozyme we sought to disrupt the interaction. To accomplish this, we used the co-crystal structure to identify residues involved in the interaction between RsiV and lysozyme (Table 2). We identified multiple residues that were not grouped together in a single linear segment but were spread over three distinct loops that come together when RsiV is folded (Fig 1C). RsiV homologs are present in a number of different Firmicutes [1] and we demonstrated RsiV homologs from *C*. *difficile* and *E*. *faecalis* bind lysozyme [12]. This suggests that lysozyme binding is likely conserved amongst RsiV homologs. BLAST was used to identify the sequences of 898 RsiV homologs (S1 Table) [35]. A multiple sequence alignment of these homologs was then generated using ClustalW [36]. Using this alignment, we mapped sequence conservation onto the structure of RsiV using ConSurf [37,38]. This analysis revealed a pocket of high conservation in the “saddle” of RsiV of which numerous residues directly contact lysozyme (Fig 2A and Table 2). The most highly conserved residues aligned with those that interact with lysozyme, as determined by our co-crystal structure. We performed similar analysis on 400 C-type lysozymes. This analysis revealed that the highest conservation is located in the peptidoglycan binding pocket and active site of lysozyme which is where RsiV binds to lysozyme (S5 Fig). Thus as expected the most highly conserved regions of both RsiV homologs and lysozyme are involved in the interaction between the two proteins.

**Table 2:** Residues that interact with lysozyme^a^^b^.

RsiV	Lysozyme	Distance A°	Role in Lysozyme
H139	D101	2.46	
S168	Q57	2.66	
Y255	E35	2.7	Active Site
G260	F34	2.8	
Q166	W63	2.81	PG Binding
E163	T47	2.83	
A167	A107	2.84	
**Y261**	**E35**	**2.89**	**Active Site**
H139	A101	2.96	
D225	K33	2.96	
Y255	R114	2.97	
S93	R61	2.98	
A167	N59	2.89	
Q166	N103	3.03	
E88	R61	3.08	
T165	W62	3.10	PG Binding
T171	N46	3.14	
**S169**	**D52**	**3.16**	**Active Site**
S169	N46	3.16	
K137	N103	3.17	
E256	R114	3.18	

^a^ Interactions based on PyMOL script list_contacts.py [95].

^b^ Bolded lines indicate amino acids determined to be important for RsiV binding to lysozyme.

### Multiple regions of RsiV are involved in binding lysozyme

Previous work showed that the extracellular domain of RsiV binds lysozyme and *in vitro* site-1 cleavage of RsiV by signal peptidase required the presence of lysozyme [12]. We hypothesized that activation of σ^V^ is dependent upon binding of RsiV to lysozyme. To directly test this model, we sought to identify mutants of RsiV that are unable to bind lysozyme and determine if they are also unable to activate σ^V^. We began by constructing mutations of highly conserved residues of RsiV which interacted with lysozyme. We then purified each of these mutant proteins and tested their ability to bind lysozyme *in vitro*.

**Fig 3:**
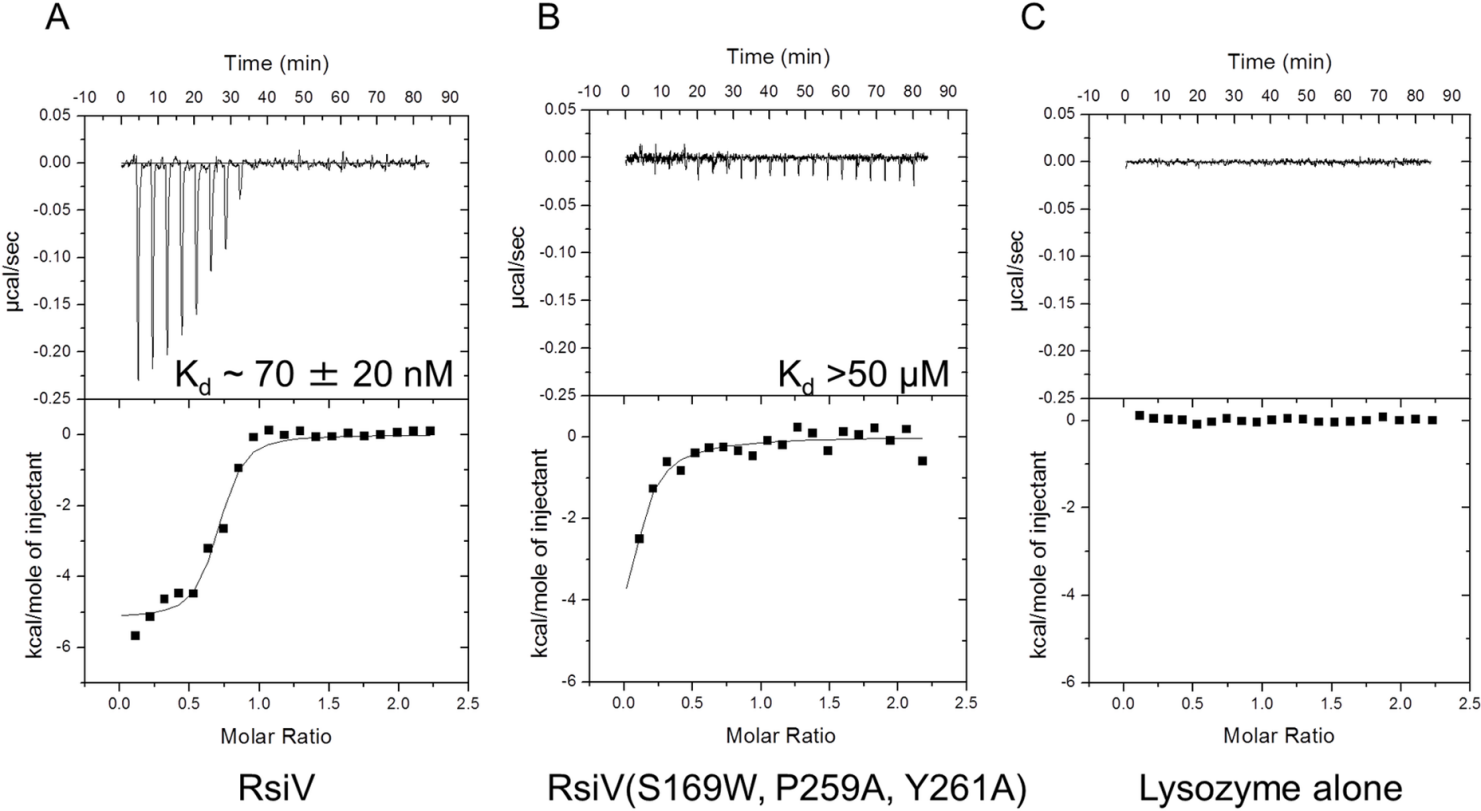
RsiV^S169W, P259A, Y261A^ is unable to bind lysozyme. Representative run of isothermal titration calorimetry (ITC) experiments conducted with **A**. 6xHis-2xFlag-RsiV^59-285^ and HEW lysozyme or **B**. 6xHis-2xFlag-RsiV^59-285, S169W, P259A, Y261A^ and HEW lysozyme. An interaction was seen with the WT construct, but not with the triple mutant. **C**. HEW lysozyme only injection control. RsiV was loaded in the cell at a concentration of 0.01 mM and lysozyme was loaded in the syringe at a concentration of 0.1 mM.

As previously reported we found that wild type RsiV^59-285^ binds lysozyme with a *K*_*d*_ of 70 nM (Table 3) [12]. We found the mutant proteins RsiV^59-285, P259A^ and RsiV^59-285, Y261A^ were able to bind lysozyme as well as wild type (Table 3 and S6 Fig). In contrast we found that RsiV^59-285, S169W^ showed a reduced affinity for lysozyme, but not a complete loss of binding as compared to the lysozyme only control. (Table 3, S6 Fig, and Fig 3C). Since RsiV makes multiple contacts with lysozyme (Table 2), we hypothesized that a single mutation may not be sufficient to completely block lysozyme binding, thus we analyzed the ability of RsiV double and triple mutants to bind lysozyme. The double mutants RsiV^59-285, P259A Y261A^ and RsiV^59-285, S169W P259A^ were able to bind lysozyme, but with a ~100 fold and ~3 fold less affinity, respectively (Table 3 and S6 Fig). The RsiV^59-285, S169W, P259A, Y261A^ triple mutant behaved similar to a lysozyme only injected control, suggesting a near complete loss of lysozyme binding activity (Table 3 and Fig 3B and 3C).

**Table 3:** Affinity of RsiV mutants for lysozyme.

Mutation	K_d_ Value
RsiV	70 ± 20 nM
RsiV^S169W, P259A, Y261A^	>50 μM
RsiV^S169W^	>50 μM
RsiV^P259A, Y261A^	600 ± 400 nM
RsiV^P259A^	30 ± 10 nM
RsiV^S169W, P259A^	269 ± 50 nM
RsiV^Y261A^	10 ± 5 nM
RsiV^S169W, Y261A^	>50 μM

Circular dichroism (CD) analysis was used to determine if the RsiV^59-285, S169W, P259A, Y261A^ triple mutant was simply unable to properly fold. We determined the RsiV^59-285, S169W, P259A, Y261A^ triple mutant retains similar α-helix and β-sheet conformation as wild type suggesting the overall secondary structure is only slightly altered (S7 Fig). Taken together these data suggest that multiple regions are involved in RsiV binding to lysozyme and these contribute to the high affinity this receptor displays for its ligand.

### Lysozyme binding is required for σ^V^ activation

**Fig 4:**
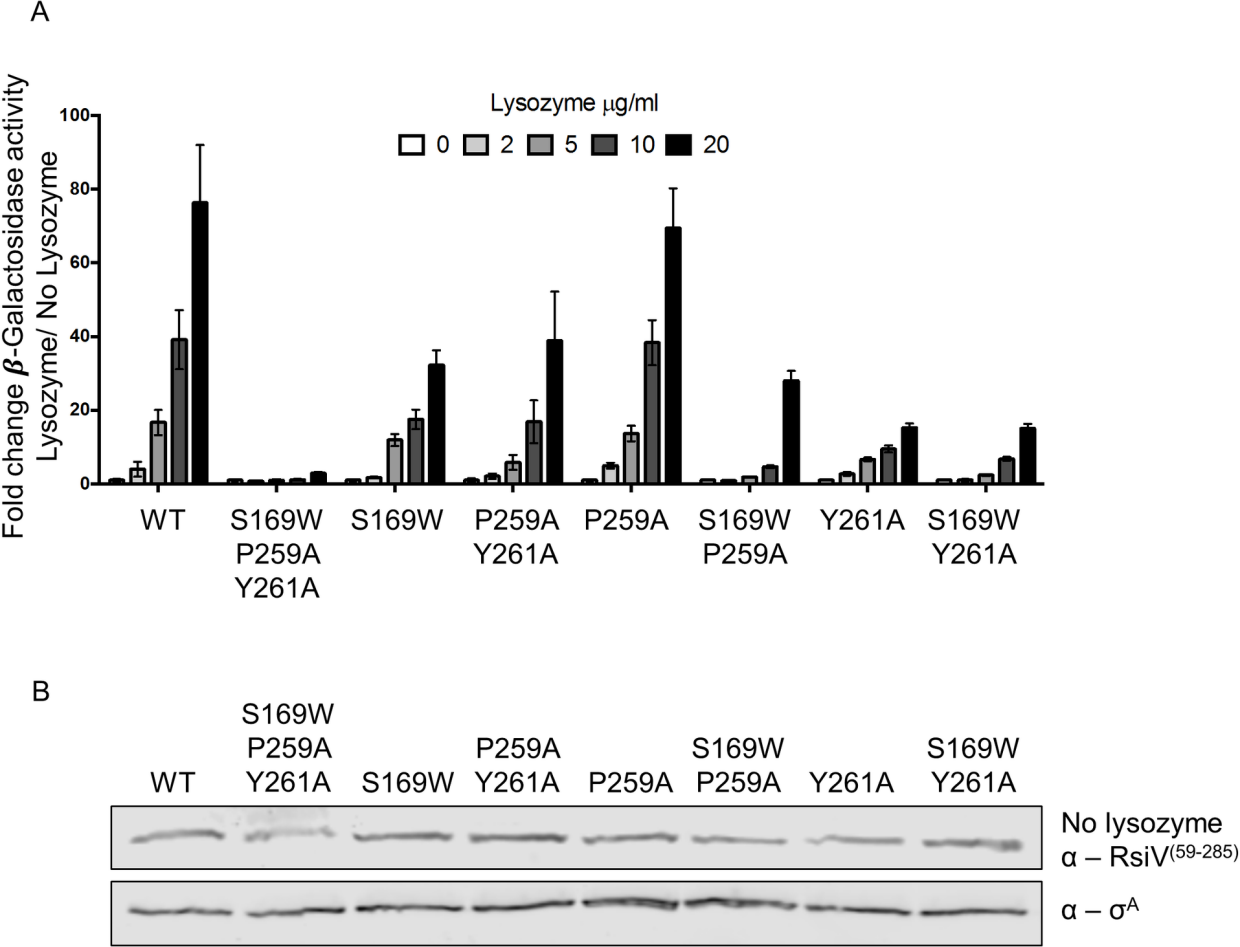
RsiV binding to lysozyme is required for σ^V^ activation. **A.** The effect of RsiV mutants on σ^V^ activation using a P_*sigV*_*-lacZ* reporter assay. *B*. *subtilis* strains CDE1546 (WT), JLH1473 (S169W, P259A, Y261A), JLH1474 (S169W), JLH1477 (P259A, Y261A), JLH1527 (P259A), JLH1536 (S169W, P259A), JLH1476 (Y261A) and JLH1538 (S169W, Y261A) were grown to mid log and then 20 μl were spotted on LB plates with various concentrations of lysozyme (0, 2, 5, 10, 20 μg/ml). Plates were incubated 37°C for 6 hours and then β-galactosidase assays were performed. **B.** Immunoblot analysis of RsiV mutant protein levels. An aliquot of 1 ml was taken from each strain before spotting. The aliquot was pelleted and resuspended in 50 μl sample buffer. Samples were immunoblotted with anti-RsiV^59-285^ and expression levels were compared using the Li-Cor software ImageStudio.

We hypothesize that RsiV binding to lysozyme is required for σ^V^ activation. Therefore we analyzed the effect of RsiV mutants defective in lysozyme binding on σ^V^ activation. The single, double and triple mutant *rsiV* alleles were integrated into the native chromosomal site by homologous recombination to allow for σ^V^ dependent regulation of *rsiV* expression. We confirmed that all of these strains produced similar basal amounts of RsiV, in the absence of lysozyme, by immunoblot (Fig 4B). This demonstrates that these mutant proteins are stable and are not altered for σ^V^ activation in the absence of lysozyme.

The activation of σ^V^ in the resulting strains was determined by exposure to increasing concentrations of lysozyme (0, 2, 5, 10, 20 μg/ml) by measuring expression from the P_*sigV*_-*lacZ* reporter. We found that the triple mutant *rsiV*^*S169W*, *P259A*, *Y261A*^ abolished σ^V^ activation (Fig 4A). As previously mentioned, immunoblot analysis shows that RsiV^S169W, P259A, Y261A^ was produced at similar levels to wild type RsiV (Fig 4B). So this lack of activation is not simply due to insufficient or unstable protein. CD analysis suggests that RsiV^S169W, P259A, Y261A^ is not simply improperly unfolded (S7 Fig). In addition, if RsiV^S169W, P259A, Y261A^ were unfolded we would predict that it would be constitutively degraded via RIP.

We also tested the role of the single and double mutants of RsiV and found they fell into two distinct groups: 1) mutants that had near wild type levels of σ^V^ activation; RsiV^P259A^ and RsiV^Y261A^ (Fig 4A), and 2) mutants that reduce, but do not abolish, σ^V^ activation; RsiV^S169W^, RsiV^P259A, Y261A^, RsiV^S169W, P259A^, and RsiV^S169W, Y261A^ (Fig 4A). We found that the reduced activation of σ^V^ in RsiV mutants, RsiV^S169W^, RsiV^P259A, Y261A^, RsiV^S169W, P259A^, and RsiV^S169W, Y261A^, was dependent upon lysozyme concentration (Fig 4). At higher lysozyme concentrations these mutants were still able to activate σ^V^. All of these data are consistent with the model in which these mutants show lower affinity for lysozyme and thus activate σ^V^ only at higher lysozyme concentrations. Thus, we have demonstrated the overall the ability of the RsiV mutants to activate σ^V^ correlates with their ability to bind lysozyme.

### Lysozyme binding to RsiV is required for site-1 cleavage of RsiV

Activation of σ^V^ requires the degradation of RsiV via RIP [11,12]. Thus we sought to determine if RsiV mutants which blocked or decreased binding of RsiV to lysozyme also blocked RIP of RsiV in response to lysozyme. We expressed wild type or mutant forms of RsiV, using an IPTG inducible promoter (P_*hs*_) in a strain of *B*. *subtilis* with a Δ*sigVrsiV*::*kan* mutation to break the auto-regulation controlling RsiV production. Control experiments revealed that when all strains were grown in 1 mM IPTG RsiV^P259A^ and RsiV^S169W Y261A^ were produced at about 6-fold higher levels compared to the other strains (S8A Fig). We found strains producing RsiV^P259A^ and RsiV^S169W Y261A^ when grown in media with 0.1 mM IPTG resulted in production of similar levels of RsiV compared to other strains grown in the presence of 1 mM IPTG (S8B Fig). Thus for these experiments the concentration of IPTG used differed in order to produce the RsiV mutant proteins at similar levels. Cells producing wild type or mutant forms of RsiV were exposed to a gradient of lysozyme concentrations (0, 0.01, 0.1, 1, 2 μg/ml) for 10 minutes and cleavage of RsiV was monitored by immunoblot using an RsiV specific antibody.

**Fig 5:**
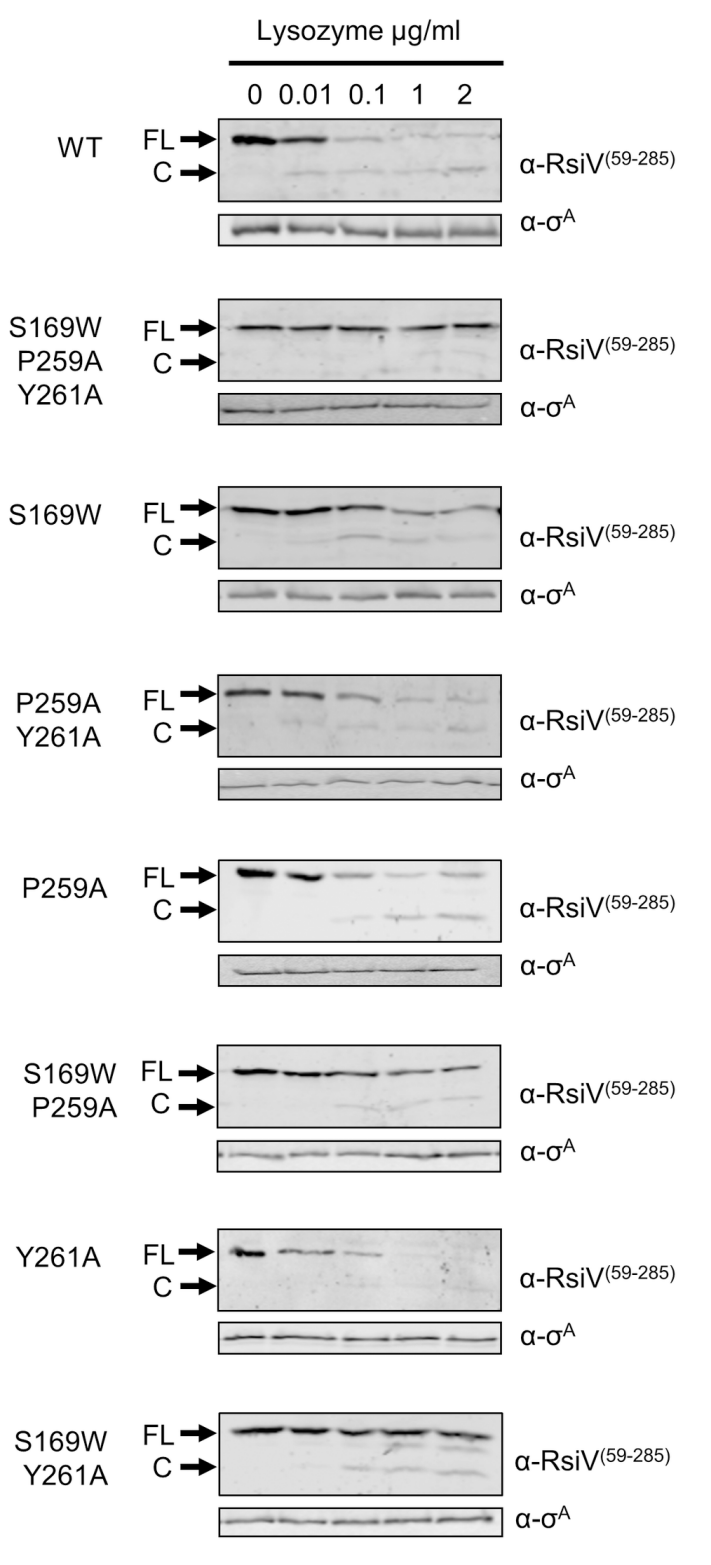
RsiV binding to lysozyme is required for site-1 cleavage of RsiV. Site-1 cleavage of RsiV mutants in response to increasing concentration of lysozyme. Overnight *B*. *subtilis* strains were grown to OD_600_ of 1 in LB IPTG. Strains were divided into 1.5 ml aliquots and incubated with increasing concentrations of lysozyme (0, 0.01, 0.1, 1, and 2 μg/mL) for 10 minutes. Following lysozyme exposure, cells were pelleted, resuspended in 100 μl sample buffer, and immunoblotted with anti-RsiV and anti-σ^A^. The blots are labelled on the right with either α-RsiV^59-285^ or α-σ^A^. The FL arrow denotes full length RsiV. The C arrow denotes cleaved extracellular domain of RsiV. WT = JLH402; S169W P259A Y261A refers to JLH1312; S169W refers to JLH1271; P259A Y261A refers to JLH1343; P259A refers to JLH1481; S169W P259A refers to JLH1326; Y261A refers to JLH1342; S169W Y261A refers to JLH1504.

As previously observed, we found that wild type RsiV is nearly completely cleaved at site-1 in 10 minutes in the presence of as little as 0.1 μg/ml of lysozyme (Fig 5) [11,12]. In contrast the RsiV triple mutant (RsiV^S169W, P259A, Y261A^), which was unable to bind lysozyme and activate σ^V^, was not degraded even at the highest concentration of lysozyme (2 μg/ml) (Fig 5). This strongly suggests it can no longer sense lysozyme. Similar to the effects on σ^V^ activation, we observed the RsiV single and double mutants displayed intermediate phenotypes compared to wild type RsiV (Fig 5). RsiV^P259A^ and RsiV^Y261A^ were degraded similar to wild type RsiV (Fig 5). Mutants RsiV^S169W^, RsiV^P259A, Y261A^, RsiV^S169W P259A^, and RsiV^S169W Y261A^ are degraded in the presence of the highest concentration of lysozyme (Fig 5). However at lower concentrations (1 μg/ml and 0.1 μg/ml) the mutant RsiV remains intact, while wild type RsiV is rapidly degraded. Taken together these data support a true receptor-ligand signaling model that requires lysozyme binding to RsiV for activation of σ^V^.

### Binding of RsiV to lysozyme is required for lysozyme resistance of *B*. *subtilis*

To determine if binding of RsiV to lysozyme was required for lysozyme resistance we performed assays to measure the minimum inhibitory concentration (MIC) by lysozyme on strains with the single, double and triple mutants integrated into the native chromosomal locus. As previously reported, we found the Δ*sigVrsiV* mutant showed increased sensitivity to lysozyme compared to wild type *B*. *subtilis* (Table 4) [5]. As predicted from the data above, we found that that the cells producing the RsiV triple mutant (RsiV^S169W, P259A, Y261A^) were nearly as sensitive to lysozyme as the Δ*sigVrsiV* (Table 4). Similarly, cells producing either single or double mutant RsiV proteins showed intermediate resistance to lysozyme, correlating with their ability to activate σ^V^ and bind lysozyme (Table 4). Taken together this demonstrates that an inability of RsiV to bind lysozyme results in increased sensitivity to lysozyme.

**Table 4:** Sensitivity of cells producing different RsiV mutants to lysozyme.

Mutation	Strain	Lysozyme MIC
WT	CDE1546	15
Δ*sigVrsiV*	JLH1936	5
RsiV^S169W, P259A, Y261A^	JLH1412	7.5
RsiV^S169W^	JLH1413	10
RsiV^P259A, Y261A^	JLH1372	15
RsiV^P259A^	JLH1380	10
RsiV^S169W, P259A^	JLH1536	7.5
RsiV^Y261A^	JLH1510	15
RsiV^S169W, Y261A^	JLH1507	15

### RsiV is an inhibitor of lysozyme activity *in vitro*

**Fig 6:**
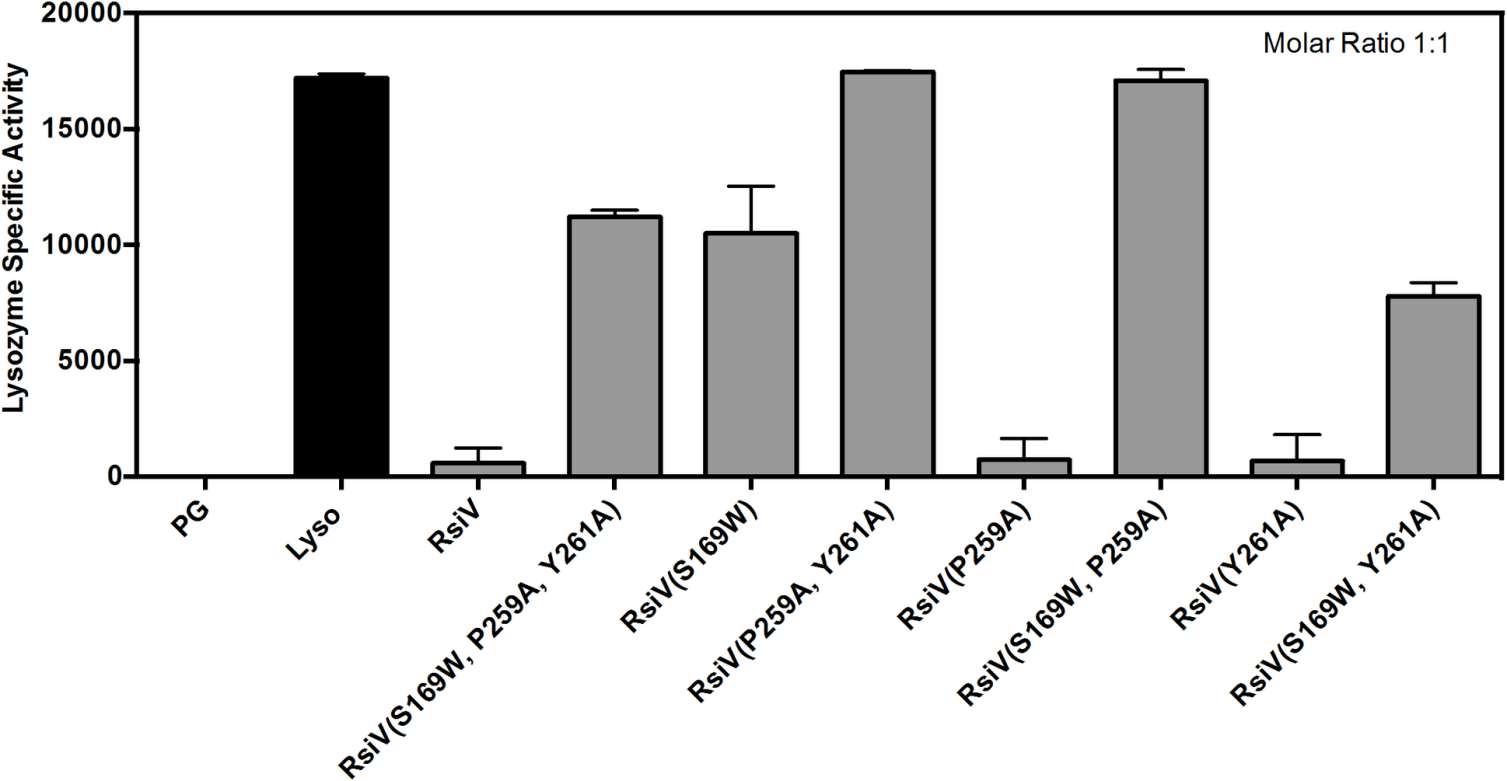
RsiV mutants that cannot bind lysozyme are less efficient at inhibiting lysozyme muramidase activity. Peptidoglycan from *M*. *lysodekticus* was combined with lysozyme (20 μg/ ml) and purified RsiV or RsiV mutants at a molar ratio of 1:1 with lysozyme. The OD_450_ was monitored every minute for 30 minutes to determine lysozyme specific activity as previously described [41].

Analysis of the RsiV-lysozyme co-crystal structure revealed RsiV binds in the enzymatic pocket of lysozyme containing the active site residues D52 and E35 as well as several residues required for binding peptidoglycan [39,40]. Thus we hypothesized RsiV could act as an inhibitor of lysozyme activity. We assayed the activity of lysozyme in the presence and absence of RsiV using a standard lysozyme activity assay [41]. Wild type RsiV^59-285^ almost completely inhibits lysozyme activity at a 1:1 molar ratio, suggesting RsiV is an effective lysozyme inhibitor (Fig 6). We then compared the ability each of the RsiV mutants to inhibit lysozyme activity. The mutants RsiV^59-285^, ^P259A^ and RsiV^59-285, Y261A^ retain the ability to inhibit lysozyme to the same degree as wild type RsiV (Fig 6). The mutants RsiV^59-285, S169W^, RsiV^59-285, S169W Y261A^, RsiV^59-285, S169W P269A^, RsiV^59-285, P259A and Y261A^, and RsiV^59-285, S169W, P259A, Y261^ were not able to inhibit lysozyme as well as wild type (Fig 6). We found that at higher concentrations, 2 and 4 molar ratios, the mutants RsiV^59-285, S169W^ and RsiV^59-285, S169W Y261A^ almost completely abolish lysozyme activity (S9 Fig). However, the mutants RsiV^59-285, S169W P269A^, RsiV^59-285, P259A and Y261A^, and RsiV^59-285, S169W, P259A, Y261^ showed significant decreases in the ability to inhibit lysozyme activity even at a molar ratio of 4:1 (S9 Fig). Together the data suggest lysozyme inhibition correlates with ability of RsiV to bind lysozyme (Fig 6 and S9 Fig).

To determine if RsiV was produced at a level sufficient to inhibit lysozyme activity we determined the levels of RsiV in the absence of lysozyme and estimated RsiV levels in the presence of lysozyme. In wild type *B*. *subtilis* there are approximately 220 molecules of RsiV per cell (S10 Fig). We found that the cells harboring the IPTG inducible construct of *rsiV* produce ~7420 molecules per cell. Since RsiV is cleaved upon binding lysozyme and released into the supernatant it was more difficult to determine RsiV protein levels in the presence of lysozyme. Therefore, we estimated the levels of RsiV protein by comparing the levels of *rsiV* transcript in uninduced and induced wild type cells to cells harboring the IPTG inducible construct of *rsiV*. The *rsiV* transcript was induced ~100 fold upon addition of 1.25 μg/ml of lysozyme in wild type cells (S2 Table, [5,6]). We found that *rsiV* transcript was ~44 fold higher in cells harboring the IPTG inducible construct of *rsiV* compared to uninduced wild type cells (S2 Table). Thus we estimate there are approximately 16700 molecules of RsiV per cell when induced with 1.25 μg/ml of lysozyme which is the equivalent of ~52600 molecules of lysozyme per cell (S2 Table). This suggests RsiV is produced at levels high enough to function as a competitive inhibitor of lysozyme. We sought to determine if RsiV contributes to lysozyme resistance *in vivo* by identifying the minimum inhibitory concentration (MIC) of cells producing RsiV from an exogenous promoter integrated at *amyE* in the absence of σ^V^. We observed that the *ΔsigVrsiV* mutant strain was 3-fold more sensitive to lysozyme compared to wild type *B*. *subtilis* (Table 5). Cells producing RsiV in a *ΔsigVrsiV* are 1.5 fold more resistant to lysozyme than the *ΔsigVrsiV* parent strain (Table 5), suggesting RsiV inhibition of lysozyme activity contributes to σ^V^-induced lysozyme resistance.

**Table 5:** RsiV provides lysozyme resistance *in vivo*.

Mutation	Strain	Lysozyme MIC
WT	PY79	15
Δ*sigVrsiV*	CDE1563	5
Δ*sigVrsiV P*_*hs*_*-rsiV*^*+*^	JLH402	7.5

## Discussion

### RsiV is a receptor for lysozyme

We determined the x-ray structure of RsiV and lysozyme complex at 2.3 Å. Using the co-structure we demonstrate that RsiV is a receptor for lysozyme and this interaction is required for σ^V^ activation. Our results indicated that due to the extensive contacts between RsiV and lysozyme a single mutation was not sufficient to block binding or σ^V^ activation. We found that several amino acid residues, S169 and P259 Y261, are critical for RsiV binding to lysozyme. These residues are located in two distinct loops of RsiV that protrude from the top of the structure and interact with lysozyme. Interestingly these residues are highly conserved amongst RsiV homologs. Our data indicates that S169 has a greater impact on the binding than P259 and Y261, but all contribute to binding. While the majority of the phenotypes are consistent with the each RsiV mutant there are some outliers. For example, the Y261A mutation has a more dramatic effect on σ^V^ activity than it appears to have on lysozyme binding or RsiV cleavage. The reason for this remains unclear. Overall however, site directed mutagenesis of residues S169, P259, and Y261 showed a correlation between decreased σ^V^ activity, loss of RsiV degradation, and an inability to bind lysozyme.

Our model that RsiV binding to lysozyme is required for σ^V^ activation is supported by multiple pieces of evidence. First, the solved co-structure of RsiV and lysozyme, revealed an intimate interaction between these proteins coordinated by contacts through multiple amino acid residues (Fig 1). Second, RsiV and lysozyme bind with high affinity (Table 2 and [12]). Third, we previously found site-1 cleavage of RsiV by signal peptidase occurred only in the presence of lysozyme [12]. Fourth, here we demonstrate that mutants of RsiV deficient in lysozyme binding also fail to activate σ^V^.

**Fig 7:**
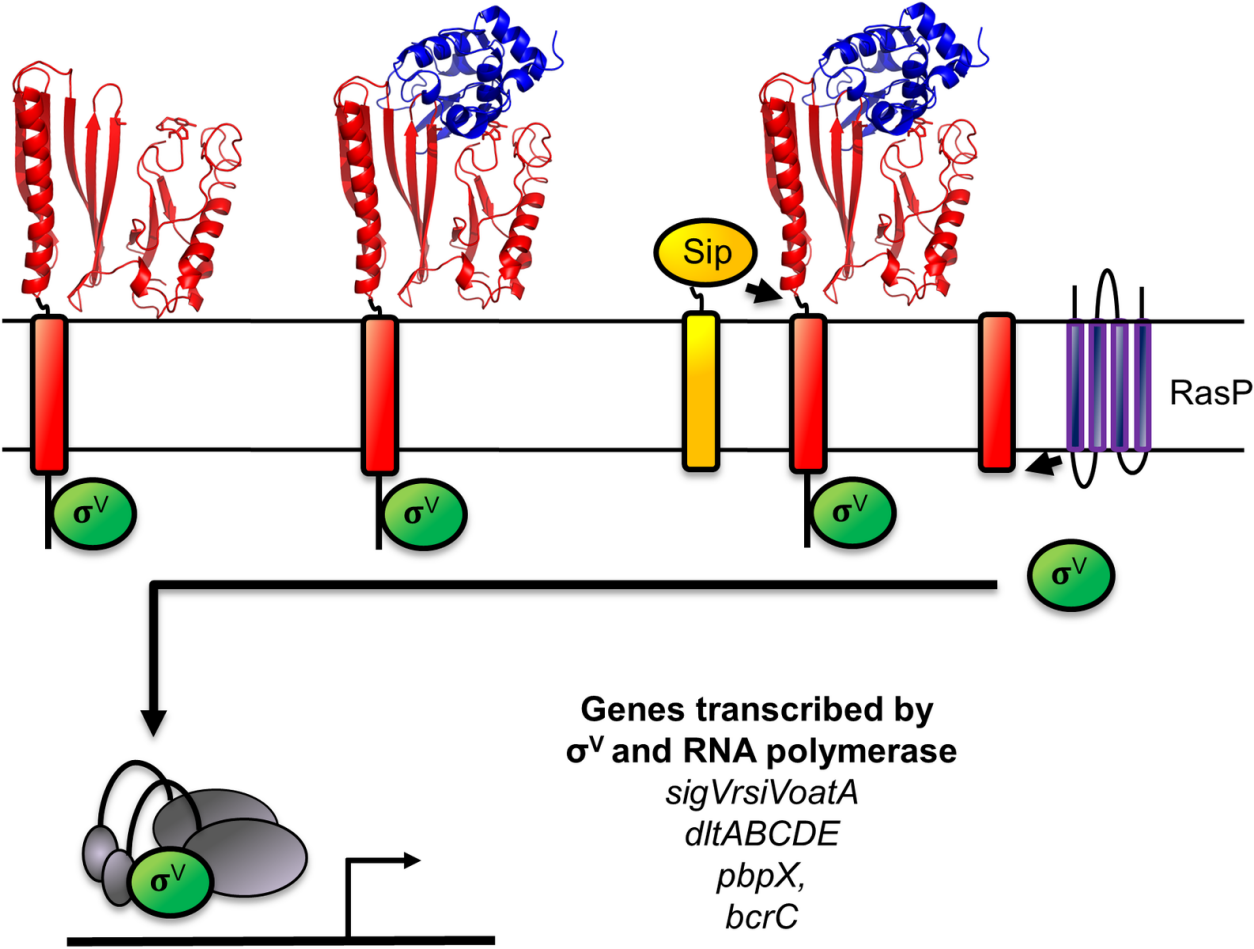
Model of lysozyme-mediated σ^V^ activation. Shown in green is σ^V^, the anti-σ factor RsiV is red (the cartoon portions represent regions of RsiV for which the structure has not been determined (1–75) while the structure of RsiV is shown representing (76–285)), the signal peptidase (Sip) is yellow, the site-2 protease (RasP) is purple and lysozyme is in blue. In the absence of lysozyme RsiV inhibits σ^V^ activity and is resistant to cleavage by signal peptidase. Upon binding to lysozyme RsiV becomes sensitive to signal peptidase and is cleaved at site-1. The site-1 cleaved RsiV then becomes a substrate for the site-2 protease RasP. The cytoplasmic portion of RsiV then becomes a substrate for cytosolic proteases which free σ^V^ to interact with RNA polymerase and transcribe target genes which are responsible for lysozyme resistance [5,6].

Our data indicate that lysozyme binds RsiV some distance from the site-1 cleavage site (Fig 7). Thus we propose the following model for how binding of RsiV to lysozyme controls σ^V^ activation. In the absence of lysozyme, the cell normally makes a low basal level of σ^V^ and RsiV. Binding of lysozyme to RsiV results in a conformational change in RsiV which allows signal peptidase to recognize the previously masked cleavage site. Once site-1 cleavage of RsiV occurs the truncated RsiV can be cleaved by the site-2 protease RasP, leading to activation of σ^V^. Once activated, σ^V^ is able increase transcription of the *sigV-rsiV-oatA* operon. This leads to increased production of RsiV which continues to bind free lysozyme and increases the level of free σ^V^. This auto-regulatory loop allows the bacteria to rapidly respond and to produce more RsiV until all the lysozyme in the environment is sequestered by RsiV. At which point unbound RsiV builds up and inhibits further σ^V^ activation. This is analogous to the recently proposed model for how the ABC transporter BceAB and the two component signal transduction system BceSR control bacitracin resistance in *B*. *subtilis* [42]. In this system BceAB is required for resistance to bacitracin and acts as the sensor for bacitracin presence by controlling BceS activity [42]. Similarly, a “Just-in-time” model of regulation was proposed to explain how the immunity protein SdpI was involved in controlling expression of resistance to the cannibalism peptide [43]. A key similarity in each of these systems is the levels of the sensor are controlled by the regulator as well.

### RsiV is a member of a new family of lysozyme inhibitors

The co-crystal structure revealed that RsiV binds lysozyme by interacting with the enzymatic pocket required for muramidase activity. Several lysozyme inhibitors have been identified in Gram-negative bacteria, however, few if any dedicated lysozyme inhibitors have been identified in Gram-positive bacteria [44]. A few virulent strains of group A streptococci produce SIC (Streptococcal inhibitor of complement), which inhibits antimicrobial peptides and proteins of the innate immune response [45]. However, SIC only binds lysozyme relatively weakly, Kd = 85 μM, while binding to several other antimicrobial factors with higher affinity [45,46]. In contrast, the interaction between RsiV and lysozyme has a high affinity in the same range as Gram-negative inhibitors (nm range) [47]. Thus RsiV may represent the first specific inhibitor of lysozyme activity in Gram-positive bacteria. Lysozyme inhibitors from Gram-negative bacteria have been easier to isolate because they are soluble and located in the periplasm. It may be that other Gram-positive bacteria encode lysozyme inhibitors however association with the membrane has made them more difficult to identify.

Known lysozyme inhibitors include: Ivy (inhibitor of vertebrate lysozyme) identified from *E*.*coli* [48], MliC (membrane lysozyme inhibitor) from *E*.*coli*, *Pseudomonas aeruginosa*, and *Salmonella enteritidis* [47] and PliC (periplasmic lysozyme inhibitor) from *Proteus mirabilis* [49]. Ivy and MliC, which inhibit C-type lysozymes have been co-crystalized with lysozyme, revealing specific loops that interact with the catalytic domains [44]. Ivy inserts a 5 residue loop into the active site of lysozyme, which allows residue H60 to makes contact with E35 and D52 of lysozyme [50] MliC inserts two conserved loops into the active site of lysozyme. This allows S89 to interact with lysozyme D52, and K103 to interact with lysozyme E35 and D52 [51]. RsiV appears to utilize a similar mechanism of inhibition by inserting two loops into the active site of lysozyme, which allows S169 to interact with D52, and Y261 to interact with E35 (Table 2). This suggests that proteins with very different structures use similar mechanisms to occlude the active site of lysozyme.

### RsiV is a multi-function protein that coordinates σ^V^ activation and lysozyme resistance

We have shown the anti-σ factor RsiV is a multifunctional protein. RsiV inhibits σ^V^ activity in the absence of stress, senses the inducing signal lysozyme, and inhibits lysozyme function by binding in the active site. The main role of most anti-σ factors is to inhibit the activity of the ECF σ factor. However some anti-σ factors are involved in sensing the inducing signal [3]. For example the anti-σ factors in several organisms respond to oxidative stress which is thought to occur via modification of cysteine residues in the anti-σ factor [3,52–54]. In *Clostridium thermocellum* several anti-σ factors contain carbohydrate binding domains [55–58]. However it is not known how binding of the carbohydrates to the anti-σ factor leads to activation of the σ factor. Another example of polysaccharide sensing by anti-σ factors is hypothesized to occur in *Bacteroides thetaiotaomicron*. In this system it is thought the ECF σ factors are responsible for responding to polysaccharides in the intestinal tract [4,59,60].

Most bacteria respond to lysozyme stress by either modifying their peptidoglycan or making a lysozyme inhibitor [44,61]. Interestingly our data indicate that activation of σ^V^ induces both activities. RNA polymerase containing σ^V^ transcribes the *sigV-rsiV-oatA* operon. The increased production of RsiV allows the cell to bind free lysozyme and inhibit its activity. In addition, increased production of OatA, an *O*-acetyltransferase that adds an acetyl group to MurNac of peptidoglycan, increases resistance to lysozyme [5,15]. Similarly σ^V^ homologs in *C*. *difficile* and *E*. *faecalis* are also required for transcription of a gene encoding a peptidoglycan modifying enzyme that removes an acetyl group from GlcNac [8,62]. The deacetylation of GlcNac also increases resistance to lysozyme [8,10,63]. Thus it appears that the σ^V^-RsiV signal transduction system is able to increase lysozyme resistance using two distinct mechanisms. RsiV responds by inhibiting lysozyme activity via direct binding to the lysozyme active site. At the same time this binding event triggers the proteolytic cascade that results in increased levels of free σ^V^. Activation of σ^V^ leads to production of RsiV which continues to bind free lysozyme and production of a peptidoglycan modification enzyme which increases lysozyme resistance. Thus σ^V^ can induce immediate lysozyme resistance by inhibiting lysozyme activity through RsiV and a longer term stress response by modifying peptidoglycan. This could provide both transient protection and prime the cells for continued growth in an environment where future lysozyme stress on their cell wall might be likely.

### Unanswered questions

It is not known when or if vegetative *B*. *subtilis* encounters lysozyme outside of the laboratory. *B*. *subtilis* is widely considered a soil organism and is often found associated with plant roots [64–67]. Interestingly, as a generally regarded as safe (GRAS) organism *B*. *subtilis* is also used in a variety of industrial and agricultural processes which may contribute to diverse environmental exposure. Recent studies have found *B*. *subtilis* can be isolated from the intestinal tracts of a variety of organisms which produce C-type lysozyme including *Drosophila melagnoster*, chickens, mice and humans [61,68–71]. It is unclear how association with the intestinal tract of a diverse number of organisms developed, likely consumption of soil, but perhaps *B*. *subtilis* has additional environmental niches beyond life in the soil. It is tempting to hypothesize that lysozyme resistance could be an important trait required for colonization of or survival though the intestinal tract of higher organisms.

It is also not known what types of lysozymes *B*. *subtilis* encounters in the environment. Another possibility is that σ^V^ is required for resistance to multiple types of lysozyme. Previous work demonstrated that the muramidase mutanolysin was unable to activate σ^V^ [6] and RsiV did not bind mutanolysin [12]. The structure of the bacterial produced mutanolysin is quite distinct from C-type lysozymes like hen egg white lysozyme [72,73]. In contrast G-type and I-type lysozymes produced by eukaryotes are structurally similar to C-type lysozyme and likely the result of divergent evolution [74,75]. However, at this time it is not known if RsiV can bind to G-type or I-type lysozymes. One interesting point to note however is many inhibitors of one class of lysozyme fail to inhibit other classes of lysozyme suggesting that they are distinct enough to not be recognized by the same inhibitor [44].

## Materials and Methods

### Strain construction

All plasmid constructs are listed in Table 6 were confirmed by DNA sequencing (Iowa State DNA sequencing facility). All *B*. *subtilis* strains are isogenic derivatives of PY79, a prototrophic derivative of *B*. *subtilis* strain 168 [76] and are listed in Table 7. *B*. *subtilis* competent cells were prepared by the one-step method previously described [77].

**Table 6:** Plasmid list.

Plasmid	Genotype	Reference^a^
pDR111	*amyE* P_*hs*_ *specR ampR* P_hs_	[78]
pCE292	pDR111 *amyE* P_*hs*_ *specR ampR* P_*hs*_-RfA (*ccdB*^*+*^ *camR*)	[7]
pJH386	pDR111 *amyE* P_*hs*_ *specR ampR* P_*hs*_-*rsiV(S169W*, *P259A*, *Y261A)*	
pJH375	pDR111 *amyE* P_*hs*_ *specR ampR* P_*hs*_-*rsiV(S169W)*	
pJH436	pDR111 *amyE* P_*hs*_ *specR ampR* P_*hs*_-*rsiV(P259A)*	
pJH397	pDR111 *amyE* P_*hs*_ *specR ampR* P_*hs*_-*rsiV(Y261A)*	
pJH398	pDR111 *amyE* P_*hs*_ *specR ampR* P_*hs*_-*rsiV(P259A*, *Y261A)*	
pJH405	pDR111 *amyE* P_*hs*_ *specR ampR* P_*hs*_-*rsiV(S169W*, *P259A)*	
pJH439	pDR111 *amyE* P_*hs*_ *specR ampR* P_*hs*_-*rsiV(S169W*, *Y261A)*	
pMAD	*bla ermC* ori pBR322 ori pE194^ts^	[98]
pJH406	pMAD *sigV*^+^*rsiV*^*S169W*, *P259A*, *Y261A*^	
pJH381	pMAD *sigV*^+^*rsiV*^*S169W*^	
pJH403	pMAD *sigV*^+^*rsiV*^*P259A*^	
pJH438	pMAD *sigV*^+^*rsiV*^*Y261A*^	
pJH404	pMAD *sigV*^+^*rsiV*^*P259A*, *Y261A*^	
pJH405	pMAD *sigV*^+^*rsiV*^*S169W*, *P259A*^	
pJH437	pMAD *sigV*^+^*rsiV*^*S169W*, *Y261A*^	
pJH402	pMAD *sigV*^+^*rsiV*^*H137A K139A*^	
pEntrD-topo	*kanR*	Invitrogen
pJH423	pEntrD-topo *‘2xflag-rsiV*^*59-285 S169W*, *P259A*, *Y261A*^ *kanR*	
pJH421	pEntrD-topo *‘2xflag-rsiV*^*59-285 S169W*^ *kanR*	
pJH426	pEntrD-topo *‘2xflag-rsiV*^*59-285 P259A*^ *kanR*	
pJH424	pEntrD-topo *‘2xflag-rsiV*^*59-285 Y261A*^ *kanR*	
pJH425	pEntrD-topo *‘2xflag-rsiV*^*59-285 P259A*, *Y261A*^ *kanR*	
pJH446	pEntrD-topo *‘2xflag-rsiV*^*59-285 S169W*, *P259A*^ *kanR*	
pJH448	pEntrD-topo *‘2xflag-rsiV*^*59-285 S169W*, *Y261A*^ *kanR*	
pDEST17	*P*_*T7*_*6xhis cat ccdB ampR* ori pBR322	Invitrogen
pKWB201	pDEST17 P_T7_*-6xhis-2xflag*-*rsiV*^59-285^ *ampR*	[11,12]
pJH429	pDEST17 P_T7_*-6xhis-2xflag*-*rsiV*^59-285^, ^*S169W*, *P259A*, *Y261A*^ *ampR*	
pJH427	pDEST17 P_T7_*-6xhis-2xflag*-*rsiV*^59-285, *S169W*^ *ampR*	
pJH450	pDEST17 P_T7_*-6xhis-2xflag*-*rsiV*^59-285, *P259A*^ *ampR*	
pJH430	pDEST17 P_T7_*-6xhis-2xflag*-*rsiV*^59-285 *Y261A*^ *ampR*	
pJH431	pDEST17 P_T7_*-6xhis-2xflag*-*rsiV*^59-285, *P259A*, *Y261A*^ *ampR*	
pJH447	pDEST17 P_T7_*-6xhis-2xflag*-*rsiV*^59-285, *S169W*, *P259A*^ *ampR*	
pJH453	pDEST17 P_T7_*-6xhis-2xflag*-*rsiV*^59-285, *S169W*, *Y261A*^ *ampR*	
pMRLB.12	pET15b P P_T7_*-6xhis-rv1980c ampR*	BEI Resources

^a^ This study, unless otherwise noted.

**Table 7:** Strain list.

Strain	Genotype	Reference^a^
***E*.*coli***		
BL21(DE3)	*E*. *coli fhuA2 [lon] ompT gal (λ DE3) [dcm] ΔhsdS λDE3 (sBamHIo ΔEcoRI-B int*::*(lacI*::*PlacUV5*::*T7 gene1) i21 Δnin5)*	
***B*. *subtilis***		
PY79	Prototrophic derivative of *B*. *subtilis* 168	[76]
CDE1546	PY79 *pyrD*::P_*sigV*_*-lacZ* (*cat*)	[5]
CDE1563	PY79 Δ*sigVrsiV*::*kan*	[11]
JLH402	PY79 *amyE*::P_*hs*_*-rsiV (spec)* Δ*sigVrsiV*::*kan*	[11]
JLH1312	PY79 *amyE*::P_*hs*_*-rsiV*^S169W,P259A, Y261A^ *(spec)* Δ*sigVrsiV*::*kan*	
JLH1271	PY79 *amyE*::P_*hs*_*-rsiV*^S169W^ *(spec)* Δ*sigVrsiV*::*kan*	
JLH1481	PY79 *amyE*::P_*hs*_*-rsiV*^P259A^ *(spec)* Δ*sigVrsiV*::*kan*	
JLH1342	PY79 *amyE*::P_*hs*_*-rsiV*^Y261A^ *(spec)* Δ*sigVrsiV*::*kan*	
JLH1343	PY79 *amyE*::P_*hs*_*-rsiV*^P259A, Y261A^ *(spec)* Δ*sigVrsiV*::*kan*	
JLH1326	PY79 *amyE*::P_*hs*_*-rsiV*^S169W, P259A^ *(spec)* Δ*sigVrsiV*::*kan*	
JLH1504	PY79 *amyE*::P_*hs*_*-rsiV*^S169W, Y261A^ *(spec)* Δ*sigVrsiV*::*kan*	
JLH1412	PY79 *pyrD*::P_*sigV*_*-lacZ* (*cat*) *rsiV*^*S169W*, *P259A*, *Y261A*^	
JLH1413	PY79 *pyrD*::P_*sigV*_*-lacZ* (*cat*) *rsiV*^*S169W*^	
JLH1380	PY79 *pyrD*::P_*sigV*_*-lacZ* (*cat*) *rsiV*^*P259A*^	
JLH1510	PY79 *pyrD*::P_*sigV*_*-lacZ* (*cat*) *rsiV*^*Y261A*^	
JLH1372	PY79 *pyrD*::P_*sigV*_*-lacZ* (*cat*) *rsiV* ^*P259A*, *Y261A*^	
JLH1507	PY79 *pyrD*::P_*sigV*_*-lacZ* (*cat*) *rsiV*^*S169W*, *Y261A*^	
JLH1362	PY79 *pyrD*::P_*sigV*_*-lacZ* (*cat*) *rsiV*^*H137A K139A*^	
JLH1536	PY79 *pyrD*::P_*sigV*_*-lacZ* (*cat*) *rsiV*^*S169W*, *P259A*^	
JLH1080	PY79 *amyE*::P_hs_-rsiV^T270A^ (spec) *pyrD*::P_*sigV*_*-lacZ (cat)*	
JLH1081	PY79 *amyE*::P_hs_-rsiV^T270A^ (spec) Δ*sigVrsiV;*:*kan*	
JLH1101	PY79 *amyE*::P_hs_-rsiV^S131P^ (spec) *pyrD*::P_*sigV*_*-lacZ (cat)*	
JLH1102	PY79 *amyE*::P_hs_-rsiV^S131P^ (spec) Δ*sigVrsiV*::*kan*	

^a^ This study, unless otherwise noted.

Site-directed mutants of *rsiV* were constructed using the QuickChange site-directed mutagenesis kit (Agilent Technologies) and Isothermal Assembly (Gibson *et al*., 2009). The sequences of the oligonucleotide primers used are listed in S2 Table. The IPTG-inducible hyper-spank (P_*hs*_) promoter was placed upstream of *rsiV* by assembling two *rsiV* PCR products into SphI digested pDR111 [78]. The two *rsiV* PCR products for the *rsiV*^*S169W*^ mutant were PCR amplified with CDEP1859/CDEP3092, and CDEP3093/CDEP1860. Each mutation was generated the same way using the appropriate primer pair with CDEP1850 and CDEP1860: S169W (CDEP3092/CDEP3093), P259A (CDEP3108/3109), Y261A (CDEP3110/CDEP3111), P259A and Y261A (CDEP3098/CDEP3099). S169W P259A Y261A, S169W P259A and S169W, Y261A were made using *rsiVS169W* (pJH375) as template and primers for P259A Y261A, P259A, or Y261A. The resulting plasmids: S169W (pJH375) P259A (pJH436), Y261A (pJH397), P259A Y261A (pJH398), S169W P259A (pJH405), S169W Y261A (pJH439), S169W P259A Y261A (pJH386) were transformed into CDE1563 *sigVrsiV*::*kan*, resulting in P_*hs*_-*rsiV*^*S169W*^ *sigVrsiV*::*kan* (JLH1271), P_*hs*_-*rsiV*^*P259A*^ *sigVrsiV*::*kan* (JLH1481), P_*hs*_-*rsiV*^*Y261A*^ *sigVrsiV*::*kan* (JLH1342), P_*hs*_-*rsiV*^*P259A Y261A*^ *sigVrsiV*::*kan* (JLH1343), P_*hs*_-*rsiV*^*S169W P259A*^ *sigVrsiV*::*kan* (JLH1326), P_*hs*_-*rsiV*^*S169W Y261A*^ *sigVrsiV*::*kan* (JLH1504), and P_*hs*_-*rsiV*^*S169W P259A Y261A*^ *sigVrsiV*::*kan* (JLH1312).

The site directed mutants were introduced onto the chromosome of PY79 by homologous recombination using the temperature sensitive plasmid pMAD (Arnaud *et al*., 2004). PCR products for *rsiV*^*S169W*^ were amplified with *rsiV* + 1 kb upstream using CDEP1892/CDEP3092 and *rsiV* + 1 kb downstream using CDEP1893/CDEP3093. The resulting PCR products were moved into SmaI digested pMAD using isothermal assembly. Each mutation was generated using the appropriate primer pair with CDEP1892/CDEP1893 or CDEP1892/CDEP3136 (adds 1.5 kb downstream *rsiV* to increase recombination efficiency): S169W (CDEP3092/CDEP3093), P259A (CDEP3108/3109), Y261A (CDEP3110/CDEP3111), P259A and Y261A (CDEP3098/CDEP3099). The triple and double mutants (S169W P259A Y261A, S169W P259A, and S169W, Y261A) were made using *rsiV*^*S169W*^ (JLH1413) as template with primers for P259A Y261A, P259A, or Y261A respectively. Each plasmid (S169W (pJH381) P259A (pJH403), Y261A (pJH438), P259A Y261A (pJH404), S169W P259A (pJH405), S169W Y261A (pJH437), and S169W P259A Y261A (pJH406)) was transformed into CDE1546 *P*_*sigV*_*-lacZ (cat)* resulting in *rsiV*^*S169W*^ *P*_*sigV*_*-lacZ* (JLH1413), *rsiV*^*P259A*^ *P*_*sigV*_*-lacZ* (JLH1380), *rsiV*^*Y261A*^ *P*_*sigV*_*-lacZ* (JLH1510), *rsiV*^*P259A Y261A*^ *P*_*sigV*_*-lacZ* (JLH1372), and *rsiV*^*S169W Y261A*^ *P*_*sigV*_*-lacZ* (JLH1507), and *rsiV*^*S169W P259A Y261A*^ *P*_*sigV*_*-lacZ* (JLH1412).

To generate constructs for purification the extracellular domain of RsiV *rsiV*^*59-285*^ was PCR amplified using primers CDEP1139 and CDEP952 and the already constructed plasmids as template: S169W (pJH375) P259A (pJH436), Y261A (pJH397), P259A Y261A (pJH398), S169W P259A Y261A (pJH386). This product was PCR amplified again with CDEP1140 and CDEP952 to add *2x-flag* and cloned into pEntrD-TOPO, resulting in plasmids: S169W (pJH421) P259A (pJH426), Y261A (pJH424), P259A Y261A (pJH425), S169W P259A (pJH446), S169W Y261A (pJH448), and S169W P259A Y261A (pJH423). To construct recombinant 6x-His-2x-Flag-RsiV^(59–285)^ + site directed mutant, *2x-flag-rsiV*^*(59–285)*^ was moved from each pEntrD-TOPO vector into the T7-inducible 6x-His destination vector pDEST17 (Invitrogen) using LR Clonase II. The following plasmids were moved into BL21λDE3 cells for expression: S169W (pJH427), P259A (pJH450), Y261A (pJH430), P259A Y261A (pJH431), S169W P259A (pJH447), and S169W P259A Y261A (pJH429).

### Medium supplements

Antibiotics were used at the following concentrations: chloramphenicol, 5 μg/ml; erythromycin plus lincomycin, 1 μg/ml and 25 μg/ml; kanamycin, 5 μg/ml; spectinomycin, 100 μg/ml; tetracycline, 10 μg/ml; ampicillin 100 μg/ml. The β-galactosidase chromogenic indicator 5-bromo-4-chloro-3-indolyl β-D-galactopyranoside (X-Gal) was used at a concentration 100 μg/ml. Isopropyl β-D-1-thiogalactopyranoside (IPTG) was used at a final concentration of 1 mM unless otherwise noted.

### Immunoblot analysis

Strains were grown for 16 hours in LB at 37°C. The cells were subcultured 1:100 in LB+1 mM IPTG at 37°C and grown to an OD_600_ of 0.8–1. The cells were pelleted by centrifugation and resuspended in 100 μl of 2X Laemmli sample buffer and lysed by repeated sonication. Samples were electrophoresed on a 15% SDS polyacrylamide gel (BioRad). The proteins were then blotted onto nitrocellulose. The nitrocellulose was blocked with 5% milk for 30 minutes. The proteins were detected by incubating with a 1:10,000 dilution of anti-RsiV^59–285^ antibodies (Hastie *et al*., 2013) or 1:15,000 dilution of anti-σ^A^ antibodies followed by 3 washes and incubation in a 1:10,000 dilution of goat anti-rabbit IgG (H+L) IRDye 800CW (Li-Cor) and imaged on an Odyssey CLx (Li-Cor). Quantification of band intensities was performed using Image Studio software (Li-Cor).

### β-galactosidase activity assays

Cultures were grown overnight in LB broth at 30°C and 20 μl were spotted onto LB agar + 0, 2, 5, 10 or 20 μg/ml lysozyme. Plates were incubated at 37°C for 6 hours. Cells were harvested and resuspended in 500 μl of Z buffer (60 mM Na_2_HPO_4_, 40 mM NaH_2_PO_4_, 10 mM KCl, 1 mM MgSO_4_, 50 mM β-mercaptoethanol pH 7.0). Cells were transferred to a 96 well plate and optical density (OD_600_) determined. Cells were permeabilized by mixing with chloroform and 2% sarkosyl [5,79]. Permeabilized cells (100 μl) were mixed with 10 mg/ml ortho-Nitrophenyl-β-galactoside (ONPG, RPI, 50 μl) and OD_405_ was measured over time. β-galactosidase activity units (μmol of ONP formed min^−1^) X 10^3^/(OD_600_ X ml of cell suspension) were calculated as previously described [80]. Experiments were performed in triplicate with the mean and standard deviation shown.

### Quantitative Reverse Transcriptase PCR

For each sample, a single colony of the appropriate *B*. *subtilis* strain was inoculated in LB medium and grown overnight. The overnight cultures were subcultured into LB or LB + 1 mM IPTG and grown to -OD_600_ of 0.8 at which point lysozyme when necessary was added to a final concentration of 1.25 μg/ml and grown for 1 hour. RNA was extracted using the RNeasy RNA Isolation kit (Qiagen). Contaminating DNA was removed using the Turbo DNA-free kit protocol (Ambion). Samples were tested for DNA contamination by PCR amplification (Thermo Taq polymerase, NEB) using primers CDEP1017 and CDEP1018 (S3 Table).

To generate cDNA from RNA samples, we used Superscript II (Invitrogen) according to manufacturer’s protocols. The resulting reverse transcription reactions were diluted 1:5 in DEPC-treated water. For each quantitative RT PCR reaction, 5 μl of sample was added to 10 μl of power Sybr green master mix (Applied Biosystems) and 5 μl gene-specific primers (2 x 2.5 μM). The list of primers used to quantitate cDNA levels of different samples is provided in S3 Table. Experiments were performed on three biologically independent replicates. Data were normalized to RNA levels of the housekeeping gene *rpoB*.

### Expression of recombinant proteins in *E*. *coli* and purification

Samples were prepared the same as previously described [12]. Briefly, overnight cultures of *E*. *coli* BL21λDE3 containing WT RsiV pKBW201 (pDEST17-*6xhis-2xflag*-*rsiV*^59–285^) or mutant RsiV S169W (pJH427), P259A (pJH450), Y261A (pJH430), P259A Y261A (pJH431), S169W P259A (pJH447), and S169W P259A Y261A (pJH429) were grown at 30°C in LB + ampicillin. The cell cultures were diluted 1:100 into 500 ml of LB + ampicillin in 2 L baffled flasks and incubated at 30°C to an OD_600_ of 0.5–0.6. IPTG was added to a final concentration of 1 mM to induce protein production and the cultures incubated for an additional 4 hours. Cells were chilled on ice and collected by centrifugation at 5000xg. Cell pellets were stored at −80°C until time for purification. Cell pellets were thawed on ice and resuspended in 5 ml lysis buffer (50 mM NaH_2_PO_4_, 250 mM NaCl, 10 mM imidazole, pH 8.0) per 500 ml of initial culture volume. Cells were lysed by passaging through a Microfluidics LV1 high shear microfluidizer (Newton, MA) twice. Lysate was centrifuged at 15,000x*g*, for 30 minutes at 4°C to pellet cellular debris. Cleared lysate was applied to a nickel affinity column to bind 6xHis-tagged protein (Qiagen). The column was washed with 10 column volumes of wash buffer (50 mM NaH_2_PO_4_, 250 mM NaCl, 20 mM imidazole, pH 8.0). Protein was eluted with elution buffer (50 mM NaH_2_PO_4_, 250 mM NaCl, 250 mM imidazole, pH 8.0) and collected in 0.5 ml fractions. Samples from each fraction were analyzed by SDS-PAGE and elution fractions containing the desired protein were combined. Combined fractions were then dialyzed into lysis buffer to remove the excess imidazole.

### Crystallization of RsiV-lysozyme

RsiV and HEW-lysozyme mixed in a 1:3 molar ratio were allowed to incubate for 60 minutes, at 4°C, and run through a Superdex-75 size exclusion column (GE). Fractions representing the complex confirmed by running a SDS-PAGE gel stained with Coomassie Blue were pooled and concentrated using a Amicon 30kDa filter to 8.9 mg/ml. Crystallization drops with commercially available screens using a TTP LabTech Mosquito robot were set-up via the hanging-drop vapor diffusion method at 18°C with the drop containing 0.4 μl each of protein and crystallization solutions. Crystals were obtained in a variety of PEG containing conditions within 14 days. In order to perform experimental phasing selenomethionine labeled RsiV protein was prepared. For production for selenomethionine labeled protein, cells were grown at 35°C overnight in M9 minimal media supplemented with 100 μg/ml ampicillin. The following morning cells were subcultured 1:50 into 1L flasks of M9 minimal media with ampicillin and incubated at 35°C. When the cultures reached O.D.600 of 0.5, 100 mg/L of lysine, phenylalanine, threonine, and 50 mg/L of isoleucine, leucine, and valine were added to inhibit methionine synthesis as described [81]. Selenomethionine was added to the media at 60 mg/L [81] and the temperature shifted to 28°C. 15 minutes after addition of amino acid cocktail, IPTG was added to a final concentration of 1mM to induce protein expression. Purification of the selenomethionine labeled protein proceeded as described for unlabeled RsiV. Selenomethionine labeled RsiV was purified as described above complexed with lysozyme and crystallization plates set up similar to the wild type protein. Crystals obtained in 0.2 M sodium nitrate, 20% w/v PEG 3350 were harvested for diffraction data collection.

### Data collection and structure determination

Crystals were flash-cooled in liquid nitrogen and shipped to the 4.2.2 synchotron beamline at the Advanced Light Source (Berkeley, CA, USA) for remote data collection. The data were processed using XDS [82]. Pointless [83,84] and Aimless [84–89] from the CCP4 [90] software suite were used for conversion of intensities to structure factors and scaling. Structure solution, initial model building and refinements were performed using AutoSol [91], AutoBuild [92] and Phenix.refine [93] from the Phenix suite [93]. Model building was performed in Coot [94] and all structural figures were generated using PyMOL [95]. All crystallography software used were configured and deployed using SBGrid [96]. The surface interface of RsiV and lysozyme was determined using Protein interfaces, surfaces and assemblies' service PISA at the European Bioinformatics Institute (http://www.ebi.ac.uk/pdbe/prot_int/pistart.html) [29].

### Isothermal Titration Calorimetry

ITC analysis was performed as previously described [12]. Briefly, 6xHis-2xFlag-RsiV^59–285^ and mutants were purified as described above and buffer matched with HEW lysozyme (≥98% pure, Sigma Aldrich) by dialysis into 50 mM Na_2_HPO_4_, 200 mM NaCl, and pH 7.0 for 24 h at 4°C. Final protein concentrations as determined by absorbance at OD_280_ were adjusted to 6xHis-2xFlag-RsiV^59–285^ (0.01 mM) and HEW lysozyme (0.1 mM) with filtered dialysate. The protein samples were degassed and ITC measurements recorded using a MicroCal VP-ITC System (GE Healthcare) with HEW lysozyme as the injected sample and 6_X_His-2_X_Flag-RsiV^59–285^ as the cell sample. 21 injections of HEW lysozyme were used, with 180 seconds spacing between events. The chamber was kept under constant stirring at 350 rpm and all experiments were performed at 25°C. The binding reaction reached saturation during the experiment and control experiments where HEW lysozyme was injected into buffer showed that the heats of dilution were constant across all injections. The constant heat of dilution, as determined by the average of the last 3–5 injections, was subtracted and the data are analyzed using the single site binding model provided in the ITC analysis package. The values for affinity were averaged from three separate runs from two different protein preps.

### Circular dichroism

Protein from 6xHis-2xFlag-RsiV^59–285^ and 6xHis-2xFlag-RsiV^59-285, S169W, P259A, Y261A^ was purified as described above. Samples were further purified on a BioRad DuoFlow FPLC with a Superdex 75 (GE) size exclusion column. Fractions were pooled and the concentration was determined by OD_280_ with an extinction coefficient calculated for each mutant as described [97]. Samples were diluted to 20 μM and analyzed using a 1mm cuvette in a Jasco J-815 CD spectropolarimeter.

### Lysozyme inhibition assay

Increasing concentrations of purified RsiV (prepared as described above) were mixed with lysozyme (20 μg/ml) for 15 minutes. The purified RsiV + lysozyme (100 μl) mixture was combined with *M*. *lysodekticus* (100 μl, OD_450_ = 0.9, Sigma) The OD_450_ was measured every minute for 30 minutes to monitor *M*. *lysodekticus* degradation. Lysozyme activity was calculated based on the equation as described by Sigma [41]. Briefly, units/ml enzyme = (ΔOD_450_)*(dilution factor)/ (0.001*0.05), Specific Activity = (units/ml enzyme) / (mg solid/ ml enzyme).

### Determination of minimum inhibitory concentration

The minimum inhibitor concentration (MIC) was determined by diluting overnight cultures 1:100 in 1:50 LB + 1 mM IPTG. The cells were inoculated into 250 μl of LB + 1 mM IPTG containing serial dilutions of hen egg white lysozyme ranging from 20 μg/ml to 1.875 μg/ml in a round bottom 96 well plate. The cells were grown for 16 hours at 37°C. Growth was defined as OD_600_ of greater than 0.05. All assays were performed using all strains listed in triplicate on the same day.

## Supporting Information

S1 Fig RsiV domain structure and homolog alignments. **A.** Cartoon of the domain structure of RsiV. The domains are labelled as described. The N-terminal region is located in the cytosol and contains the σ^V^ binding domain. TM refers to the predicted transmembrane domain. The * denotes the signal peptidase cleavage site. The DUF4163 and DUF3298 refer to domains of unknown function. **B.** Alignment of RsiV from *B*. *subtilis*, *C*. *difficile* and *E*. *faecalis*. The domains are labeled above the sequences. The dashed line denotes a likely extension of the DUF4163 domain. The signal peptidase recognition sequence is highlighted yellow. * denotes a fully conserved residue,: Strong group conservation,. Weak group conservation. **C.** A cartoon structure of the lysozyme binding domain of RsiV highlighting the DUF4163 (red) and DUF3298 (blue) domains.
(TIF)

S2 Fig Structural alignment of RsiV with orthologs from other organisms. **A.** Structure alignment of an uncharacterized protein from *Fervidobacterium nodosum* Rt17-B1 (PDB:3CYG) (yellow) and the extracellular domain of RsiV (red). **B.** Structure alignment of an uncharacterized protein BF2082 from *Bacteroides fragilis* NCTC 9343 (PDB:3S5T) (yellow) and the extracellular domain of RsiV (red). **C.** Structure alignment of an uncharacterized protein from PA4972 from *Pseudomonas aeruginosa* PAO1 (PDB:4E72) (yellow) and the extracellular domain of RsiV (red). **D.** Structure alignment of Rv1980c (MPT64) from *Mycobacterium tuberculosis* (PDB:2HHI) (yellow) and the extracellular domain of RsiV (red). Structural Alignment was conducted in PyMOL, using the align function [95].
(TIF)

S3 Fig Amino acid alignment of RsiV with orthologs from other organisms. Amino acid sequences of the extracellular domain of RsiV and those of *Fervidobacterium nodosum* Rt17-B1 (PDB:3CYG), BF2082 from *Bacteroides fragilis* NCTC 9343 (PDB:3S5T), PA4972 from *Pseudomonas aeruginosa* PAO1 (PDB:4E72), and MPT64 from *Mycobacterium tuberculosis* (PDB:2HHI) were aligned using ClustalW. Residues in Red are those identified as critical to RsiV binding to lysozyme or equivalent in other orthologs. * denotes a fully conserved residue,: Strong group conservation,. Weak group conservation.
(TIF)

S4 Fig Rv1980c does not bind lysozyme. Samples from a lysozyme pull-down assay were separated on a 15% SDS-PAGE gel and stained with Coomassie brilliant blue. The pull down assay was performed as described in Materials and Methods. Recombinant 6xHis-Rv1980c was used in this experiment. Elution fractions show 6xHis-Rv1980c and HEW lysozyme do not elute from a Ni affinity column in the same fractions. Arrows indicate where 6xHis-Rv1980c and HEW Lysozyme migrate.
(TIF)

S5 Fig Regions of highest homology mapped on the structure of lysozyme. Space fill model of hen egg white lysozyme based on 1lyz [100]. The amino acid residues of lysozyme are colored according to degree of conservation with 400 other C-type lysozyme homologs using ClustalW [29]. The ClustalW homology was overlaid on the lysozyme structure using ConSurf [30,31]. The darker maroon color indicates higher conservation while white is neutral and the darker blues are the least conserved amino acid residues. The image on the right has been rotated 180° clockwise. Below is a bottom view of the active site and peptidoglycan biding pocket of lysozyme.
(TIF)

S6 Fig ITC of RsiV mutants. Representative run of ITC experiments conducted with combinations of the S169W, P259A, Y261A triple mutant and lysozyme. RsiV was loaded in the cell at a concentration of 0.01 mM and lysozyme was loaded in the syringe at 0.1 mM. **A**. RsiV^59-285^ (P259A, Y261A), **B.** RsiV^59-285^ (S169W), **C**. RsiV^59-285^ (P259A), **D**. RsiV^59-285^ (S169W, P259A), **E**. RsiV^59-285^ (Y261A), **F**. RsiV^59-285^ (S169W, Y261A).
(TIF)

S7 Fig Circular dichroism spectra of RsiV^S169W, P259A, Y261A^. Protein samples were purified and the concentration was determined by OD_280_. Samples were diluted to 20 μM and analyzed using a 1mm cuvette in a Jasco J-815 CD spectropolarimeter. Data shown is the average of two spectral scans.
(TIF)

S8 Fig RsiV mutants do not have altered levels in the absence of lysozyme. **A.** Immunoblot analysis of RsiV mutant protein levels when grown at 1mM. Overnigh*t B*. *subtilis* strains were subcultured 1:100 into media with 1mM IPTG* and grown to OD_600_ of 1. Cells were pelleted, resuspended in 100 μl sample buffer, lysed, and immunoblotted with anti-RsiV antibodies. **B.** Immunoblot analysis of RsiV mutant protein levels with matched expression levels. Strains JLH1481 (P259A) and JLH1504 (S169W, Y261A) levels were subcultured into LB + 0.1 mM IPTG while the remaining cultures were subcultured in LB + 1 mM IPTG. Cells were pelleted, resuspended in 100 μl sample buffer, lysed, and immunoblotted with anti-RsiV antibodies.
(TIF)

S9 Fig RsiV inhibition of lysozyme requires binding to lysozyme. Peptidoglycan from *M*. *lysodekticus* was combined with lysozyme (20 μg/ ml) and purified RsiV or RsiV mutants at a molar ratio of 0, 1, 2 or 4. The OD_450_ was monitored every minute for 30 minutes to determine lysozyme specific activity. **A.** WT RsiV^59-285^; **B**. RsiV^59-285^(P259A, Y261); **C.** RsiV^59-285^(P259A); **D.** RsiV^59-285^(Y261A); **E**. RsiV^59-285^(S169W, P259A, Y261A); **F.** RsiV^59-285^(S169W); **G.** RsiV^59-285^(S169W, P259A); and **H.** RsiV^59-285^(S169W, Y261A).
(TIF)

S10 Fig RsiV inhibition of lysozyme requires binding to lysozyme. Immunoblot analysis of RsiV protein levels. Overnigh*t B*. *subtilis* strains were subcultured 1:100 into LB WT (PY79) and *ΔsigVrsiV* (CDE1563) or *P*_*hs*_*-rsiV* (JLH402) LB + 1mM IPTG and grown to OD_600_ of 1. Cells were pelleted, resuspended in 100 μl sample buffer, lysed. 2-fold serial dilutions of purified His6-3xFlag-RsiV were loaded were prepared and loaded on an SDS-PAGE gel. Proteins were separated by SDS-PAGE and immunoblotted with anti-RsiV antibodies.
(TIF)

S1 Methods Co-purification experiments. (PDF)

S1 Table BLASTP results of RsiV homologs. (PDF)

S2 Table Relative expression in response to lysozyme exposure or IPTG induction. (PDF)

S3 Table Oligonucleotides. (PDF)
