# Supplementary figures and images for: The Anti-sigma Factor RsiV Is a Bacterial Receptor for Lysozyme: Co-crystal Structure Determination and Demonstration That Binding of Lysozyme to RsiV Is Required for σV Activation
Source: PLoS Genet. 2016 Sep 7;12(9):e1006287. doi: 10.1371/journal.pgen.1006287 (PMC5014341; doi:10.1371/journal.pgen.1006287)

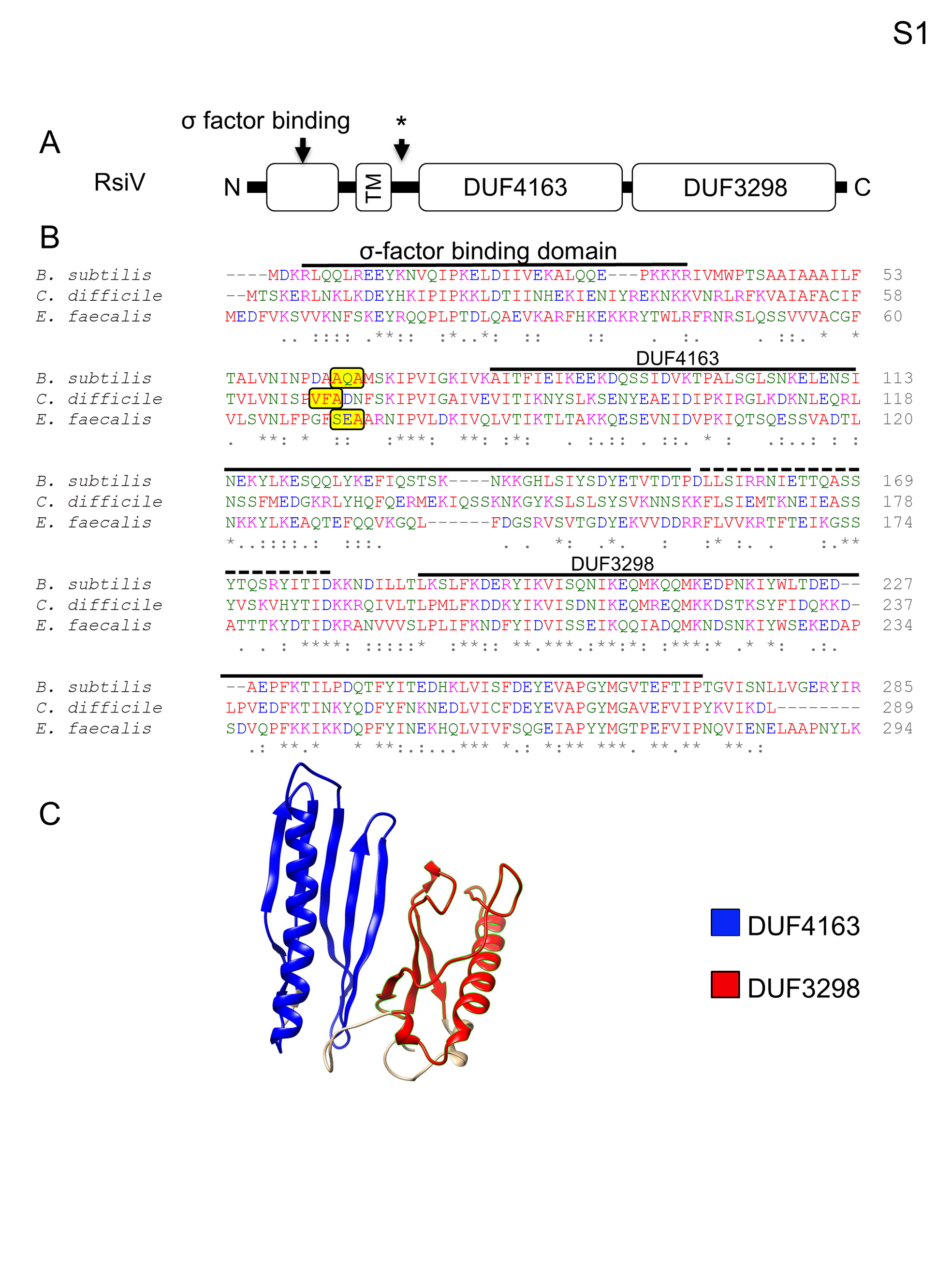

Supplement: S1 Fig — A. Cartoon of the domain structure of RsiV. The domains are labelled as described. The N-terminal region is located in the cytosol and contains the σV binding domain. TM refers to the predicted transmembrane domain. The * denotes the signal peptidase cleavage site. The DUF4163 and DUF3298 refer to domains of unknown function. B. Alignment of RsiV from B. subtilis, C. difficile and E. faecalis. The domains are labeled above the sequences. The dashed line denotes a likely extension of the DUF4163 domain. The signal peptidase recognition sequence is highlighted yellow. * denotes a fully conserved residue,: Strong group conservation,. Weak group conservation. C. A cartoon structure of the lysozyme binding domain of RsiV highlighting the DUF4163 (red) and DUF3298 (blue) domains. (TIF) [file pgen.1006287.s001.tif]

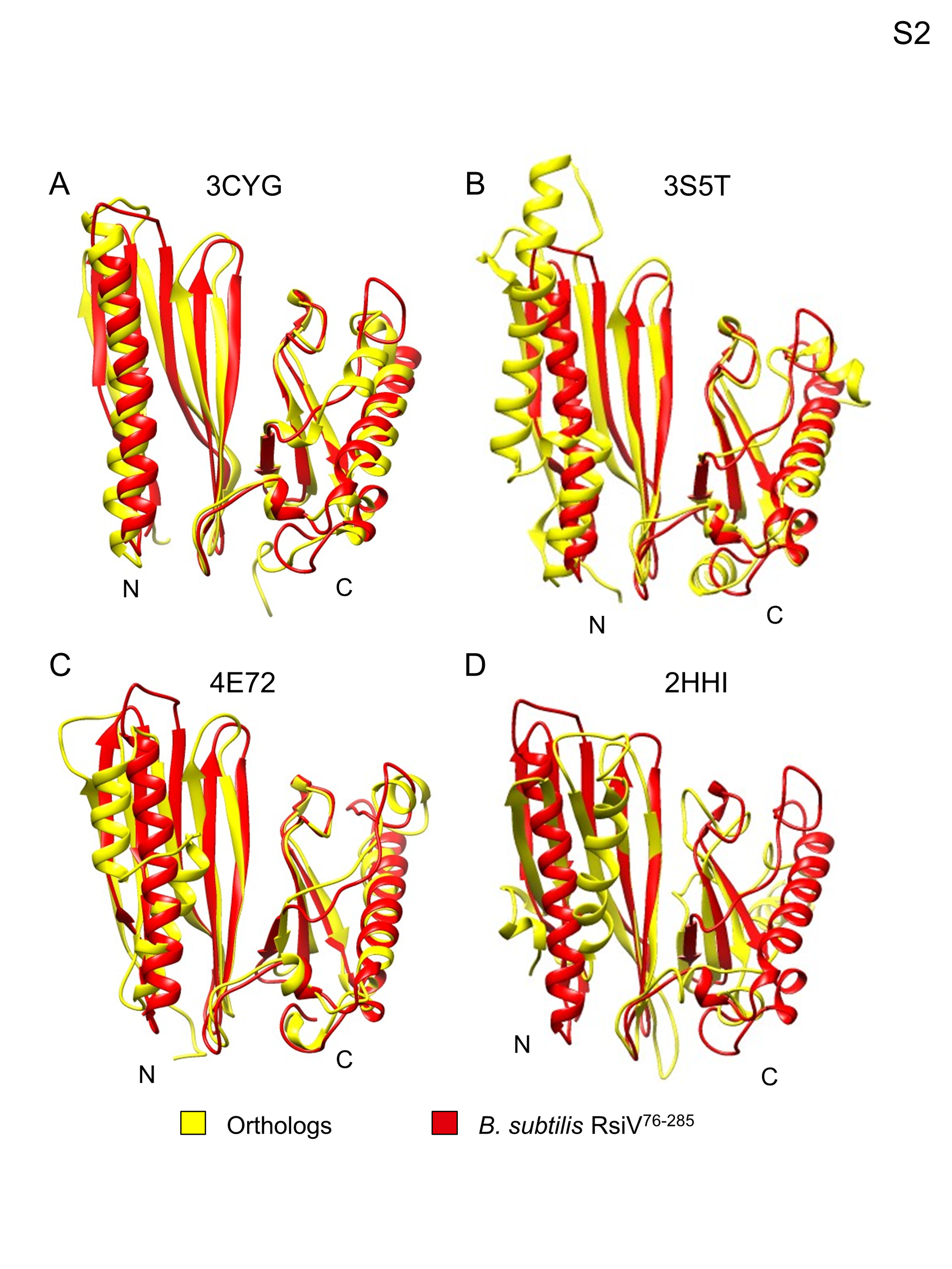

Supplement: S2 Fig — A. Structure alignment of an uncharacterized protein from Fervidobacterium nodosum Rt17-B1 (PDB:3CYG) (yellow) and the extracellular domain of RsiV (red). B. Structure alignment of an uncharacterized protein BF2082 from Bacteroides fragilis NCTC 9343 (PDB:3S5T) (yellow) and the extracellular domain of RsiV (red). C. Structure alignment of an uncharacterized protein from PA4972 from Pseudomonas aeruginosa PAO1 (PDB:4E72) (yellow) and the extracellular domain of RsiV (red). D. Structure alignment of Rv1980c (MPT64) from Mycobacterium tuberculosis (PDB:2HHI) (yellow) and the extracellular domain of RsiV (red). Structural Alignment was conducted in PyMOL, using the align function [95]. (TIF) [file pgen.1006287.s002.tif]

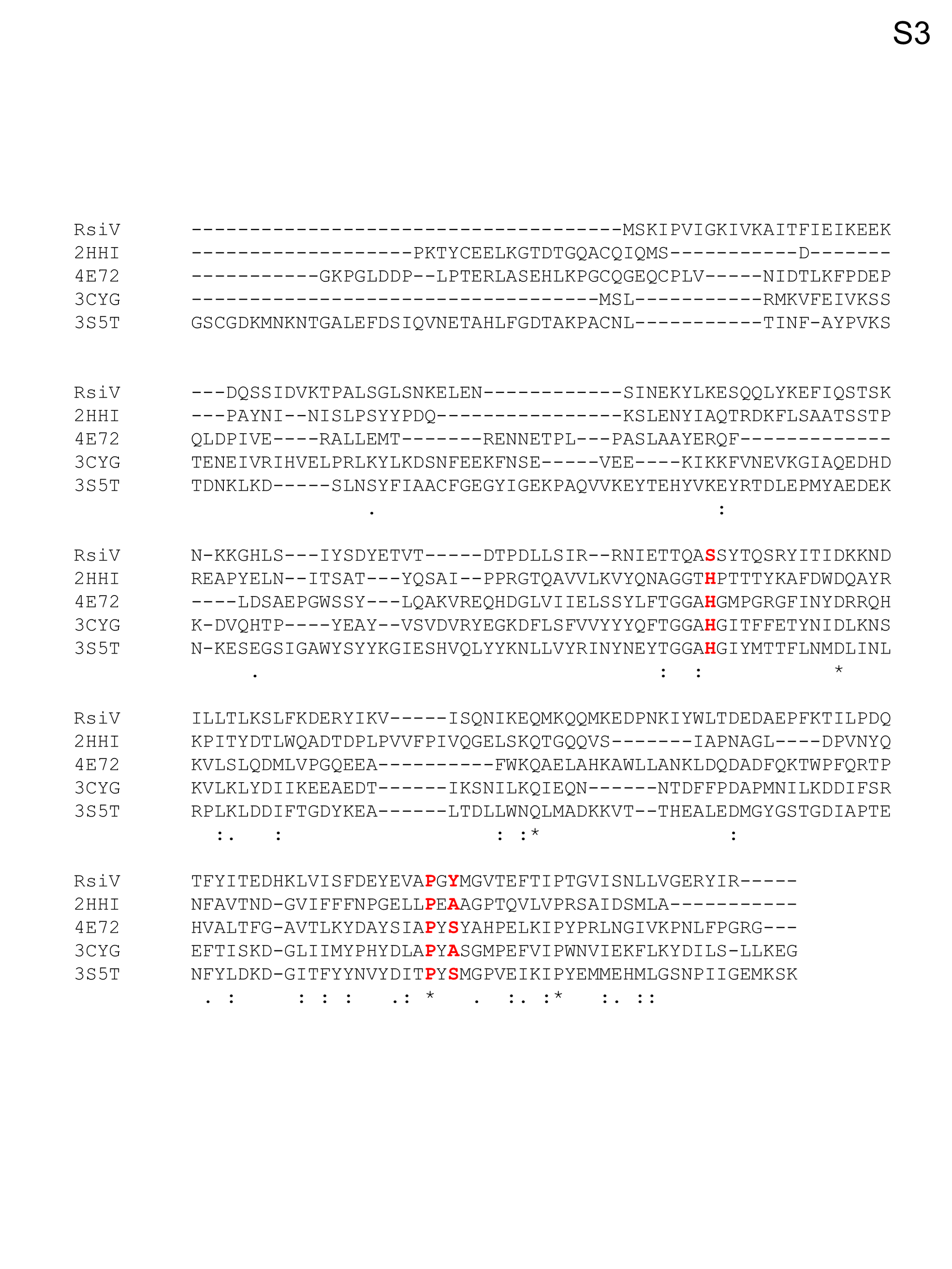

Supplement: S3 Fig — Amino acid sequences of the extracellular domain of RsiV and those of Fervidobacterium nodosum Rt17-B1 (PDB:3CYG), BF2082 from Bacteroides fragilis NCTC 9343 (PDB:3S5T), PA4972 from Pseudomonas aeruginosa PAO1 (PDB:4E72), and MPT64 from Mycobacterium tuberculosis (PDB:2HHI) were aligned using ClustalW. Residues in Red are those identified as critical to RsiV binding to lysozyme or equivalent in other orthologs. * denotes a fully conserved residue,: Strong group conservation,. Weak group conservation. (TIF) [file pgen.1006287.s003.tif]

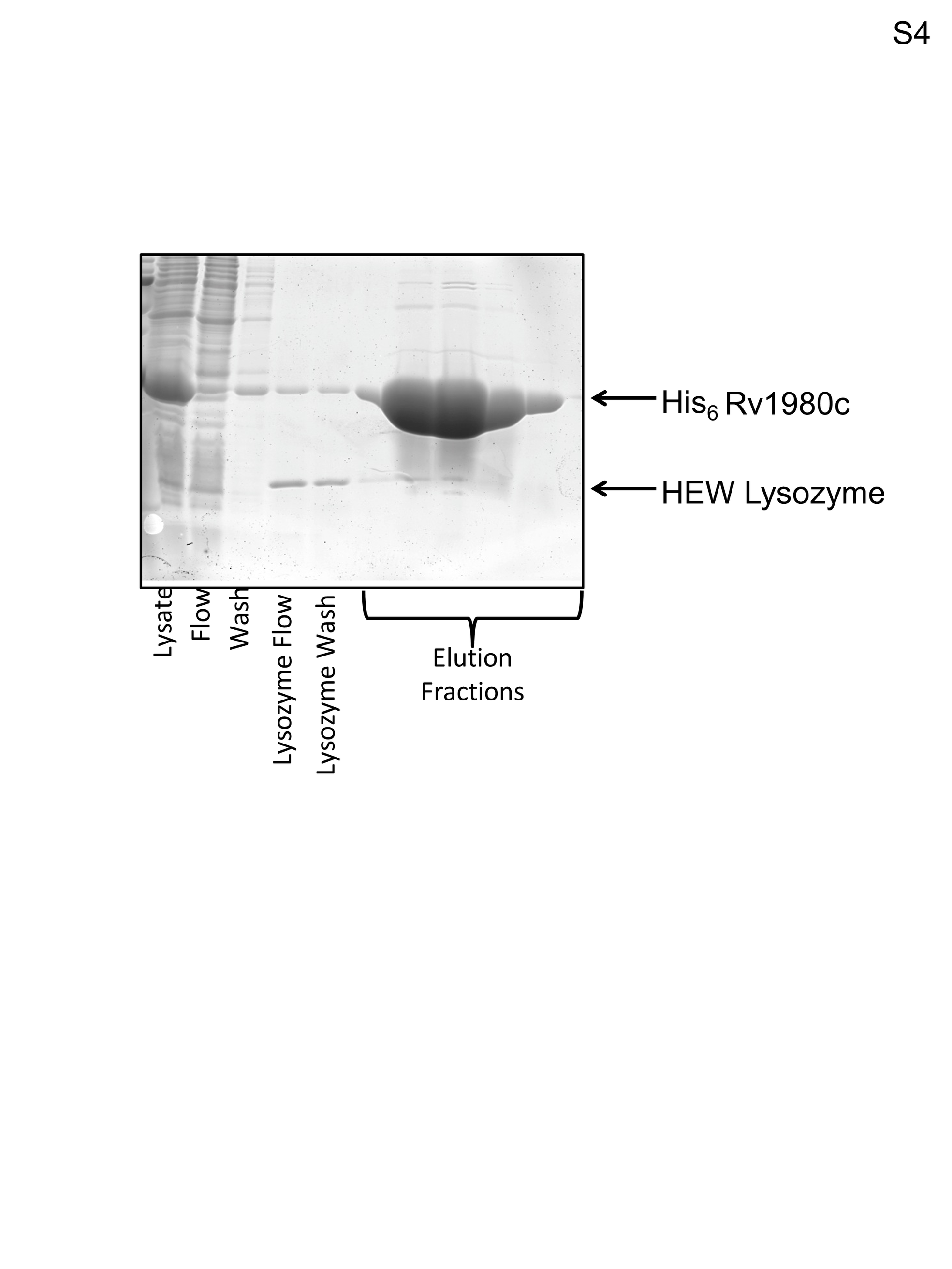

Supplement: S4 Fig — Samples from a lysozyme pull-down assay were separated on a 15% SDS-PAGE gel and stained with Coomassie brilliant blue. The pull down assay was performed as described in Materials and Methods. Recombinant 6xHis-Rv1980c was used in this experiment. Elution fractions show 6xHis-Rv1980c and HEW lysozyme do not elute from a Ni affinity column in the same fractions. Arrows indicate where 6xHis-Rv1980c and HEW Lysozyme migrate. (TIF) [file pgen.1006287.s004.tif]

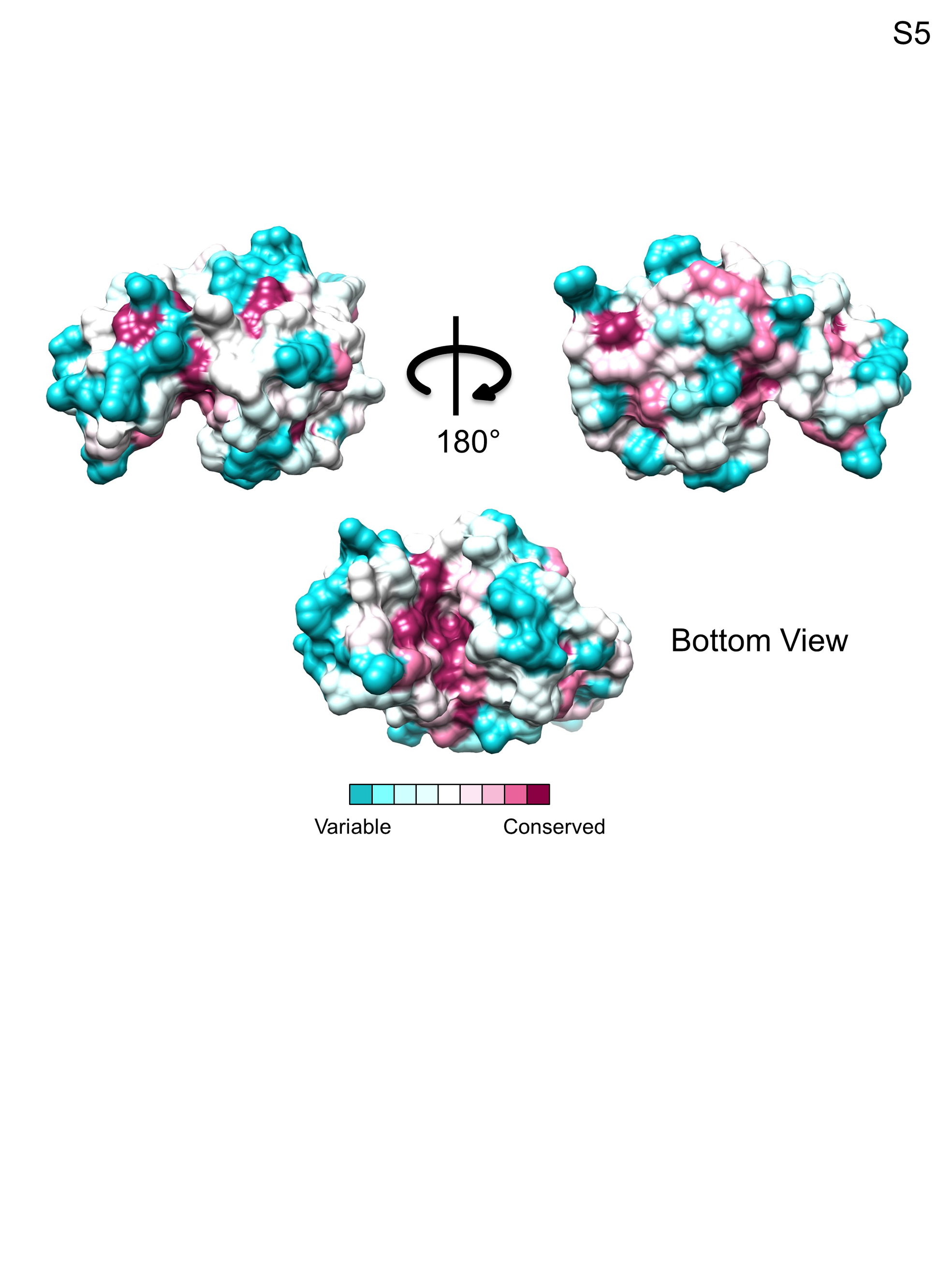

Supplement: S5 Fig — Space fill model of hen egg white lysozyme based on 1lyz [100]. The amino acid residues of lysozyme are colored according to degree of conservation with 400 other C-type lysozyme homologs using ClustalW [29]. The ClustalW homology was overlaid on the lysozyme structure using ConSurf [30,31]. The darker maroon color indicates higher conservation while white is neutral and the darker blues are the least conserved amino acid residues. The image on the right has been rotated 180° clockwise. Below is a bottom view of the active site and peptidoglycan biding pocket of lysozyme. (TIF) [file pgen.1006287.s005.tif]

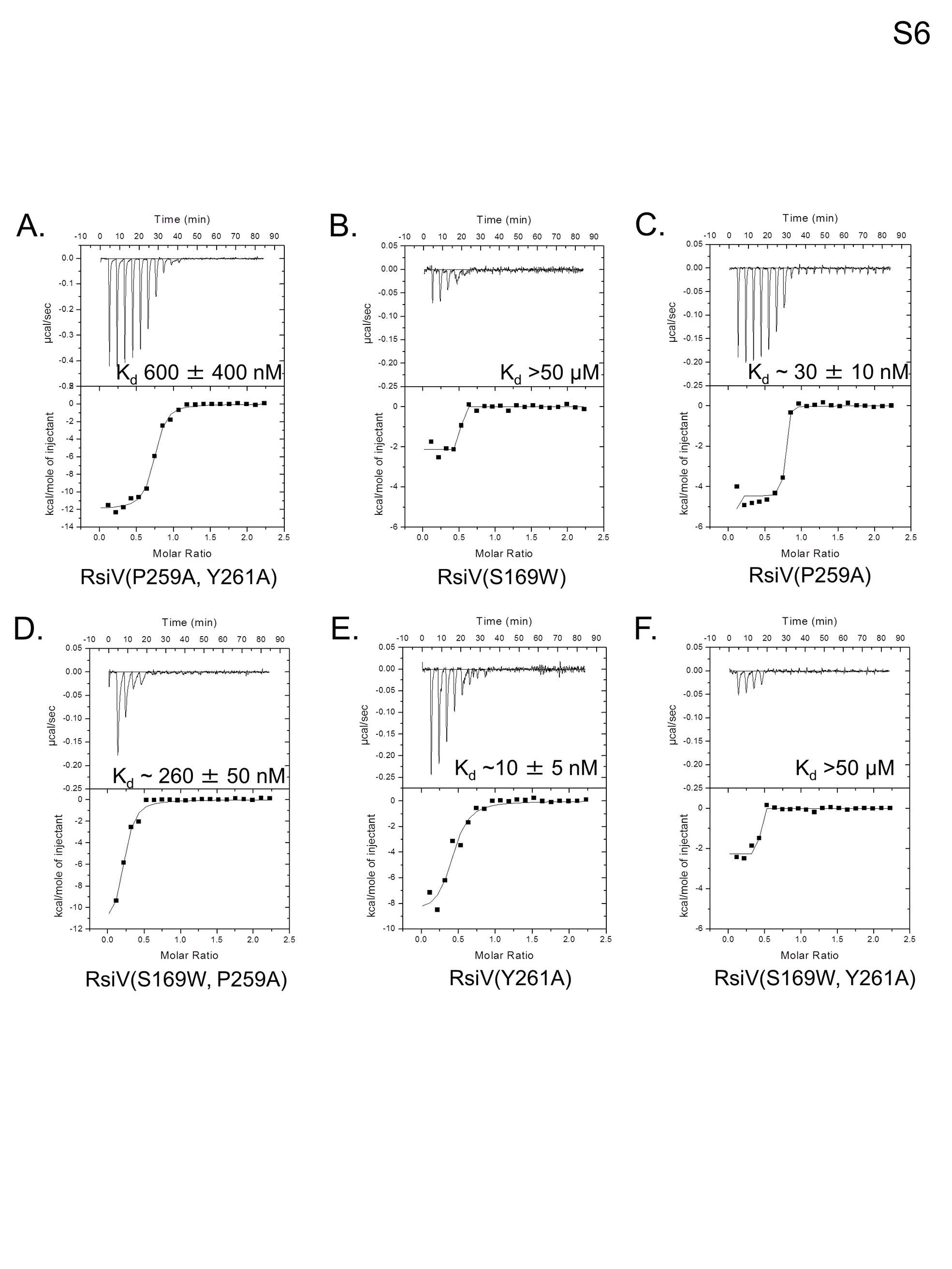

Supplement: S6 Fig — Representative run of ITC experiments conducted with combinations of the S169W, P259A, Y261A triple mutant and lysozyme. RsiV was loaded in the cell at a concentration of 0.01 mM and lysozyme was loaded in the syringe at 0.1 mM. A. RsiV59-285 (P259A, Y261A), B. RsiV59-285 (S169W), C. RsiV59-285 (P259A), D. RsiV59-285 (S169W, P259A), E. RsiV59-285 (Y261A), F. RsiV59-285 (S169W, Y261A). (TIF) [file pgen.1006287.s006.tif]

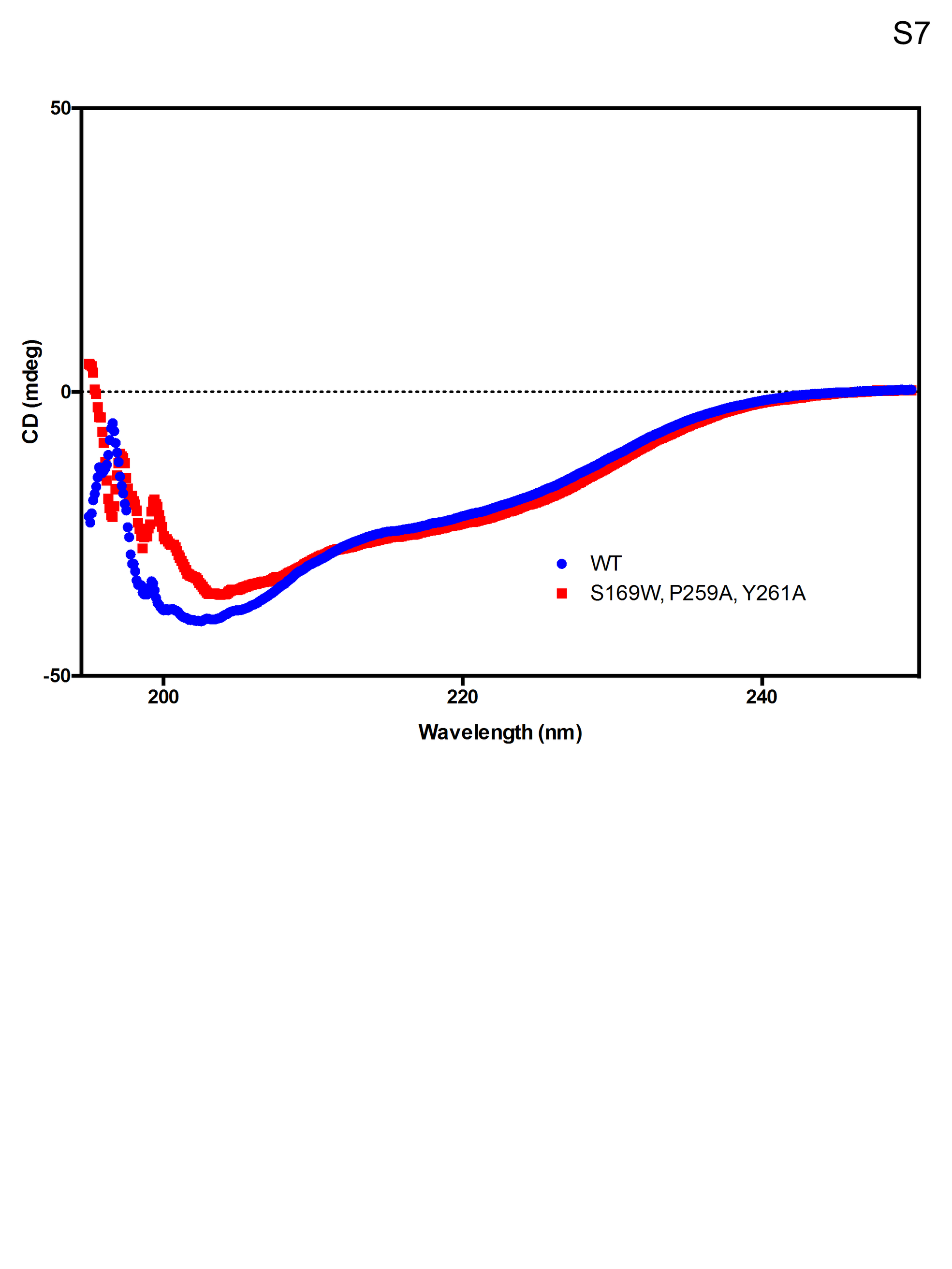

Supplement: S7 Fig — Protein samples were purified and the concentration was determined by OD280. Samples were diluted to 20 μM and analyzed using a 1mm cuvette in a Jasco J-815 CD spectropolarimeter. Data shown is the average of two spectral scans. (TIF) [file pgen.1006287.s007.tif]

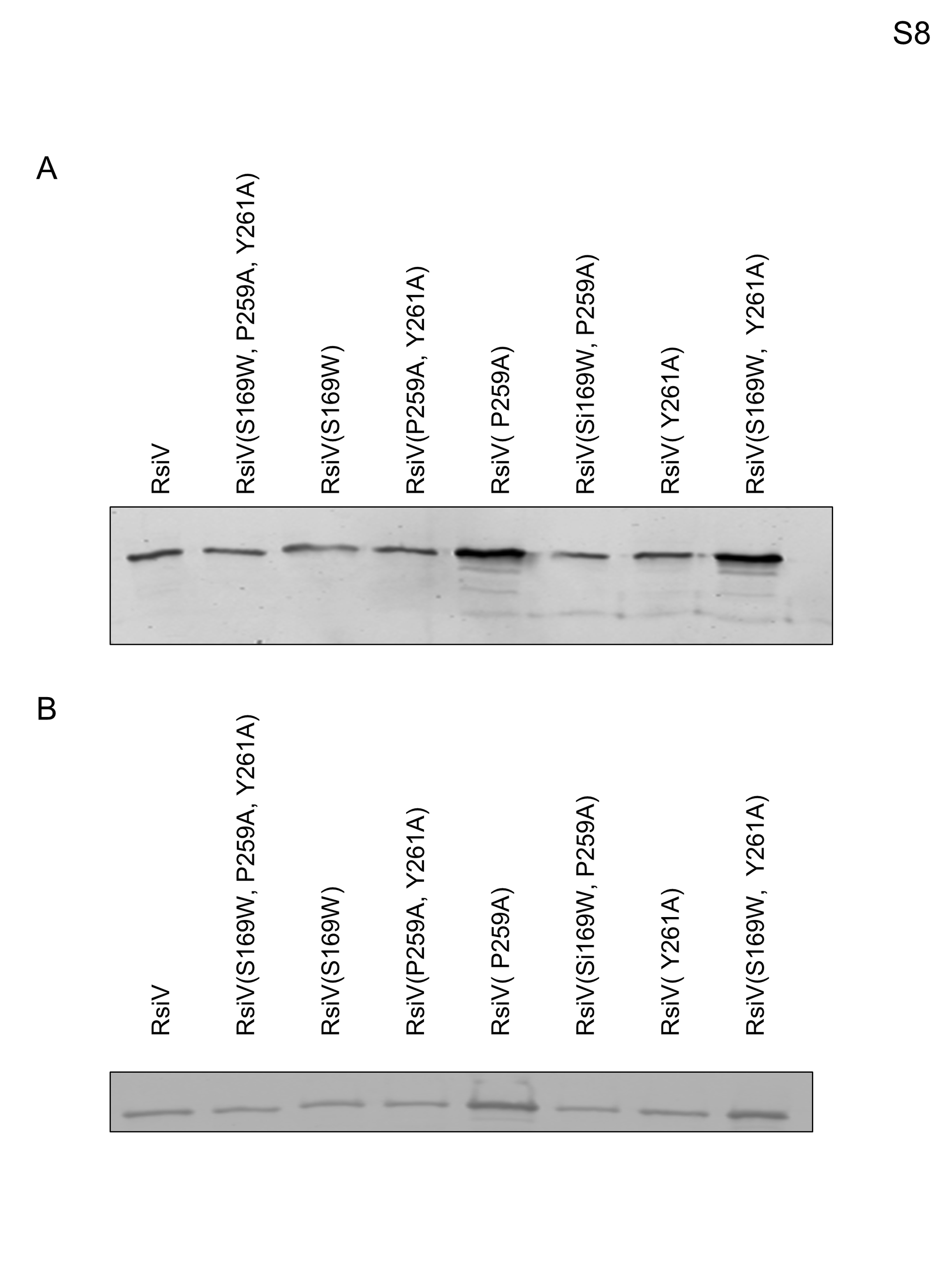

Supplement: S8 Fig — A. Immunoblot analysis of RsiV mutant protein levels when grown at 1mM. Overnight B. subtilis strains were subcultured 1:100 into media with 1mM IPTG* and grown to OD600 of 1. Cells were pelleted, resuspended in 100 μl sample buffer, lysed, and immunoblotted with anti-RsiV antibodies. B. Immunoblot analysis of RsiV mutant protein levels with matched expression levels. Strains JLH1481 (P259A) and JLH1504 (S169W, Y261A) levels were subcultured into LB + 0.1 mM IPTG while the remaining cultures were subcultured in LB + 1 mM IPTG. Cells were pelleted, resuspended in 100 μl sample buffer, lysed, and immunoblotted with anti-RsiV antibodies. (TIF) [file pgen.1006287.s008.tif]

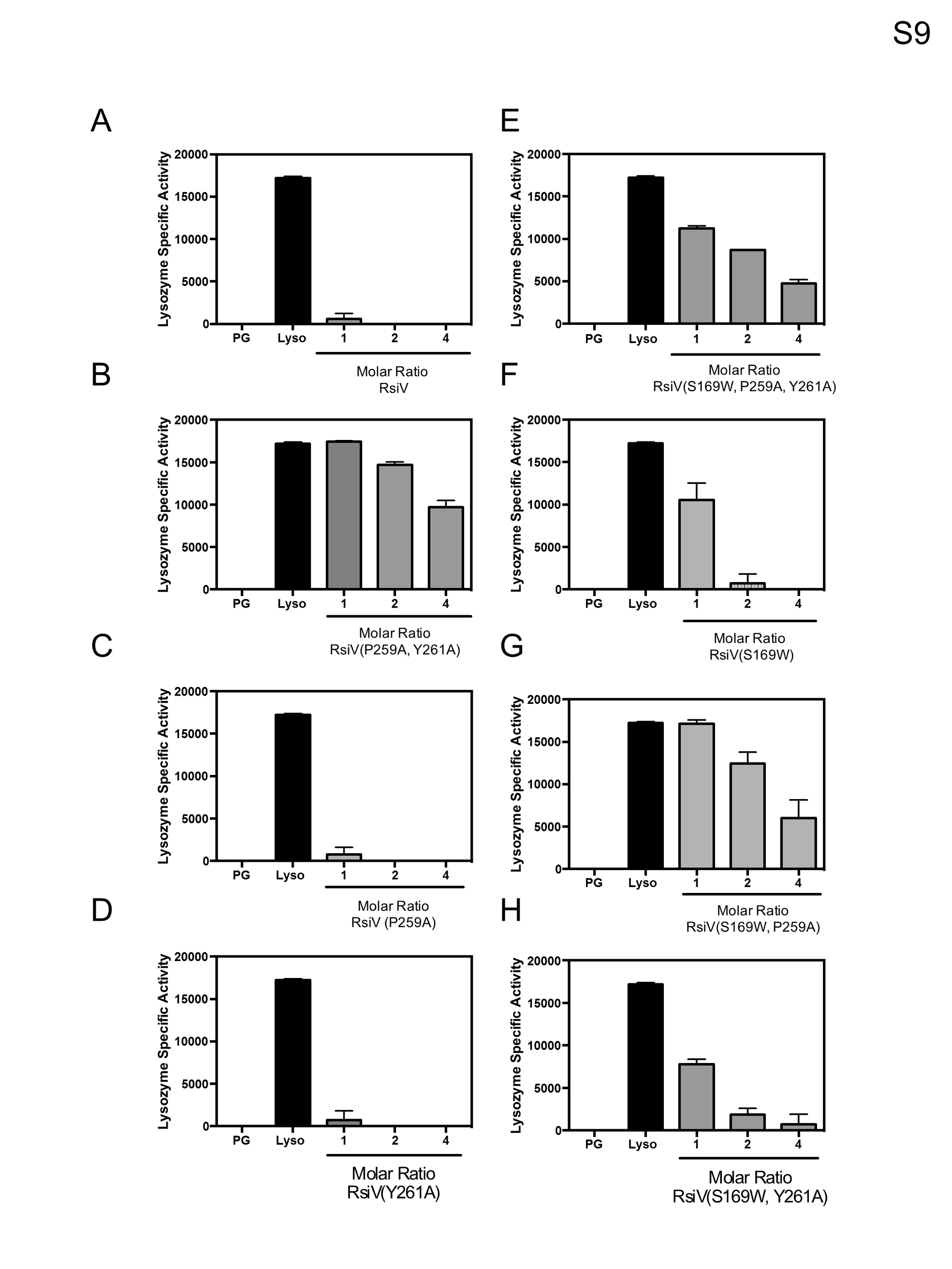

Supplement: S9 Fig — Peptidoglycan from M. lysodekticus was combined with lysozyme (20 μg/ ml) and purified RsiV or RsiV mutants at a molar ratio of 0, 1, 2 or 4. The OD450 was monitored every minute for 30 minutes to determine lysozyme specific activity. A. WT RsiV59-285; B. RsiV59-285(P259A, Y261); C. RsiV59-285(P259A); D. RsiV59-285(Y261A); E. RsiV59-285(S169W, P259A, Y261A); F. RsiV59-285(S169W); G. RsiV59-285(S169W, P259A); and H. RsiV59-285(S169W, Y261A). (TIF) [file pgen.1006287.s009.tif]

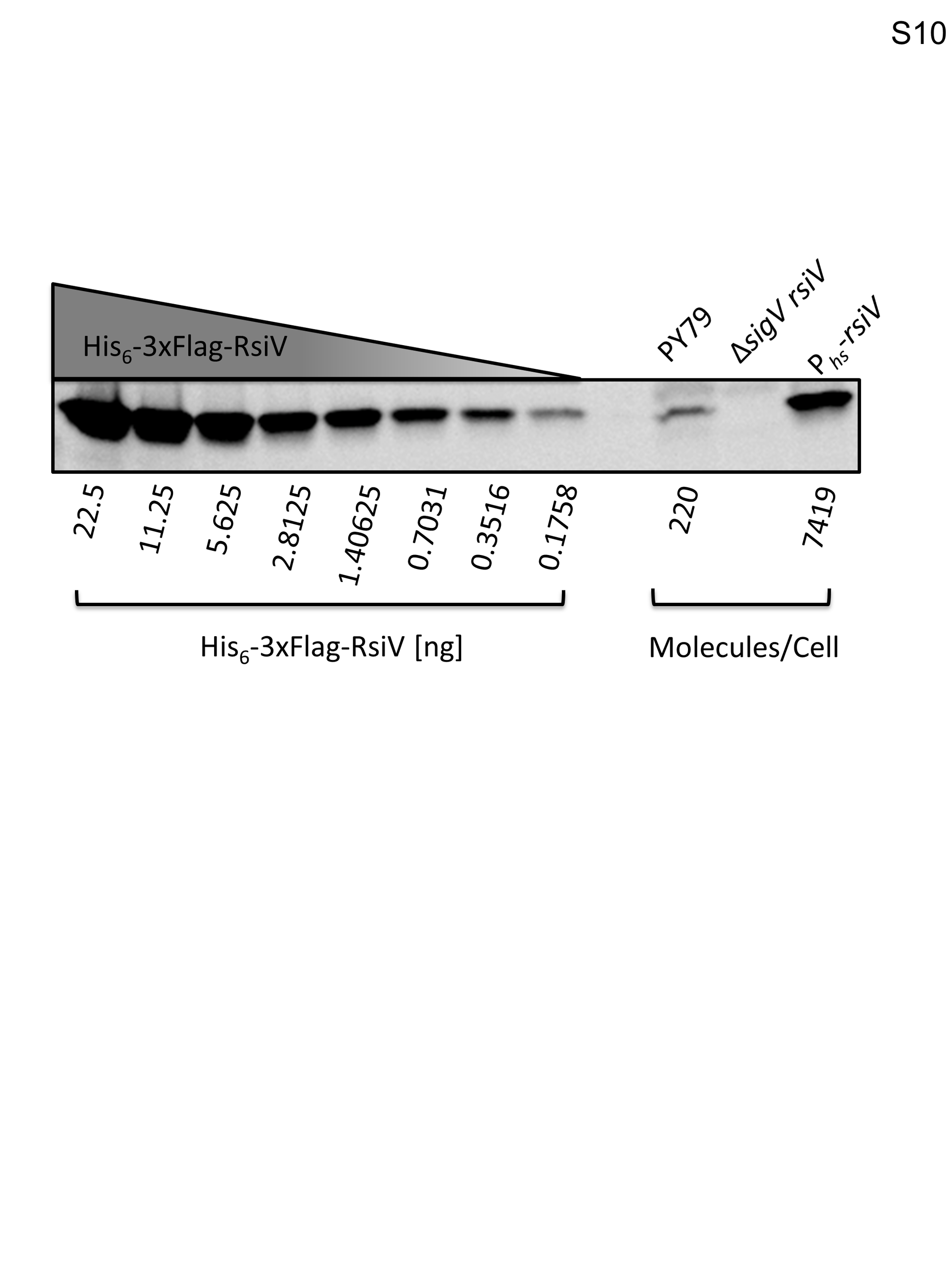

Supplement: S10 Fig — Immunoblot analysis of RsiV protein levels. Overnight B. subtilis strains were subcultured 1:100 into LB WT (PY79) and ΔsigVrsiV (CDE1563) or Phs-rsiV (JLH402) LB + 1mM IPTG and grown to OD600 of 1. Cells were pelleted, resuspended in 100 μl sample buffer, lysed. 2-fold serial dilutions of purified His6-3xFlag-RsiV were loaded were prepared and loaded on an SDS-PAGE gel. Proteins were separated by SDS-PAGE and immunoblotted with anti-RsiV antibodies. (TIF) [file pgen.1006287.s010.tif]
