# Supplementary material for: The Anti-sigma Factor RsiV Is a Bacterial Receptor for Lysozyme: Co-crystal Structure Determination and Demonstration That Binding of Lysozyme to RsiV Is Required for σV Activation
Source: PLoS Genet. 2016 Sep 7;12(9):e1006287. doi: 10.1371/journal.pgen.1006287 (PMC5014341; doi:10.1371/journal.pgen.1006287)
Supplement: S1 Methods — (PDF) [file pgen.1006287.s011.pdf]

Materials and Methods

Co-purification experiments.

Briefly, overnight cultures of *E. coli* BL21λDE3 containing pMRLB.12 were grown at 30°C in LB + ampicillin. The cell cultures were diluted 1:100 into 500 ml of LB + ampicillin in 2 L baffled flasks and incubated at 30°C to an OD_{600} of 0.5–0.6. IPTG was added to a final concentration of 1 mM to induce protein production and the cultures incubated for an additional 4 hours. Cells were chilled on ice and collected by centrifugation at 5000xg. Cell pellets were stored at −80°C until time for purification.

Cell pellets were thawed on ice and resuspended in 5 ml lysis buffer (50 mM Sodium Phosphate, 250 mM NaCl, 10 mM imidazole, pH 8.0) per 500 ml of initial culture volume. Cells were lysed by passaging through a Microfluidics LV1 high shear microfluidizer (Newton, MA) twice. Lysate was centrifuged at 15,000 x *g*, for 30 minutes at 4°C, to pellet cellular debris. Cleared lysate was applied to a nickel affinity column to bind 6xHis-tagged protein (Qiagen). The column was washed with 10 column volumes of wash buffer (50 mM sodium phosphate, 250 mM NaCl, 20 mM imidazole, pH 8.0). After the initial application of wash buffer, 2 ml of 1 mg/ml HEW lysozyme (Sigma Aldrich) was applied to the column and flow through collected. An additional 5 column volumes of wash buffer were applied to remove any unbound HEW lysozyme. Protein was eluted with elution buffer (50 mM Sodium Phosphate, 250 mM NaCl, 250 mM imidazole, pH 8.0) and collected in 0.5 ml fractions. Samples from each fraction were mixed with an equal volume with 2x Laemmli sample buffer and analyzed on 15% SDS-PAGE gels stained with Coomassie brilliant blue.
