# Supplementary material for: The Anti-sigma Factor RsiV Is a Bacterial Receptor for Lysozyme: Co-crystal Structure Determination and Demonstration That Binding of Lysozyme to RsiV Is Required for σV Activation
Source: PLoS Genet. 2016 Sep 7;12(9):e1006287. doi: 10.1371/journal.pgen.1006287 (PMC5014341; doi:10.1371/journal.pgen.1006287)
Supplement: S1 Table — (PDF) [file pgen.1006287.s012.pdf]

Table S1
blastp
Iteration: 0
Query:
RID: KK8J6DHT015
Database: nr
Fields: query id, subject ids, % identity, % positives, alignment length, mismatches, gap opens,
898 hits found

unnamed	gi|16079767	100	100	285	0	0
unnamed	gi|430755846	99.65	100	285	1	0
unnamed	gi|449095149	98.95	99.3	285	3	0
unnamed	gi|516292751	98.6	98.95	285	4	0
unnamed	gi|384176288	98.25	99.3	285	5	0
unnamed	gi|428280155	97.89	98.95	285	6	0
unnamed	gi|545117568	97.89	98.6	285	6	0
unnamed	gi|386759266	97.54	98.6	285	7	0
unnamed	gi|321312225	97.54	97.89	285	7	0
unnamed	gi|518088803	95.79	98.6	285	12	0
unnamed	gi|489330062	95.79	98.6	285	12	0
unnamed	gi|305675297	94.74	97.89	285	15	0
unnamed	gi|350266880	94.74	97.89	285	15	0
unnamed	gi|518544982	94.04	97.19	285	17	0
unnamed	gi|498020924	93.33	97.89	285	19	0
unnamed	gi|515503793	81.47	93.71	286	52	1
unnamed	gi|494883764	81.82	93.01	286	51	1
unnamed	gi|308172506	81.12	93.01	286	53	1
unnamed	gi|568176318	81.47	93.01	286	52	1
unnamed	gi|154685118	81.47	93.01	286	52	1
unnamed	gi|452854625	81.47	93.01	286	52	1
unnamed	gi|545133883	81.47	93.01	286	52	1
unnamed	gi|384264205	81.47	93.01	286	52	1
unnamed	gi|489247651	81.12	93.36	286	53	1
unnamed	gi|530568429	81.12	93.36	286	53	1
unnamed	gi|375361324	81.12	93.01	286	53	1
unnamed	gi|515988421	80.77	93.01	286	54	1
unnamed	gi|498486116	76.49	89.82	285	67	0
unnamed	gi|311067088	76.14	89.82	285	68	0
unnamed	gi|493688775	81.12	90.56	286	53	1
unnamed	gi|489271484	74.48	88.46	286	72	1
unnamed	gi|52079037	74.48	88.11	286	72	1
unnamed	gi|511061459	73.43	88.46	286	75	1

unnamed	gi|584583183	73.78	87.76	286	74	1
unnamed	gi|515136411	55.56	79.51	288	124	2
unnamed	gi|384045510	54.51	79.17	288	127	2
unnamed	gi|294500639	54.86	79.17	288	126	2
unnamed	gi|295705992	55.21	78.82	288	125	2
unnamed	gi|518573218	54.2	73.78	286	129	2
unnamed	gi|494762022	54.2	73.78	286	129	2
unnamed	gi|157694451	53.85	73.78	286	130	2
unnamed	gi|594025497	53.15	73.78	286	132	2
unnamed	gi|489307457	54.2	73.78	286	129	2
unnamed	gi|495622957	53.5	73.78	286	131	2
unnamed	gi|515937874	53.5	73.78	286	131	2
unnamed	gi|495634542	52.63	72.28	285	134	1
unnamed	gi|494213570	48.47	67.12	295	141	3
unnamed	gi|489425691	46.26	69.73	294	148	4
unnamed	gi|169828834	48.65	65.88	296	140	5
unnamed	gi|490350953	48.82	66.33	297	139	6
unnamed	gi|517584129	49.15	65.87	293	140	4
unnamed	gi|495457111	47.65	66.11	298	142	6
unnamed	gi|493232334	46.26	64.63	294	148	3
unnamed	gi|497780557	47.42	65.98	291	145	3
unnamed	gi|573584812	48.31	65.88	296	141	5
unnamed	gi|573531864	46.26	64.29	294	148	3
unnamed	gi|512483909	46.92	65.07	292	146	3
unnamed	gi|385778167	46.31	66.11	298	146	4
unnamed	gi|125973950	46.31	66.11	298	146	4
unnamed	gi|496351565	46.84	66.11	301	144	5
unnamed	gi|518918267	47.84	66.11	301	141	6
unnamed	gi|489422799	72.84	88.89	162	44	0
unnamed	gi|261404313	44.22	64.63	294	154	3
unnamed	gi|518071632	47.92	64.58	288	141	3
unnamed	gi|402574333	46.98	65.44	298	145	6
unnamed	gi|515820194	45.92	66.33	294	143	5
unnamed	gi|499131541	47.3	64.53	296	144	5
unnamed	gi|495684285	47.28	64.97	294	144	4
unnamed	gi|544840271	46.39	67.01	291	148	3
unnamed	gi|497281663	43.54	63.27	294	156	3
unnamed	gi|488642450	46.31	64.43	298	146	3
unnamed	gi|489503137	46.31	64.43	298	146	3
unnamed	gi|573540591	41.75	65.32	297	160	4
unnamed	gi|407475024	46.49	65.22	299	145	4

unnamed	gi|566226671	45.64	64.77	298	148	3
unnamed	gi|497452409	44.83	65.86	290	152	4
unnamed	gi|573585868	48.97	66.21	290	138	4
unnamed	gi|597828674	47.74	63.41	287	137	6
unnamed	gi|573545688	41.61	65.77	298	159	6
unnamed	gi|332798968	44.78	65.32	297	148	4
unnamed	gi|555077424	44.78	65.32	297	148	4
unnamed	gi|515112257	46.49	64.21	299	142	6
unnamed	gi|565878574	45.72	63.57	269	137	3
unnamed	gi|573567428	42.57	63.18	296	158	4
unnamed	gi|495054829	42.09	64.31	297	159	4
unnamed	gi|495683844	41.98	69.97	293	159	6
unnamed	gi|326792987	43.49	64.73	292	153	6
unnamed	gi|494737286	42.31	62.94	286	163	2
unnamed	gi|494811185	41.96	62.94	286	164	2
unnamed	gi|585396013	42.57	60.81	296	158	4
unnamed	gi|431792897	41.69	62.71	295	161	3
unnamed	gi|393201285	41.3	69.62	293	161	6
unnamed	gi|547840824	43.06	62.15	288	158	4
unnamed	gi|89896976	42.81	64.88	299	156	5
unnamed	gi|517015815	42.81	64.55	299	156	5
unnamed	gi|219667154	42.67	64	300	157	5
unnamed	gi|518687989	42.81	64.21	299	156	5
unnamed	gi|557827145	44.67	62.89	291	148	7
unnamed	gi|310659674	45.02	65.98	291	148	5
unnamed	gi|392392627	41.81	63.88	299	159	5
unnamed	gi|573584659	45.24	63.27	294	149	5
unnamed	gi|251796800	42.95	60.74	298	156	4
unnamed	gi|545059256	43.17	62.95	278	145	6
unnamed	gi|510831359	40.7	63.51	285	167	2
unnamed	gi|489515282	45.67	60.55	289	137	8
unnamed	gi|544697571	41.34	62.19	283	154	5
unnamed	gi|545058319	42.09	62.59	278	148	6
unnamed	gi|491693279	49.1	68.92	222	107	2
unnamed	gi|491031489	40.61	61.77	293	157	6
unnamed	gi|126699163	44.79	59.72	288	141	7
unnamed	gi|515099336	44.79	59.72	288	141	7
unnamed	gi|544960748	44.79	59.72	288	141	7
unnamed	gi|289433709	39.31	61.38	290	159	3
unnamed	gi|260683174	44.79	59.72	288	141	7
unnamed	gi|544972424	44.79	59.38	288	141	7

unnamed	gi|544986222	43.01	58.74	286	149	5
unnamed	gi|544969560	44.44	59.38	288	142	7
unnamed	gi|549299325	44.79	59.38	288	141	7
unnamed	gi|489525027	44.44	59.38	288	142	7
unnamed	gi|544955118	44.44	59.38	288	142	7
unnamed	gi|545051670	44.44	59.38	288	142	7
unnamed	gi|347547813	39.51	60.84	286	164	4
unnamed	gi|583837066	39.51	60.84	286	164	4
unnamed	gi|495450326	39.33	57.67	300	166	4
unnamed	gi|493615037	39.18	60.82	291	162	6
unnamed	gi|489507786	40.22	60.52	271	156	3
unnamed	gi|489524924	40.22	60.52	271	156	3
unnamed	gi|488645701	40.22	60.52	271	156	3
unnamed	gi|496596186	38.49	60.82	291	164	6
unnamed	gi|577798833	41.95	61.02	236	136	1
unnamed	gi|517420074	36.62	58.1	284	170	4
unnamed	gi|498524319	35.99	55.36	289	174	4
unnamed	gi|498407749	35.99	55.36	289	174	4
unnamed	gi|498471427	35.99	55.36	289	174	4
unnamed	gi|428768284	35.99	55.36	289	174	4
unnamed	gi|384514411	35.99	55.36	289	174	4
unnamed	gi|488304837	35.99	55.36	289	174	4
unnamed	gi|397701320	35.99	55.36	289	174	4
unnamed	gi|488287789	35.99	55.36	289	174	4
unnamed	gi|488333137	35.99	55.36	289	174	4
unnamed	gi|514898017	35.99	55.02	289	174	4
unnamed	gi|498522125	35.99	55.36	289	174	4
unnamed	gi|498528321	35.99	55.36	289	174	4
unnamed	gi|488313812	35.99	55.02	289	174	4
unnamed	gi|29377629	35.99	55.36	289	174	4
unnamed	gi|514886594	35.99	55.36	289	174	4
unnamed	gi|498399605	35.99	55.36	289	174	4
unnamed	gi|498393274	35.99	55.36	289	174	4
unnamed	gi|498517721	35.99	55.02	289	174	4
unnamed	gi|547301295	37.76	57.34	286	161	8
unnamed	gi|488296265	35.64	55.02	289	175	4
unnamed	gi|573663433	35.64	55.36	289	175	4
unnamed	gi|313630204	39.09	59.26	243	146	2
unnamed	gi|488315781	35.64	55.02	289	175	4
unnamed	gi|488331751	35.64	55.02	289	175	4
unnamed	gi|488674779	38.89	58.68	288	155	9

unnamed	gi|488338459	35.99	54.67	289	174	4
unnamed	gi|488326615	35.64	55.02	289	175	4
unnamed	gi|488323317	36.82	55.6	277	164	4
unnamed	gi|488325353	35.64	55.02	289	175	4
unnamed	gi|488319236	35.29	54.67	289	176	4
unnamed	gi|544986196	41.95	57.2	236	132	3
unnamed	gi|34761997	34.95	54.33	289	177	4
unnamed	gi|546668083	34.46	56.76	296	172	7
unnamed	gi|498266515	35.37	56.12	294	172	6
unnamed	gi|545029898	39.83	56.36	236	138	2
unnamed	gi|544958602	39.83	56.36	236	138	2
unnamed	gi|515099337	39.83	55.93	236	138	2
unnamed	gi|495998060	35.31	53.85	286	172	5
unnamed	gi|544972433	39.41	56.36	236	139	2
unnamed	gi|544967596	39.41	55.93	236	139	2
unnamed	gi|545016443	39.41	55.51	236	139	2
unnamed	gi|544959667	38.98	55.51	236	140	2
unnamed	gi|496653627	32.14	53.25	308	169	6
unnamed	gi|498473699	31.97	56.12	294	182	6
unnamed	gi|489594501	32.14	53.25	308	169	6
unnamed	gi|493485496	33.56	55.14	292	169	6
unnamed	gi|514895291	37.12	56.44	264	155	6
unnamed	gi|511046877	36.1	54.31	313	170	7
unnamed	gi|495142965	34.97	54.2	286	169	6
unnamed	gi|544962094	39.41	55.08	236	139	2
unnamed	gi|514885634	35.64	54.67	289	175	4
unnamed	gi|488262913	31.63	55.78	294	183	6
unnamed	gi|488246683	31.63	55.78	294	183	6
unnamed	gi|497582333	38.98	55.08	236	140	2
unnamed	gi|494983815	33.69	56.38	282	176	6
unnamed	gi|383329398	31.63	55.78	294	183	6
unnamed	gi|544957514	38.56	55.51	236	141	2
unnamed	gi|389869256	31.63	55.78	294	183	6
unnamed	gi|497588389	38.56	55.51	236	141	2
unnamed	gi|488234985	31.63	55.78	294	183	6
unnamed	gi|489531927	38.56	55.51	236	141	2
unnamed	gi|126699164	38.56	55.51	236	141	2
unnamed	gi|498421399	31.63	55.78	294	183	6
unnamed	gi|488307533	31.63	55.78	294	183	6
unnamed	gi|545047922	38.98	55.08	236	140	2
unnamed	gi|545026583	38.14	55.51	236	142	2

unnamed	gi|544953439	38.56	55.08	236	141	2
unnamed	gi|493835465	36.01	56.64	286	172	5
unnamed	gi|488223920	31.63	55.44	294	183	6
unnamed	gi|544978114	38.56	55.08	236	141	2
unnamed	gi|514881775	34.23	57.31	260	166	3
unnamed	gi|549299326	45.21	60.11	188	99	2
unnamed	gi|498408802	32.55	55.37	298	177	7
unnamed	gi|547313952	35.37	52.41	311	165	8
unnamed	gi|544955119	38.14	55.08	236	142	2
unnamed	gi|392988891	32.55	55.03	298	177	7
unnamed	gi|486822861	33.33	56.2	258	160	5
unnamed	gi|514892960	33.46	57.31	260	168	3
unnamed	gi|488273240	32.95	56.59	258	161	5
unnamed	gi|491375159	33.46	57.31	260	168	3
unnamed	gi|488237342	32.95	56.59	258	161	5
unnamed	gi|495657240	33.46	57.31	260	168	3
unnamed	gi|488274068	31.6	56.13	269	173	5
unnamed	gi|488221615	32.95	56.59	258	161	5
unnamed	gi|488243457	32.95	56.59	258	161	5
unnamed	gi|488264793	32.95	56.59	258	161	5
unnamed	gi|488249063	32.95	56.59	258	161	5
unnamed	gi|260683175	37.29	55.08	236	144	2
unnamed	gi|498415641	32.95	56.59	258	161	5
unnamed	gi|488269692	32.95	56.59	258	161	5
unnamed	gi|544963322	38.56	54.24	236	141	2
unnamed	gi|514906238	32.95	56.59	258	161	5
unnamed	gi|488271646	32.56	56.2	258	162	5
unnamed	gi|488229246	32.56	56.59	258	162	5
unnamed	gi|547940239	33.45	56.76	296	181	9
unnamed	gi|547848725	35.16	52.01	273	166	6
unnamed	gi|488637362	35.19	54.36	287	173	6
unnamed	gi|488661184	35.19	54.36	287	173	6
unnamed	gi|488634551	35.19	54.36	287	173	6
unnamed	gi|547964957	35.14	51.45	276	168	6
unnamed	gi|510872560	33.77	53.57	308	171	8
unnamed	gi|258515768	34.04	56.03	282	176	6
unnamed	gi|488632769	34.84	54.36	287	174	6
unnamed	gi|494096915	35.21	54.23	284	171	6
unnamed	gi|547791021	35.69	54.77	283	169	6
unnamed	gi|488647833	35.69	54.77	283	169	6
unnamed	gi|589402467	35.79	57.19	285	166	9

unnamed	gi|54679876!	34.84	54.36	287	174	6
unnamed	gi|49069648:	33.45	51.22	287	168	6
unnamed	gi|48952502!	44.09	58.6	186	100	2
unnamed	gi|51080260!	31.08	53.72	296	183	7
unnamed	gi|48942280:	81.25	91.25	80	15	0
unnamed	gi|49253932(	30.33	52.33	300	180	6
unnamed	gi|49844698!	33.74	53.66	246	153	3
unnamed	gi|55868525!	32.87	56.4	289	180	6
unnamed	gi|54783481!	31.85	58.56	292	183	7
unnamed	gi|51103338:	32.08	49.06	318	175	7
unnamed	gi|30238672:	34.16	54.09	281	172	7
unnamed	gi|54564017!	36.55	53.41	249	148	5
unnamed	gi|54825917:	32.01	54.32	278	176	6
unnamed	gi|49843050:	32.18	56.06	289	182	6
unnamed	gi|49295036:	29.89	52.31	281	182	5
unnamed	gi|54746791-	36.44	56.44	225	129	5
unnamed	gi|49806084:	31.36	49.83	287	174	6
unnamed	gi|54504399:	51.95	62.99	154	69	3
unnamed	gi|49093553(	28.47	54.17	288	195	5
unnamed	gi|49391030:	35.14	50.34	296	166	6
unnamed	gi|49016626-	32.88	55.48	292	181	7
unnamed	gi|47914803!	35.14	50.34	296	166	6
unnamed	gi|47920257!	35.14	50.34	296	166	6
unnamed	gi|51105247:	33.76	51.91	314	175	10
unnamed	gi|54788503-	35.51	54.21	214	129	3
unnamed	gi|54647292!	33.92	53	283	174	6
unnamed	gi|49339923!	30.47	51.25	279	180	3
unnamed	gi|54773079-	34.69	51.43	245	142	5
unnamed	gi|49382936:	40.31	58.64	191	105	5
unnamed	gi|54731331!	33.6	55.34	253	149	7
unnamed	gi|49657118:	29.49	53.22	295	186	6
unnamed	gi|54721301(	32.29	55.21	288	177	8
unnamed	gi|54790998:	31.27	49.85	323	169	10
unnamed	gi|54832906:	32.4	52.4	250	153	4
unnamed	gi|49094142!	33.73	54.22	249	152	6
unnamed	gi|54821953!	35.86	54.48	290	172	7
unnamed	gi|54566841(	33.76	51.28	234	147	5
unnamed	gi|49650759-	31.78	50	258	161	6
unnamed	gi|51102977:	33.33	53.33	240	143	5
unnamed	gi|54640509:	28.42	48.63	292	193	7
unnamed	gi|54774018!	30.67	50	326	172	9

unnamed	gi|49361909[illegible]	30.88	53.68	285	180	6
unnamed	gi|54786796[illegible]	34.07	53.1	226	144	4
unnamed	gi|51176380[illegible]	35.25	52.05	244	145	6
unnamed	gi|31312446[illegible]	35.66	51.64	244	144	6
unnamed	gi|48971187[illegible]	35.66	51.64	244	144	6
unnamed	gi|11651469[illegible]	35.92	52.24	245	142	7
unnamed	gi|49539368[illegible]	30.98	48.48	297	166	10
unnamed	gi|34753148[illegible]	31.4	52.07	242	144	5
unnamed	gi|53785424[illegible]	31.27	46.72	259	155	6
unnamed	gi|54818783[illegible]	28.98	51.94	283	180	7
unnamed	gi|48970801[illegible]	35.51	52.24	245	143	7
unnamed	gi|16087972[illegible]	27.72	49.82	285	196	6
unnamed	gi|48868533[illegible]	27.21	51.36	294	194	6
unnamed	gi|48971790[illegible]	35.51	51.84	245	143	7
unnamed	gi|49383163[illegible]	31.31	51.18	297	169	9
unnamed	gi|54789582[illegible]	27.21	51.02	294	194	6
unnamed	gi|49656975[illegible]	27.21	51.02	294	194	6
unnamed	gi|49475469[illegible]	28.07	50.18	285	189	5
unnamed	gi|54540156[illegible]	27.39	46.5	314	181	6
unnamed	gi|49654887[illegible]	27.52	48.66	298	174	7
unnamed	gi|37429794[illegible]	32.62	54.08	233	143	5
unnamed	gi|54712686[illegible]	28.16	51.13	309	188	7
unnamed	gi|49513444[illegible]	28.47	50.51	295	178	8
unnamed	gi|49609232[illegible]	27.21	50.68	294	194	6
unnamed	gi|54539483[illegible]	27.92	47.4	308	170	8
unnamed	gi|49655124[illegible]	27.72	49.82	285	190	5
unnamed	gi|54796103[illegible]	30.64	48.48	297	167	10
unnamed	gi|54824687[illegible]	32.63	50.42	236	143	4
unnamed	gi|49727439[illegible]	26.87	50.68	294	195	6
unnamed	gi|54794440[illegible]	27.46	51.76	284	183	8
unnamed	gi|10477457[illegible]	35.1	51.84	245	144	7
unnamed	gi|48972029[illegible]	35.1	51.84	245	144	7
unnamed	gi|54469202[illegible]	56.67	74.44	90	37	1
unnamed	gi|49567377[illegible]	30.6	46.64	268	156	6
unnamed	gi|54820480[illegible]	40.67	59.33	150	84	2
unnamed	gi|49573728[illegible]	31.66	49.03	259	161	6
unnamed	gi|49724365[illegible]	32.05	49.03	259	160	7
unnamed	gi|47933869[illegible]	39.16	54.55	143	86	1
unnamed	gi|49355194[illegible]	41.48	62.96	135	78	1
unnamed	gi|49383712[illegible]	28.83	49.47	281	181	6
unnamed	gi|49729280[illegible]	28.83	49.47	281	181	6

unnamed	gi|49567773	29.11	47.6	292	170	8
unnamed	gi|54720348	29.11	47.6	292	170	8
unnamed	gi|54538968	30.3	51.08	231	148	4
unnamed	gi|49599285	25.84	47.65	298	185	7
unnamed	gi|49499377	28.57	50.34	294	175	9
unnamed	gi|48959591	29.83	48.32	238	146	5
unnamed	gi|49665640	29.83	48.32	238	146	5
unnamed	gi|54794495	32.73	54.09	220	133	7
unnamed	gi|49606367	27.21	49.12	283	185	5
unnamed	gi|58940189	30.17	46.78	295	173	8
unnamed	gi|49598613	29.63	53.97	189	125	3
unnamed	gi|54772626	32.73	52.27	220	132	5
unnamed	gi|54746941	33.1	53.1	290	167	9
unnamed	gi|51217613	30.04	51.57	223	135	6
unnamed	gi|48863602	25.16	46.27	322	197	9
unnamed	gi|48862830	25.16	46.58	322	197	9
unnamed	gi|56563269	29.72	53.3	212	142	3
unnamed	gi|54788376	26.86	49.47	283	182	10
unnamed	gi|49567277	28.04	52.03	296	176	13
unnamed	gi|54722948	28.38	52.03	296	175	13
unnamed	gi|54818170	31.34	52.74	201	126	5
unnamed	gi|47933738	27.7	51.69	296	177	13
unnamed	gi|59782818	31.54	45.77	260	154	6
unnamed	gi|49698917	25.74	45.22	272	158	4
unnamed	gi|48951058	29.83	45.8	238	152	4
unnamed	gi|49721825	26.3	51.11	270	178	9
unnamed	gi|49722019	27.44	47.74	266	153	6
unnamed	gi|49722221	25.37	44.85	272	159	4
unnamed	gi|49383712	32.5	52.5	200	115	6
unnamed	gi|54796460	26.86	45.63	309	178	9
unnamed	gi|54784754	26.54	44.66	309	179	8
unnamed	gi|54771824	25.1	50.59	255	173	5
unnamed	gi|54504399	38.46	58.46	130	73	3
unnamed	gi|49426990	28.21	45.71	280	165	7
unnamed	gi|51108154	24.56	44.84	281	191	8
unnamed	gi|48866150	24.12	45.02	311	188	8
unnamed	gi|49567154	25.51	48.56	243	154	7
unnamed	gi|48863858	24.12	45.02	311	188	8
unnamed	gi|54722847	25.51	48.56	243	154	7
unnamed	gi|49606541	30.3	50.22	231	140	7
unnamed	gi|54730129	27.19	47.81	228	146	6

unnamed	gi|551221491	24.24	42.93	198	133	4
unnamed	gi|568265557	32.63	49.47	190	109	8
unnamed	gi|493829358	26.75	47.37	228	147	6
unnamed	gi|551220750	23.27	48.51	202	135	4
unnamed	gi|496267971	23.57	45.71	280	192	7
unnamed	gi|488674781	26.75	47.81	228	147	6
unnamed	gi|547945004	26.2	46.72	229	141	7
unnamed	gi|545625047	22.18	46.13	284	193	9
unnamed	gi|565896727	29.75	50.41	242	138	11
unnamed	gi|497361739	28.42	45.79	190	112	7
unnamed	gi|150015190	29.75	50.41	242	138	11
unnamed	gi|134300664	30.24	47.18	248	150	9
unnamed	gi|573547450	30.85	43.78	201	98	7
unnamed	gi|114567141	28.92	46.18	249	118	11
unnamed	gi|326791267	27.38	45.63	252	152	8
unnamed	gi|515112889	33.71	45.71	175	97	6
unnamed	gi|226315158	33.71	45.71	175	97	6
unnamed	gi|518209861	31.02	47.06	187	110	6
unnamed	gi|515821725	33.14	45.71	175	98	6
unnamed	gi|494994022	33.14	45.71	175	98	6
unnamed	gi|515777149	29.75	48.76	242	138	11
unnamed	gi|496694333	31.02	46.52	187	110	6
unnamed	gi|517588171	30.5	47.5	200	118	8
unnamed	gi|488776529	28.97	47.66	214	129	7
unnamed	gi|495052487	32	44	175	100	6
unnamed	gi|492385856	32	44	175	100	6
unnamed	gi|559040262	29.71	47.43	175	104	6
unnamed	gi|384455273	30.43	48.79	207	122	9
unnamed	gi|28211483	26.61	47.98	248	158	8
unnamed	gi|494705205	30.58	49.03	206	121	9
unnamed	gi|342731843	30.58	49.03	206	121	9
unnamed	gi|492951201	23.36	44.53	274	184	7
unnamed	gi|492386187	27.8	41.31	259	151	9
unnamed	gi|495061253	27.8	41.31	259	151	9
unnamed	gi|496068178	30.43	58.26	115	76	3
unnamed	gi|334339695	30.65	47.74	199	113	9
unnamed	gi|489635106	30.43	58.26	115	76	3
unnamed	gi|550156777	31.53	50.25	203	118	9
unnamed	gi|557605903	27.02	47.58	248	157	8
unnamed	gi|251796724	29	44.5	200	121	7
unnamed	gi|332800287	31.16	46.73	199	110	8

unnamed	gi|49736242	27.5	45.5	200	124	8
unnamed	gi|58392088	32.02	45.51	178	102	6
unnamed	gi|48943577	30.54	50.74	203	120	9
unnamed	gi|31065775	33.66	47.8	205	116	9
unnamed	gi|49135667	39.24	67.09	79	44	3
unnamed	gi|54745148	27.63	45.14	257	143	10
unnamed	gi|48952030	30.43	47.39	230	131	11
unnamed	gi|48951306	30.87	47.83	230	130	11
unnamed	gi|49557506	29.05	45.25	179	108	6
unnamed	gi|54502623	30.43	47.39	230	131	11
unnamed	gi|54538283	28.16	48.06	206	124	8
unnamed	gi|51107158	28.57	48.81	168	94	4
unnamed	gi|54496166	30.43	47.39	230	131	11
unnamed	gi|49449696	27.63	45.14	257	143	10
unnamed	gi|54496844	30.43	47.39	230	131	11
unnamed	gi|26068224	30.43	47.39	230	131	11
unnamed	gi|12669812	30.43	47.39	230	131	11
unnamed	gi|54497178	30.43	47.39	230	131	11
unnamed	gi|55091682	29.95	49.24	197	118	8
unnamed	gi|51509934	30.43	47.39	230	131	11
unnamed	gi|26140640	27.36	47.76	201	125	9
unnamed	gi|49421493	28.86	46.77	201	122	8
unnamed	gi|48951528	42.47	58.9	73	37	2
unnamed	gi|54505859	32.46	50.26	191	102	7
unnamed	gi|48948509	26.74	45.99	187	118	7
unnamed	gi|57357456	34.44	57.78	90	59	0
unnamed	gi|51795074	31.49	45.86	181	105	8
unnamed	gi|49570224	31.02	50.27	187	109	9
unnamed	gi|49595643	29.9	49.51	204	103	9
unnamed	gi|54505529	32.46	50.26	191	102	7
unnamed	gi|51594865	29.03	42.47	186	113	6
unnamed	gi|45181721	28.71	48.51	202	122	9
unnamed	gi|48941764	28.72	49.23	195	106	7
unnamed	gi|51581673	25.23	49.07	214	120	10
unnamed	gi|28855520	28.65	44.86	185	107	6
unnamed	gi|48864610	31.85	51.11	135	78	5
unnamed	gi|48950595	31.58	51.13	133	81	4
unnamed	gi|48952520	33.09	50.74	136	69	5
unnamed	gi|25179667	31.61	45.98	174	100	7
unnamed	gi|49645915	28.34	50.8	187	114	7
unnamed	gi|51248512	28.22	42.08	202	110	7

unnamed	gi|547847548	29.53	49.66	149	94	4
unnamed	gi|517499696	29.69	42.71	192	114	7
unnamed	gi|331270593	29.65	45.73	199	117	7
unnamed	gi|599128647	29.51	45.36	183	114	6
unnamed	gi|489493583	25.48	46.63	208	133	8
unnamed	gi|544868809	30.6	47.54	183	106	7
unnamed	gi|497279296	27.27	46.52	187	117	8
unnamed	gi|550549850	28.11	44.32	185	108	6
unnamed	gi|158319153	28.14	48.24	199	115	8
unnamed	gi|492906410	29.65	43.02	172	106	4
unnamed	gi|516287724	33.33	61.29	93	56	3
unnamed	gi|544984997	32.85	48.91	137	82	4
unnamed	gi|517588884	26.8	47.42	194	121	6
unnamed	gi|547295219	27.08	49.48	192	129	6
unnamed	gi|148378990	30.53	44.74	190	111	8
unnamed	gi|494213140	32.26	59.14	93	61	2
unnamed	gi|517205489	30.96	44.16	197	114	8
unnamed	gi|517420486	31.55	50.6	168	93	7
unnamed	gi|153932938	30.17	44.13	179	106	7
unnamed	gi|489483459	28.92	41.18	204	103	10
unnamed	gi|384047329	29.32	49.74	191	115	8
unnamed	gi|591273656	27.51	48.68	189	117	6
unnamed	gi|515984483	29.35	46.27	201	121	9
unnamed	gi|160879197	25.94	43.69	293	181	8
unnamed	gi|522105063	28.74	46.11	167	104	5
unnamed	gi|545381561	26.95	43.75	256	157	10
unnamed	gi|114567277	25.12	47.87	211	134	7
unnamed	gi|489549883	27.15	47.51	221	138	9
unnamed	gi|491697704	32.35	47.79	136	66	5
unnamed	gi|494996204	32.58	55.06	89	53	2
unnamed	gi|518363750	27.72	44.57	184	108	8
unnamed	gi|559779962	29.8	44.44	198	118	7
unnamed	gi|496754675	25.98	47.06	204	129	8
unnamed	gi|515821621	30	45	200	116	9
unnamed	gi|544692019	34.44	55.56	90	55	1
unnamed	gi|220932336	29.61	47.49	179	106	7
unnamed	gi|496184387	29.47	50.53	190	114	8
unnamed	gi|497880200	28.12	44.79	192	119	7
unnamed	gi|544846168	32.35	47.06	136	66	5
unnamed	gi|493232798	36	65.33	75	46	2
unnamed	gi|573531639	36	65.33	75	46	2

unnamed	gi|14767869	25.7	45.33	214	139	8
unnamed	gi|49717427	25.56	45.56	180	115	6
unnamed	gi|49499949	29.08	44.9	196	117	8
unnamed	gi|51846984	26.29	44.85	194	124	6
unnamed	gi|49336610	26.47	46.08	204	128	8
unnamed	gi|57354337	38.96	62.34	77	41	3
unnamed	gi|57354262	26.87	45.77	201	126	8
unnamed	gi|48946190	25.73	47.09	206	130	7
unnamed	gi|51771458	36.19	51.43	105	57	4
unnamed	gi|51758803	35.11	58.51	94	54	4
unnamed	gi|51511110	30.23	45.58	215	121	11
unnamed	gi|56587760	30.22	44.6	139	84	5
unnamed	gi|22631506	30.23	45.12	215	121	11
unnamed	gi|18793543	28.79	41.25	257	142	11
unnamed	gi|48955777	26.7	47.51	221	139	9
unnamed	gi|56588519	34.48	57.47	87	55	2
unnamed	gi|48958700	29.8	43.43	198	118	7
unnamed	gi|49135313	29.37	51.75	143	90	5
unnamed	gi|51598659	28.77	46.58	219	115	10
unnamed	gi|48958488	30	43.16	190	112	7
unnamed	gi|51741982	40.43	58.51	94	46	4
unnamed	gi|48949747	29.47	42.63	190	113	7
unnamed	gi|49323611	26.37	45.27	201	127	8
unnamed	gi|17075526	29.47	42.63	190	113	7
unnamed	gi|55016131	27.6	42.19	192	118	8
unnamed	gi|51824635	25.49	46.57	204	130	8
unnamed	gi|51795249	28.44	45.87	218	134	10
unnamed	gi|11079911	26.24	47.51	221	140	9
unnamed	gi|48942880	27.6	42.19	192	118	8
unnamed	gi|38672512	21.1	39.45	218	116	5
unnamed	gi|37972237	21.1	39.45	218	116	5
unnamed	gi|15613580	30.05	42.08	183	99	7
unnamed	gi|54483849	26.92	43.96	182	102	6
unnamed	gi|21263802	25.98	45.59	204	129	8
unnamed	gi|57353334	36.36	61.04	77	43	3
unnamed	gi|33774944	21.1	39.45	218	116	5
unnamed	gi|11080227	27.73	45	220	138	7
unnamed	gi|54469653	29.9	50	194	104	9
unnamed	gi|57508259	28.36	44.28	201	125	7
unnamed	gi|49796892	29.21	44.94	178	107	6
unnamed	gi|48943558	27.6	42.19	192	118	8

unnamed	gi|54470313(	28.95	43.16	190	114	7
unnamed	gi|51592146	28.02	41.63	257	144	11
unnamed	gi|22092936	27.38	44.44	252	140	12
unnamed	gi|38781728	28.95	43.16	190	114	7
unnamed	gi|48955333(	26.24	47.51	221	140	9
unnamed	gi|22694827	28.42	44.74	190	115	8
unnamed	gi|55760540(	31.07	44.07	177	107	6
unnamed	gi|49736165	23.01	43.81	226	137	8
unnamed	gi|48957059	26.24	47.06	221	140	9
unnamed	gi|49818758	22.31	43.82	251	156	9
unnamed	gi|56961848	25.67	43.85	187	102	7
unnamed	gi|55015466	28.32	47.79	113	62	2
unnamed	gi|51643695	25.12	48.79	207	122	10
unnamed	gi|54615842	24.9	43.27	245	134	8
unnamed	gi|48943228	28.32	47.79	113	62	2
unnamed	gi|48943927	28.32	47.79	113	62	2
unnamed	gi|48810260	26.45	42.98	242	149	9
unnamed	gi|40835768	30.9	45.51	178	104	6
unnamed	gi|48957786	27.07	45.85	229	128	10
unnamed	gi|51750228	27.84	48.45	97	55	1
unnamed	gi|18858772	30.07	48.25	143	83	6
unnamed	gi|49609991	28.5	45.89	207	126	8
unnamed	gi|54657132	24.88	40.09	217	106	5
unnamed	gi|48946687	30.07	48.25	143	83	6
unnamed	gi|57353235	27.6	45.31	192	118	8
unnamed	gi|51628492	27	45	200	125	8
unnamed	gi|28211046	31.64	44.07	177	106	6
unnamed	gi|49372613	27.22	42.78	180	111	6
unnamed	gi|51680497(	29.06	47.29	203	104	11
unnamed	gi|50702455	26.45	42.56	242	149	9
unnamed	gi|44673309	26.14	43.57	241	149	8
unnamed	gi|38672140	29.1	42.86	189	114	8
unnamed	gi|49057872	28.16	46.6	206	124	8
unnamed	gi|54484686	25.63	41.52	277	168	11
unnamed	gi|44673340(	25.61	42.68	246	154	8
unnamed	gi|55762169	25.61	42.68	246	154	8
unnamed	gi|58445525	25	41.8	256	158	8
unnamed	gi|15394017	27.93	41.9	179	110	6
unnamed	gi|49059429	25.34	47.06	221	142	9
unnamed	gi|57354568	40.28	61.11	72	32	4
unnamed	gi|29449886	27.13	48.94	188	117	8

unnamed	gi|54775409	29.9	45.88	194	116	10
unnamed	gi|59011003	27.37	43.68	190	106	9
unnamed	gi|44673308	25.61	42.68	246	154	8
unnamed	gi|49787835	34.15	57.32	82	50	2
unnamed	gi|51798649	27.91	40.7	172	111	4
unnamed	gi|48807371	26.02	41.46	246	153	9
unnamed	gi|57354901	27.08	44.27	192	119	8
unnamed	gi|50703480	26.45	42.56	242	149	9
unnamed	gi|49791990	27.89	45.79	190	117	7
unnamed	gi|48799592	26.47	42.44	238	146	9
unnamed	gi|54486916	30	63.75	80	54	2
unnamed	gi|51758441	28.18	45.3	181	111	7
unnamed	gi|17076056	28.42	42.63	190	115	7
unnamed	gi|29570418	27.13	48.94	188	117	8
unnamed	gi|55016057	25.94	45.75	212	121	10
unnamed	gi|44673309	26.14	43.15	241	149	8
unnamed	gi|11848000	26.14	43.15	241	149	8
unnamed	gi|52140608	26.14	43.15	241	149	8
unnamed	gi|49480746	26.14	43.15	241	149	8
unnamed	gi|49470724	30.26	50.66	152	90	7
unnamed	gi|37626882	26.14	43.15	241	149	8
unnamed	gi|18311293	25.34	47.06	221	142	9
unnamed	gi|51513511	27.53	48.88	178	110	7
unnamed	gi|58539601	26.64	45.56	259	152	13
unnamed	gi|22586689	26.14	43.15	241	149	8
unnamed	gi|40747306	29.5	47.48	139	85	5
unnamed	gi|44673309	26.14	42.32	241	149	8
unnamed	gi|49651670	23.26	40.7	258	136	8
unnamed	gi|51825370	27.22	43.89	180	111	6
unnamed	gi|18858490	32.17	54.78	115	68	5
unnamed	gi|18858931	27.08	47.92	144	81	6
unnamed	gi|33774527	28.57	42.86	189	115	8
unnamed	gi|51643776	30.23	49.61	129	78	5
unnamed	gi|51513625	25	45.83	192	121	8
unnamed	gi|40691453	22.9	45.79	214	136	9
unnamed	gi|29570565	25.39	46.11	193	119	9
unnamed	gi|48954216	34.29	54.29	105	59	4
unnamed	gi|51613107	26.16	43.46	237	146	8
unnamed	gi|48943140	30.05	50.74	203	121	9
unnamed	gi|49421357	22.36	43.88	237	159	9
unnamed	gi|30105640	26.14	42.74	241	149	8

unnamed	gi|54723320	28.16	46.6	206	112	10
unnamed	gi|48938317	26.05	43.28	238	147	8
unnamed	gi|44673504	26.97	43.15	241	147	9
unnamed	gi|48805830	26.45	42.56	242	149	9
unnamed	gi|48938898	26.05	43.28	238	147	8
unnamed	gi|48793028	26.05	43.28	238	147	8
unnamed	gi|40973978	34.23	43.24	111	63	4
unnamed	gi|48877270	33.33	54.29	105	60	4
unnamed	gi|48805300	26.75	41.98	243	147	10
unnamed	gi|51598628	32.88	63.01	73	47	2
unnamed	gi|51758163	25.93	43.92	189	117	7
unnamed	gi|48804119	26.03	42.15	242	150	9
unnamed	gi|48808864	26.03	42.15	242	150	9
unnamed	gi|54505972	30.08	50.38	133	77	6
unnamed	gi|48801789	26.45	42.56	242	149	9
unnamed	gi|49836303	28.08	47.95	146	87	5
unnamed	gi|48809606	26.45	42.56	242	149	9
unnamed	gi|30287322	30.43	48.91	184	102	10
unnamed	gi|48946843	23.67	47.34	207	125	9
unnamed	gi|31320303	32.54	45.24	126	79	4
unnamed	gi|50700732	25.41	41.8	244	157	9
unnamed	gi|18793455	24.06	47.64	212	118	10
unnamed	gi|51592180	24.06	47.64	212	118	10
unnamed	gi|49467263	35.62	58.9	73	41	4
unnamed	gi|57354059	38.89	59.72	72	33	4
unnamed	gi|54486991	23.26	41.86	258	174	8
unnamed	gi|30264958	25.73	43.15	241	150	8
unnamed	gi|57358740	31.71	54.88	82	50	3
unnamed	gi|56880938	30.68	54.55	88	58	2
unnamed	gi|44673308	24.79	42.15	242	153	8
unnamed	gi|30085511	25.53	42.55	188	124	6
unnamed	gi|57354857	35.48	54.84	93	55	4
unnamed	gi|54483908	26.13	45.23	199	120	7
unnamed	gi|16394263	26.02	41.87	246	153	9
unnamed	gi|51875202	32.38	44.76	105	61	4
unnamed	gi|51868854	39.66	62.07	58	34	1
unnamed	gi|39239339	39.66	62.07	58	34	1
unnamed	gi|48796679	25.89	42.64	197	121	6
unnamed	gi|29450030	24.87	46.11	193	120	9
unnamed	gi|54470459	27.37	43.16	190	117	7
unnamed	gi|23779429	27.37	43.16	190	117	7

unnamed	gi|43179212	41.38	60.34	58	33	1
unnamed	gi|48794240	26.47	42.44	238	146	9
unnamed	gi|49756415	25.63	43.28	238	148	8
unnamed	gi|51104876	32.88	60.27	73	49	0
unnamed	gi|51104876	27	46	100	62	2
unnamed	gi|44280462	26.74	42.78	187	117	7
unnamed	gi|44673308	27.27	43.94	198	117	8
unnamed	gi|54487884	27.08	44.27	192	124	7
unnamed	gi|48796123	26.47	42.44	238	146	9
unnamed	gi|48950313	34.29	58.57	70	44	2
unnamed	gi|54657528	30.95	46.83	126	71	6
unnamed	gi|48793539	25.63	42.86	238	148	8
unnamed	gi|38404580	24.87	45.08	193	120	9
unnamed	gi|37432128	33.8	57.75	71	45	2
unnamed	gi|44673308	27.27	43.94	198	117	8
unnamed	gi|48800160	25.89	42.13	197	121	6
unnamed	gi|49836251	29.11	53.16	79	51	2
unnamed	gi|49075838	26.51	55.42	83	51	3
unnamed	gi|38418897	25.2	41.06	246	155	8
unnamed	gi|48790066	25.51	42.91	247	153	10
unnamed	gi|44673306	25.93	43.21	243	149	10
unnamed	gi|54505745	42.86	60.71	56	31	1
unnamed	gi|49414472	28.49	42.46	179	106	7
unnamed	gi|12597258	27.47	44.51	182	110	8
unnamed	gi|11844328	23.9	43.41	205	135	6
unnamed	gi|54780251	26.56	45.31	192	123	8
unnamed	gi|54469218	29.23	50.77	130	80	5
unnamed	gi|51701416	32.86	62.86	70	42	1
unnamed	gi|89893930	32.86	62.86	70	42	1
unnamed	gi|49233802	32.86	62.86	70	42	1
unnamed	gi|13429998	27.14	47.74	199	121	8
unnamed	gi|55903613	27.5	46.5	200	115	11
unnamed	gi|54558825	24.34	46.03	189	101	9
unnamed	gi|42784101	24.79	42.15	242	153	8
unnamed	gi|50705987	26.45	42.15	242	149	9
unnamed	gi|44673312	26.45	42.15	242	149	9
unnamed	gi|44673339	25.2	41.87	246	155	8
unnamed	gi|44673307	25.2	42.28	246	155	9
unnamed	gi|54541724	33.33	49.52	105	60	4
unnamed	gi|50706511	25	41.39	244	158	9
unnamed	gi|55437780	25.53	40.96	188	124	6

unnamed	gi|44673308[illegible]	24.8	41.46	246	156	8
unnamed	gi|51083583[illegible]	28.57	58.57	70	50	0
unnamed	gi|21796238[illegible]	25.73	41.91	241	150	8
unnamed	gi|58539617[illegible]	25	44.9	196	131	6
unnamed	gi|51382671[illegible]	26.02	41.06	246	153	9
unnamed	gi|51598568[illegible]	24.35	47.67	193	120	8
unnamed	gi|51598568[illegible]	32.8	49.6	125	64	6
unnamed	gi|40256358[illegible]	24.8	41.06	246	156	8
unnamed	gi|49821560[illegible]	26.94	43.52	193	114	7
unnamed	gi|48798779[illegible]	28.22	44.79	163	103	5
unnamed	gi|51637367[illegible]	24.8	41.06	246	156	8
unnamed	gi|44673307[illegible]	26.02	41.06	246	153	9
unnamed	gi|16982809[illegible]	30.49	53.66	82	51	3
unnamed	gi|39319931[illegible]	29.78	45.51	178	106	8
unnamed	gi|15894146	25.13	44.92	187	122	6
unnamed	gi|54746260[illegible]	27.2	46.4	125	85	3
unnamed	gi|48936531[illegible]	25.89	41.62	197	121	6
unnamed	gi|49071010[illegible]	30.23	44.19	129	77	5
unnamed	gi|58460471[illegible]	30.49	53.66	82	51	3
unnamed	gi|30022957	24.39	41.06	246	157	8
unnamed	gi|48800607[illegible]	26.89	42.44	238	145	10
unnamed	gi|51107246[illegible]	23.76	41.09	202	105	6
unnamed	gi|56981003[illegible]	23.65	46.31	203	135	8
unnamed	gi|50704996[illegible]	26.03	42.15	242	150	9
unnamed	gi|54730088[illegible]	25.85	48.3	147	88	6
unnamed	gi|40255496[illegible]	23.98	41.46	246	158	8
unnamed	gi|49054369[illegible]	25.89	43.15	197	121	7
unnamed	gi|51581991[illegible]	31.33	61.45	83	49	5
unnamed	gi|48937380[illegible]	25.51	42.35	196	123	7
unnamed	gi|44673312[illegible]	26.56	41.91	241	148	9
unnamed	gi|44673310[illegible]	25.73	41.91	241	150	8
unnamed	gi|38580953[illegible]	24.69	41.84	239	153	9
unnamed	gi|50698927[illegible]	24.8	40.65	246	156	8
unnamed	gi|29650535[illegible]	23.98	41.06	246	158	8
unnamed	gi|51771723[illegible]	27	52	100	64	2
unnamed	gi|38418271[illegible]	24.69	41.98	243	152	9
unnamed	gi|54469748[illegible]	28.46	50	130	81	5
unnamed	gi|49600026[illegible]	29.47	53.68	95	62	3
unnamed	gi|54947352[illegible]	34.62	53.85	78	47	2
unnamed	gi|48942571[illegible]	35.05	48.45	97	53	4
unnamed	gi|52369615[illegible]	27.75	44.02	209	120	11

unnamed	gi|22209837	25.73	41.49	241	150	8
unnamed	gi|51470091	22.73	41.74	242	153	8
unnamed	gi|21823492	24.39	41.06	246	157	8
unnamed	gi|48940177	25.38	41.62	197	122	6
unnamed	gi|49382818	25.85	47.62	147	88	6
unnamed	gi|48792509	25.63	42.02	238	148	8
unnamed	gi|49793568	24.3	52.34	107	63	3
unnamed	gi|25851726	22.39	47.26	201	136	6
unnamed	gi|57355925	24.06	40.11	187	124	7
unnamed	gi|48947376	34.26	50.93	108	59	5
unnamed	gi|49590803	22.22	43.39	189	125	6
unnamed	gi|31713294	24.86	44.51	173	115	6
unnamed	gi|49089183	22.61	44.22	199	140	6
unnamed	gi|37626154	25.79	44.44	252	148	12
unnamed	gi|44673307	24.49	41.22	245	158	9
unnamed	gi|48812708	24.87	41.62	197	123	6
unnamed	gi|51854602	21.72	41.39	244	145	8
unnamed	gi|49727758	33.33	58.62	87	56	2
unnamed	gi|44673309	24.49	42.45	245	158	10
unnamed	gi|44673306	24.7	40.89	247	155	9
unnamed	gi|49104163	23.76	44.06	202	134	8
unnamed	gi|54726960	27.27	49.59	121	65	5
unnamed	gi|44673306	24.39	41.06	246	157	8
unnamed	gi|12043776	24.03	48.84	129	85	4
unnamed	gi|49513887	26.8	50.52	97	66	3
unnamed	gi|49013818	26.06	44.15	188	104	8
unnamed	gi|51571759	26.77	41.41	198	118	8
unnamed	gi|57353508	30.48	44.76	105	63	4
unnamed	gi|44673307	25.2	41.06	246	155	9
unnamed	gi|54541645	29.67	44.02	209	118	11
unnamed	gi|40770742	29.2	43.8	137	85	4
unnamed	gi|54558717	30.33	42.62	122	72	4
unnamed	gi|44673306	24.39	41.06	246	157	8
unnamed	gi|50703920	24.39	41.06	246	157	8
unnamed	gi|44673306	25.2	41.87	246	155	9
unnamed	gi|54470588	27.81	40.64	187	117	6
unnamed	gi|48796898	24.87	41.62	197	123	6
unnamed	gi|49075532	28.17	46.48	213	124	11
unnamed	gi|57355110	29.52	47.62	105	64	4
unnamed	gi|51083738	44	54	50	27	1
unnamed	gi|26140456	33.33	58.62	87	56	2

unnamed	gi|57354852	24.65	41.86	215	142	7
unnamed	gi|44909187	24.39	40.65	246	157	8
unnamed	gi|57508304	26.6	46.28	188	114	8
unnamed	gi|15833941	22.74	39.71	277	168	12
unnamed	gi|48877219	26.67	43.08	195	119	8
unnamed	gi|30566601	26.62	42.45	139	86	3
unnamed	gi|48799032	24.87	41.62	197	123	6
unnamed	gi|38781767	26.74	42.78	187	119	7
unnamed	gi|48792787	24.87	41.62	197	123	6
unnamed	gi|48939667	24.87	41.12	197	123	6
unnamed	gi|25179822	25.27	44.51	182	107	8
unnamed	gi|48793243	24.87	41.62	197	123	6
unnamed	gi|22991708	25	41.67	180	113	5
unnamed	gi|57356743	22.49	44.98	249	153	10
unnamed	gi|48945336	27.27	40.11	187	118	6
unnamed	gi|54773621	30.7	49.12	114	74	4
unnamed	gi|51776235	24.42	54.65	86	61	2
unnamed	gi|54792449	29.55	51.14	88	50	3
unnamed	gi|23779475	27.37	40.53	190	120	6
unnamed	gi|48798240	32.04	46.6	103	60	3
unnamed	gi|37625993	23.81	48.57	105	65	3
unnamed	gi|48958654	24.87	42.86	189	126	5
unnamed	gi|48939919	32.04	46.6	103	60	3
unnamed	gi|49505847	29.27	47.56	82	55	1
unnamed	gi|35026819	22.59	42.26	239	149	8
unnamed	gi|49513388	24.74	49.48	97	68	3
unnamed	gi|51795193	26.51	59.04	83	56	3
unnamed	gi|29758495	25.55	48.18	137	96	4
unnamed	gi|49801828	22.59	42.26	239	149	9
unnamed	gi|56880307	25.34	42.08	221	132	9
unnamed	gi|17076083	25.87	42.79	201	130	8
unnamed	gi|51759728	26.77	39.9	198	118	8
unnamed	gi|49728168	22.18	43.93	239	157	9
unnamed	gi|54486842	28.79	60.61	66	46	1
unnamed	gi|49302329	26.44	39.42	208	126	8
unnamed	gi|51750031	33.09	49.64	139	75	6
unnamed	gi|48937895	24.87	41.12	197	123	6
unnamed	gi|51636049	25.13	44.39	187	122	6
unnamed	gi|22694867	27.27	40.11	187	118	6
unnamed	gi|48962085	25.26	52.63	95	66	3
unnamed	gi|48794614	23.86	41.62	197	125	6

unnamed	gi|489294075	26.77	39.9	198	118	8
unnamed	gi|507040674	26.77	39.9	198	118	8
unnamed	gi|487955320	32.04	45.63	103	60	3
unnamed	gi|559780403	25.74	42.57	202	129	8
unnamed	gi|573561278	22.59	43.51	239	156	9
unnamed	gi|310639652	28.3	44.34	106	64	3
unnamed	gi|489568782	25.81	45.16	186	120	8
unnamed	gi|218900067	25.2	40.65	246	155	9
unnamed	gi|261404312	22.59	43.51	239	156	9
unnamed	gi|516286616	27.71	54.22	83	57	2
unnamed	gi|261405053	25.14	41.9	179	114	7
unnamed	gi|153954305	27.5	55	80	52	2
unnamed	gi|153940784	26.2	42.78	187	120	7
unnamed	gi|558636418	22.18	41	239	155	8
unnamed	gi|435853834	24.21	42.63	190	121	6
unnamed	gi|544702972	25.37	42.79	201	131	8
unnamed	gi|488658777	25.26	51.58	95	66	3
unnamed	gi|305676543	22.59	42.68	239	149	9
unnamed	gi|387900561	20.58	42.39	243	164	8
unnamed	gi|386713350	20.21	44.68	188	122	6
unnamed	gi|507077847	32.43	56.76	74	48	2
unnamed	gi|499532765	26.51	53.01	83	49	2
unnamed	gi|573552848	26.51	54.22	83	58	2
unnamed	gi|488627944	25.26	51.58	95	66	3
unnamed	gi|547189466	28.18	47.27	110	66	3
unnamed	gi|187933797	37.14	52.86	70	39	3
unnamed	gi|547790058	25.26	50.53	95	66	3
unnamed	gi|489530178	24.66	45.29	223	131	11
unnamed	gi|549699755	20.76	42.8	236	158	8
unnamed	gi|488635819	25.26	52.63	95	66	3
unnamed	gi|488638300	25.26	51.58	95	66	3
unnamed	gi|515922544	37.14	52.86	70	39	3
unnamed	gi|494670209	24.04	42.08	183	121	6
unnamed	gi|488776799	36.54	57.69	52	31	1
unnamed	gi|271962232	20.38	44.59	157	106	6
unnamed	gi|545133357	20.76	42.8	236	158	8
unnamed	gi|384267427	20.76	42.8	236	158	8
unnamed	gi|374321530	22.99	42.25	187	127	6
unnamed	gi|493616675	23.85	48.46	130	92	4
unnamed	gi|547870754	21.46	41.95	205	138	5
unnamed	gi|148379374	25.37	42.79	201	131	8

unnamed	gi|515997289	42.22	55.56	45	25	1
unnamed	gi|489328934	22.18	41.84	239	150	9
unnamed	gi|518515588	42.22	55.56	45	25	1
unnamed	gi|595631638	42.22	55.56	45	25	1
unnamed	gi|507078202	24.14	47.13	87	63	1
unnamed	gi|515988008	20.76	42.8	236	158	8
unnamed	gi|515504206	20.94	41.88	234	160	8
unnamed	gi|386738788	31.07	46.6	103	61	3
unnamed	gi|589400827	26.74	55.81	86	58	3
unnamed	gi|518089680	23.46	39.92	243	152	11
unnamed	gi|550549515	23.93	44.17	163	113	4
unnamed	gi|549309973	24.66	44.84	223	131	11
unnamed	gi|260685101	24.66	44.84	223	131	11
unnamed	gi|497280753	25.14	41.34	179	114	7
unnamed	gi|516362687	27.5	55	80	52	2
unnamed	gi|547235249	28.46	43.9	123	75	3
unnamed	gi|545027476	24.66	44.84	223	131	11
unnamed	gi|488637680	30.67	57.33	75	44	4
unnamed	gi|489582224	25.26	40.53	190	124	6
unnamed	gi|544988426	23.48	42.61	230	137	9
unnamed	gi|429507190	20.34	42.8	236	159	8
unnamed	gi|585386197	27.5	45.62	160	94	6
unnamed	gi|597504678	20.34	42.8	236	159	8
unnamed	gi|585392324	27.27	45.45	165	98	6
unnamed	gi|517425547	32.43	58.11	74	38	4
unnamed	gi|517763098	26.42	44.56	193	120	10
unnamed	gi|488669410	31.58	46.05	76	34	2
unnamed	gi|497713292	27.63	50	76	49	1
unnamed	gi|407475206	50	73.33	30	15	0
unnamed	gi|547472300	25.79	44.03	159	102	6
unnamed	gi|375364327	20.68	43.04	237	157	9
unnamed	gi|488671561	26.32	51.58	95	65	3
unnamed	gi|565990129	30.77	44.87	78	41	2
unnamed	gi|489525121	34.78	50	92	54	3
unnamed	gi|488662688	30.67	56	75	44	4
unnamed	gi|545050343	24.22	44.84	223	132	11
unnamed	gi|546802313	25.58	52.33	86	59	3
unnamed	gi|513041249	22.3	46.04	139	98	3
unnamed	gi|495954157	36.17	59.57	47	29	1
unnamed	gi|493325504	30.99	47.18	142	70	7
unnamed	gi|494120031	23.58	39.62	212	109	8

<table>
<tr><td>unnamed</td><td>gi|544958162</td><td>24.22</td><td>44.84</td><td>223</td><td>132</td><td>11</td></tr>
<tr><td>unnamed</td><td>gi|545043723</td><td>24.22</td><td>44.84</td><td>223</td><td>132</td><td>11</td></tr>
<tr><td>unnamed</td><td>gi|518906704</td><td>26.64</td><td>42.52</td><td>214</td><td>120</td><td>9</td></tr>
<tr><td>unnamed</td><td>gi|515937954</td><td>28.57</td><td>53.33</td><td>105</td><td>65</td><td>4</td></tr>
</table>

q. start, q. end, s. start, s. end, evalue, bit score

1	285	1	285	0	576
1	285	1	285	0	575
1	285	1	285	0	569
1	285	1	285	0	567
1	285	1	285	0	566
1	285	1	285	0	565
1	285	1	285	0	563
1	285	1	285	0	563
1	285	1	285	0	561
1	285	1	285	0	556
1	285	1	285	0	555
1	285	1	285	0	552
1	285	1	285	0	550
1	285	1	285	0	546
1	285	1	285	0	542
1	285	1	286	1.00E-170	485
1	285	1	286	3.00E-170	484
1	285	1	286	6.00E-170	484
1	285	1	286	8.00E-170	483
1	285	1	286	1.00E-169	483
1	285	1	286	1.00E-169	483
1	285	1	286	1.00E-169	483
1	285	1	286	2.00E-169	483
1	285	1	286	2.00E-169	482
1	285	1	286	4.00E-169	481
1	285	1	286	6.00E-169	481
1	285	1	286	3.00E-168	479
1	285	1	285	3.00E-160	459
1	285	1	285	8.00E-160	458
1	285	1	286	2.00E-158	454
1	285	1	286	2.00E-149	431
1	285	1	286	6.00E-149	430
1	285	1	286	3.00E-147	426

1	285	1	286	4.00E-147	426
1	285	1	287	5.00E-107	324
1	285	1	287	9.00E-107	323
1	285	1	287	9.00E-106	320
1	285	1	287	4.00E-105	319
1	285	1	285	1.00E-102	313
1	285	1	285	2.00E-102	312
1	285	1	285	3.00E-102	311
1	285	1	285	1.00E-101	310
1	285	1	285	1.00E-101	310
1	285	1	285	2.00E-101	310
1	285	1	285	3.00E-101	309
1	285	1	284	2.00E-99	304
1	285	1	294	4.00E-90	281
1	285	1	293	1.00E-84	266
1	285	1	295	2.00E-84	266
1	285	1	296	7.00E-84	265
1	285	3	294	1.00E-83	264
1	285	1	297	2.00E-83	264
1	285	1	293	4.00E-83	263
2	285	3	292	6.00E-83	263
1	285	1	295	1.00E-82	262
1	285	1	293	2.00E-82	261
1	285	1	290	5.00E-82	260
2	285	3	300	1.00E-81	259
2	285	3	300	2.00E-81	259
1	285	1	301	9.00E-81	257
1	285	1	301	1.00E-80	257
1	162	1	162	2.00E-80	251
1	285	1	293	3.00E-80	256
5	285	4	289	4.00E-80	255
1	285	1	298	1.00E-79	254
1	285	1	287	1.00E-79	253
1	285	1	295	5.00E-79	252
2	285	3	295	1.00E-78	251
1	285	1	289	5.00E-78	249
1	285	1	293	1.00E-77	249
2	285	3	300	2.00E-77	249
2	285	3	300	3.00E-77	248
1	285	1	296	2.00E-76	246
2	285	3	301	8.00E-76	244

2	285	3	300	1.00E-75	244
3	285	2	290	1.00E-75	243
3	285	2	288	2.00E-75	243
11	285	11	296	8.00E-75	242
1	285	1	296	1.00E-74	241
5	285	6	302	7.00E-74	239
5	285	11	307	8.00E-74	239
1	285	1	295	1.00E-73	238
25	285	1	268	5.00E-73	236
1	285	1	295	8.00E-73	236
1	285	6	301	1.00E-72	236
1	285	1	290	5.00E-72	234
6	285	16	307	1.00E-71	234
1	285	2	286	2.00E-71	233
1	285	2	286	3.00E-71	232
1	285	1	295	3.00E-71	233
2	285	8	302	8.00E-71	231
1	285	1	290	2.00E-70	230
3	285	5	291	5.00E-70	229
2	285	3	301	1.00E-69	229
2	285	3	301	1.00E-69	228
1	285	1	300	2.00E-69	228
2	285	3	301	2.00E-69	228
1	285	1	284	3.00E-69	227
5	285	1	289	3.00E-69	227
2	285	3	301	2.00E-67	223
4	285	3	296	4.00E-67	222
1	285	1	297	5.00E-67	222
10	278	12	285	5.00E-66	219
1	285	1	283	1.00E-65	218
4	277	6	289	1.00E-64	215
4	278	6	284	7.00E-64	213
10	278	12	285	7.00E-64	213
70	285	1	222	2.00E-63	209
3	284	2	288	4.00E-63	211
4	277	6	289	3.00E-62	209
4	277	6	289	3.00E-62	209
4	277	6	289	3.00E-62	209
5	285	1	282	3.00E-62	209
4	277	6	289	3.00E-62	209
4	277	6	289	4.00E-62	209

4	277	6	289	6.00E-62	208
4	277	6	289	9.00E-62	208
4	277	6	289	9.00E-62	208
4	277	6	289	4.00E-61	206
4	277	6	289	6.00E-61	206
4	277	6	289	1.00E-60	205
5	285	2	283	4.00E-60	203
5	285	1	282	6.00E-60	203
1	285	1	299	1.00E-57	197
3	278	5	295	6.00E-57	196
3	269	2	270	1.00E-55	191
3	269	2	270	2.00E-55	191
3	269	2	270	3.00E-55	191
3	278	5	295	5.00E-55	191
50	285	3	237	5.00E-55	189
2	278	4	284	3.00E-54	188
5	285	9	294	5.00E-50	177
5	285	9	294	7.00E-50	177
5	285	9	294	8.00E-50	177
5	285	9	294	8.00E-50	177
5	285	9	294	1.00E-49	176
5	285	9	294	1.00E-49	176
5	285	9	294	1.00E-49	176
5	285	9	294	1.00E-49	176
5	285	9	294	1.00E-49	176
5	285	9	294	2.00E-49	176
5	285	9	294	2.00E-49	176
5	285	9	294	2.00E-49	176
5	285	9	294	2.00E-49	176
5	285	9	294	2.00E-49	176
5	285	9	294	2.00E-49	176
5	285	9	294	2.00E-49	176
5	285	9	294	2.00E-49	176
5	285	9	294	2.00E-49	176
4	276	8	289	4.00E-49	175
5	285	9	294	4.00E-49	175
5	285	9	294	5.00E-49	175
43	285	10	250	5.00E-49	173
5	285	9	294	6.00E-49	174
5	285	9	294	7.00E-49	174
5	277	9	290	9.00E-49	174

5	285	9	294	9.00E-49	174
5	285	9	294	9.00E-49	174
17	285	1	274	3.00E-48	172
5	285	9	294	4.00E-48	172
5	285	9	294	5.00E-48	172
46	277	15	249	2.00E-47	169
5	285	9	294	2.00E-47	170
2	278	3	295	7.00E-47	169
5	285	8	296	3.00E-46	167
46	277	15	250	5.00E-46	166
46	277	15	250	7.00E-46	165
46	277	15	250	2.00E-45	164
3	277	12	295	2.00E-45	165
46	277	15	250	2.00E-45	164
46	277	15	250	3.00E-45	164
46	277	15	250	2.00E-44	162
46	277	15	250	3.00E-44	161
1	270	28	333	5.00E-44	163
5	284	8	297	5.00E-44	162
1	270	28	333	6.00E-44	163
4	278	5	288	6.00E-44	161
26	284	36	293	7.00E-44	161
2	285	3	314	7.00E-44	162
5	277	1	282	8.00E-44	161
46	277	15	250	9.00E-44	160
5	285	9	294	1.00E-43	161
5	284	8	297	1.00E-43	161
5	284	8	297	1.00E-43	160
46	277	15	250	2.00E-43	159
5	277	9	288	2.00E-43	160
5	284	8	297	2.00E-43	160
46	277	15	250	2.00E-43	159
5	284	8	297	2.00E-43	160
46	277	15	250	2.00E-43	159
5	284	8	297	2.00E-43	160
46	277	15	250	3.00E-43	159
46	277	15	250	3.00E-43	159
5	284	8	297	3.00E-43	160
5	284	8	297	5.00E-43	159
46	277	15	250	5.00E-43	158
46	277	15	250	6.00E-43	157

46	277	15	250	1.00E-42	157
3	277	10	295	1.00E-42	158
5	284	8	297	1.00E-42	158
46	277	15	250	1.00E-42	157
25	284	31	285	1.00E-42	157
94	277	16	203	2.00E-42	155
3	284	6	295	2.00E-42	157
4	278	3	313	3.00E-42	157
46	277	15	250	5.00E-42	155
3	284	6	295	6.00E-42	156
34	284	45	297	9.00E-42	156
25	284	31	285	9.00E-42	155
34	284	45	297	1.00E-41	155
25	284	31	285	1.00E-41	155
34	284	45	297	2.00E-41	155
25	284	24	278	2.00E-41	154
23	284	33	297	2.00E-41	155
34	284	45	297	2.00E-41	155
34	284	45	297	2.00E-41	155
34	284	45	297	2.00E-41	155
34	284	45	297	3.00E-41	154
46	277	15	250	3.00E-41	153
34	284	45	297	3.00E-41	154
34	284	45	297	3.00E-41	154
46	277	15	250	7.00E-41	152
34	284	45	297	7.00E-41	154
34	284	45	297	9.00E-41	153
34	284	45	297	1.00E-40	153
5	285	6	300	2.00E-40	152
5	270	12	280	2.00E-40	152
2	277	6	290	3.00E-40	152
2	277	6	290	5.00E-40	151
2	277	6	290	8.00E-40	150
5	273	12	283	8.00E-40	150
1	278	1	305	1.00E-39	150
3	277	4	282	1.00E-39	150
2	277	6	290	2.00E-39	150
5	277	1	282	2.00E-39	149
2	273	6	286	2.00E-39	149
2	273	6	286	2.00E-39	149
2	273	6	286	2.00E-39	149

2	277	6	290	3.00E-39	149
5	285	3	272	4.00E-39	148
96	277	3	188	5.00E-39	145
3	284	6	294	6.00E-39	148
206	285	1	80	8.00E-39	141
3	284	6	294	9.00E-39	148
46	285	55	296	1.00E-38	147
3	284	6	287	3.00E-38	146
3	278	4	295	9.00E-38	145
1	278	1	317	2.00E-37	145
4	273	8	286	6.00E-37	143
31	278	43	282	4.00E-36	140
2	269	6	280	7.00E-36	140
3	284	6	287	8.00E-36	140
1	269	1	278	9.00E-36	140
67	278	1	224	1.00E-35	138
5	285	3	272	4.00E-35	137
127	277	2	153	4.00E-35	134
1	278	1	287	5.00E-35	138
7	278	8	301	5.00E-35	138
1	277	21	312	6.00E-35	138
7	278	8	301	9.00E-35	137
7	278	8	301	9.00E-35	137
1	285	1	310	2.00E-34	136
58	270	71	276	3.00E-34	135
4	277	16	294	1.00E-33	134
4	269	3	280	2.00E-33	133
50	277	54	297	6.00E-33	132
91	276	5	191	1.00E-32	129
39	278	7	253	3.00E-32	129
3	281	2	290	4.00E-32	130
2	278	4	284	1.00E-31	129
3	278	18	334	3.00E-31	129
43	277	25	273	7.00E-31	126
35	279	33	272	2.00E-30	125
2	278	3	291	5.00E-30	124
46	273	50	281	1.00E-29	123
29	273	26	281	2.00E-29	123
52	275	75	313	2.00E-29	123
1	281	4	290	3.00E-29	122
3	278	5	326	5.00E-29	122

1	270	1	283	8.00E-29	121
56	278	94	317	3.00E-28	120
45	277	45	286	7.00E-28	119
45	277	45	286	7.00E-28	118
45	277	45	286	7.00E-28	118
45	277	45	286	8.00E-28	118
9	269	13	306	9.00E-28	119
56	277	81	320	1.00E-27	119
29	280	40	282	1.00E-27	118
4	269	6	284	2.00E-27	117
45	277	45	286	2.00E-27	117
2	278	20	302	2.00E-27	117
3	281	2	290	3.00E-27	117
45	277	45	286	3.00E-27	116
4	269	3	295	3.00E-27	117
3	281	2	290	4.00E-27	116
3	281	2	290	4.00E-27	116
1	270	1	284	5.00E-27	116
1	269	6	317	5.00E-27	117
12	269	11	306	6.00E-27	116
58	278	67	297	6.00E-27	116
2	277	5	312	7.00E-27	116
4	269	3	293	8.00E-27	115
3	281	2	290	1.00E-26	115
12	269	11	316	1.00E-26	115
1	270	1	284	1.00E-26	115
9	269	13	306	2.00E-26	115
56	277	60	293	3.00E-26	114
3	281	2	290	4.00E-26	114
4	269	3	281	5.00E-26	113
45	277	45	286	5.00E-26	113
45	277	45	286	7.00E-26	113
191	278	1	90	8.00E-26	107
34	277	2	263	3.00E-25	111
126	274	79	224	3.00E-25	110
28	275	35	288	4.00E-25	110
28	275	35	288	1.00E-24	109
135	277	122	263	1.00E-24	109
141	274	90	224	1.00E-24	108
4	269	3	279	3.00E-24	108
4	269	3	279	3.00E-24	108

9	269	6	291	4.00E-24	108
9	269	5	290	4.00E-24	108
52	270	3	232	1.00E-23	105
4	269	3	296	1.00E-23	107
4	269	3	289	2.00E-23	106
51	269	23	258	2.00E-23	105
51	269	13	248	3.00E-23	105
62	271	41	255	3.00E-23	105
7	273	4	281	5.00E-23	105
4	269	3	293	7.00E-23	104
98	279	51	238	8.00E-23	103
72	277	1	218	2.00E-22	102
9	278	5	287	2.00E-22	103
67	269	1	222	6.00E-22	100
3	284	2	319	1.00E-21	102
3	284	2	319	1.00E-21	102
5	212	9	217	2.00E-21	99.4
5	269	4	279	7.00E-21	99
4	269	3	291	6.00E-20	96.3
4	269	3	291	1.00E-19	95.5
22	211	21	220	2.00E-19	94
4	269	3	291	5.00E-19	93.6
33	269	37	295	1.00E-18	92.4
44	273	49	318	1.00E-18	92.4
46	269	53	289	3.00E-18	91.3
15	270	26	288	3.00E-18	91.3
46	273	52	315	4.00E-18	91.3
44	273	49	318	5.00E-18	90.9
85	269	33	227	8.00E-18	89.4
1	269	6	306	2.00E-17	89.4
1	269	6	306	3.00E-17	88.6
34	274	43	293	5.00E-17	87.8
4	128	6	133	7.00E-17	84.7
24	270	17	293	4.00E-16	85.1
5	269	4	279	1.00E-15	83.6
3	269	2	308	2.00E-15	85.1
51	269	114	353	2.00E-15	84
3	269	2	308	2.00E-15	84.3
51	269	114	353	2.00E-15	83.6
49	271	14	231	5.00E-14	78.2
58	269	82	305	1.00E-13	78.2

96	277	32	228	2.00E-13	76.6
93	278	18	192	2.00E-13	76.3
58	269	82	305	4.00E-13	77
95	277	118	318	5.00E-13	76.6
5	269	12	284	9.00E-13	75.5
58	269	82	305	2.00E-12	76.3
67	269	1	227	2.00E-12	73.9
5	269	4	278	5.00E-12	73.2
43	270	8	231	5.00E-12	72.8
87	269	14	186	5.00E-12	72
43	270	8	231	6.00E-12	72.4
43	278	16	252	9.00E-12	73.9
1	179	1	182	3.00E-11	71.6
53	275	16	231	5.00E-11	69.7
35	266	3	243	1.00E-10	68.9
99	269	25	184	4.00E-10	67
99	269	25	184	4.00E-10	66.6
96	278	22	193	7.00E-10	65.9
99	269	25	184	8.00E-10	65.9
99	269	25	184	8.00E-10	65.9
43	270	8	231	8.00E-10	66.6
96	278	22	193	1.00E-09	65.1
85	278	635	819	2.00E-09	67.4
80	278	154	359	2.00E-09	66.6
99	269	27	186	3.00E-09	64.3
99	269	27	186	3.00E-09	64.3
99	269	25	184	4.00E-09	63.9
83	276	36	233	4.00E-09	64.3
36	270	8	244	5.00E-09	64.3
83	275	11	207	5.00E-09	63.9
83	275	45	241	6.00E-09	64.3
13	269	4	268	6.00E-09	64.7
43	278	8	253	6.00E-09	64.3
43	278	10	255	7.00E-09	64.3
155	269	106	216	7.00E-09	63.5
85	277	10	189	7.00E-09	63.2
155	269	106	216	8.00E-09	63.2
84	280	155	342	1.00E-08	64.7
36	270	3	239	1.00E-08	63.2
85	278	508	692	2.00E-08	64.3
85	278	15	191	2.00E-08	62

85	278	638	822	2.00E-08	64.3
96	269	22	184	2.00E-08	61.6
84	280	151	338	3.00E-08	63.2
83	274	54	251	3.00E-08	62.4
6	82	9	85	3.00E-08	63.2
43	278	6	240	3.00E-08	62
60	278	65	276	4.00E-08	62.4
60	278	63	274	4.00E-08	62.4
94	269	30	192	4.00E-08	61.2
60	278	45	256	4.00E-08	62
85	278	53	246	5.00E-08	62.4
103	270	215	356	5.00E-08	62.4
60	278	65	276	5.00E-08	62
43	278	6	240	5.00E-08	61.6
60	278	65	276	7.00E-08	61.6
60	278	65	276	7.00E-08	61.6
60	278	65	276	7.00E-08	61.6
60	278	65	276	8.00E-08	61.6
85	270	44	231	9.00E-08	60.8
60	278	65	276	1.00E-07	61.2
84	278	638	823	1.00E-07	62
84	278	638	823	1.00E-07	62
209	277	1	72	1.00E-07	57.4
106	285	274	448	2.00E-07	60.8
96	278	666	837	2.00E-07	61.2
9	98	4	93	2.00E-07	60.5
93	269	19	184	3.00E-07	58.9
95	270	68	245	3.00E-07	59.7
85	271	44	224	3.00E-07	58.9
106	285	274	448	3.00E-07	60.1
88	270	13	182	4.00E-07	58.5
85	274	44	235	4.00E-07	58.9
94	278	20	191	5.00E-07	58.2
1	194	1	194	5.00E-07	59.3
91	269	17	182	5.00E-07	57.8
140	270	107	231	6.00E-07	58.5
140	270	107	231	6.00E-07	58.5
135	270	118	231	6.00E-07	58.5
99	269	24	181	6.00E-07	57.8
95	270	54	231	9.00E-07	57.8
88	278	13	190	1.00E-06	57

125	269	94	235	1.00E-06	57.8
85	270	506	682	1.00E-06	58.9
84	270	63	250	1.00E-06	57.8
95	270	53	227	2.00E-06	57
80	274	26	224	2.00E-06	57
99	270	67	239	2.00E-06	57.8
96	278	652	823	2.00E-06	58.2
91	269	17	182	2.00E-06	56.2
96	278	61	247	3.00E-06	56.6
99	269	24	181	3.00E-06	55.8
9	98	4	93	3.00E-06	56.6
145	278	147	276	4.00E-06	56.6
84	270	163	342	4.00E-06	57
81	270	29	211	4.00E-06	55.8
87	270	631	805	4.00E-06	57.4
9	100	4	95	4.00E-06	56.6
80	269	3	184	4.00E-06	55.5
115	277	5	155	4.00E-06	54.7
96	270	627	790	5.00E-06	57
96	277	72	255	5.00E-06	56.2
94	281	18	191	5.00E-06	55.1
94	278	20	192	5.00E-06	55.1
84	278	657	842	5.00E-06	57
9	269	8	296	7.00E-06	55.8
94	259	19	171	8.00E-06	54.7
36	278	567	805	8.00E-06	56.2
84	278	42	244	8.00E-06	55.5
72	279	21	231	9.00E-06	55.1
135	269	73	183	1.00E-05	54.3
1	82	1	89	1.00E-05	55.5
93	269	19	184	1.00E-05	54.3
87	278	631	813	1.00E-05	55.8
80	270	26	220	1.00E-05	54.7
96	281	68	257	1.00E-05	55.1
96	181	17	106	1.00E-05	52.4
96	270	14	176	1.00E-05	53.9
91	270	52	231	1.00E-05	54.3
83	270	377	553	2.00E-05	55.5
135	269	73	183	2.00E-05	53.5
9	82	4	77	2.00E-05	54.7
9	82	4	77	2.00E-05	54.7

79	278	146	353	2.00E-05	55.1
94	270	19	182	2.00E-05	53.5
96	278	68	254	2.00E-05	54.3
83	270	174	354	2.00E-05	54.7
80	270	29	223	2.00E-05	53.9
9	82	4	77	2.00E-05	54.3
84	278	639	824	2.00E-05	55.1
84	277	51	245	2.00E-05	53.9
175	278	728	823	2.00E-05	55.1
9	98	4	94	3.00E-05	53.9
78	278	55	254	3.00E-05	53.9
144	278	34	163	3.00E-05	52.4
78	278	55	254	3.00E-05	53.9
46	284	12	245	3.00E-05	53.5
72	279	21	231	4.00E-05	53.1
9	94	4	89	4.00E-05	53.5
87	278	631	813	4.00E-05	54.3
142	282	5	138	4.00E-05	51.2
68	269	44	238	5.00E-05	53.5
87	270	643	817	5.00E-05	53.9
186	278	174	258	5.00E-05	53.1
87	270	631	805	5.00E-05	53.9
84	278	639	824	6.00E-05	53.9
87	270	616	790	6.00E-05	53.9
85	270	505	681	6.00E-05	53.5
80	270	26	220	6.00E-05	52.4
63	270	90	295	7.00E-05	52.8
72	279	21	231	8.00E-05	52.4
85	270	505	681	8.00E-05	53.1
83	270	177	368	8.00E-05	52.8
83	270	168	359	8.00E-05	52.8
95	269	21	182	8.00E-05	51.6
94	269	27	183	9.00E-05	51.6
80	270	29	223	9.00E-05	52
9	82	4	77	9.00E-05	52.4
83	270	168	359	9.00E-05	52.8
72	279	21	231	1.00E-04	52
106	285	276	451	1.00E-04	52.8
83	279	10	195	1.00E-04	51.6
96	269	22	184	1.00E-04	51.2
85	270	505	681	1.00E-04	52.8

87	270	631	805	1.00E-04	52.8
46	284	12	245	1.00E-04	51.6
46	278	11	238	1.00E-04	52.4
87	270	631	805	1.00E-04	52.8
72	279	21	231	1.00E-04	51.6
87	270	631	805	1.00E-04	52.4
96	270	663	826	2.00E-04	52.4
80	284	59	268	2.00E-04	52
72	279	21	231	2.00E-04	51.2
46	270	11	248	2.00E-04	51.6
96	270	381	542	2.00E-04	52
1	94	1	113	2.00E-04	51.6
76	274	51	232	2.00E-04	50.8
62	279	18	239	2.00E-04	50.8
1	94	1	113	2.00E-04	51.6
1	94	1	113	2.00E-04	51.2
51	278	9	235	2.00E-04	50.8
96	269	22	184	3.00E-04	50.4
72	279	21	231	3.00E-04	50.4
1	82	1	97	3.00E-04	51.2
145	284	117	245	3.00E-04	50.4
85	283	631	823	3.00E-04	51.6
85	270	173	363	3.00E-04	51.2
145	284	117	245	3.00E-04	50.4
85	270	639	815	3.00E-04	51.6
85	278	639	823	3.00E-04	51.6
96	270	663	826	4.00E-04	51.2
94	269	29	192	4.00E-04	49.7
89	278	89	264	4.00E-04	50.4
51	278	9	235	5.00E-04	50.1
52	278	10	235	5.00E-04	49.7
94	278	25	197	6.00E-04	49.3
77	270	44	237	6.00E-04	49.7
16	278	126	378	7.00E-04	50.1
47	278	5	235	7.00E-04	49.3
47	278	5	235	7.00E-04	49.3
39	278	11	248	7.00E-04	49.3
96	270	642	805	7.00E-04	50.4
72	279	21	231	9.00E-04	48.9
200	267	185	249	9.00E-04	49.3
94	278	18	188	9.00E-04	48.5

88	271	48	231	0.001	49.3
99	270	86	261	0.001	49.7
47	278	5	235	0.001	48.9
2	80	6	86	0.001	49.7
99	269	25	184	0.001	48.5
47	278	5	235	0.001	48.9
85	270	639	815	0.001	49.7
51	278	9	235	0.001	48.9
84	269	628	801	0.001	49.7
55	278	3	225	0.001	48.5
5	82	6	85	0.001	49.3
93	269	19	184	0.001	48.1
87	270	631	805	0.002	49.3
94	278	18	188	0.002	47.8
85	278	53	246	0.002	48.5
52	278	10	235	0.002	48.1
52	278	10	235	0.002	48.1
52	278	10	235	0.002	48.1
52	278	10	235	0.002	48.1
122	266	10	152	0.002	46.6
52	278	10	235	0.002	48.1
72	279	21	231	0.002	47.8
104	278	27	188	0.002	47.4
34	267	2	247	0.002	48.5
52	278	10	235	0.002	48.1
1	128	1	137	0.002	48.1
52	278	10	235	0.002	48.1
47	270	13	242	0.002	48.1
94	269	19	182	0.002	47.4
4	108	6	120	0.002	48.1
132	274	112	232	0.002	47.8
94	278	25	197	0.002	47.4
145	270	117	236	0.003	47.8
95	273	126	307	0.003	47.8
85	278	139	343	0.003	48.1
95	273	126	307	0.003	47.8
175	278	714	809	0.003	48.5
52	274	10	231	0.003	47.4
84	280	189	376	0.003	47.8
47	269	25	250	0.003	47.8
52	278	10	235	0.003	47.4

84	278	22	202	0.003	47
55	278	3	225	0.003	47.4
52	278	10	235	0.003	47.4
51	278	9	235	0.004	47.4
55	278	3	225	0.004	47
55	278	3	225	0.004	47
161	270	10	111	0.004	45.4
175	278	717	812	0.004	48.1
51	278	9	235	0.004	47
12	82	13	85	0.004	47.8
91	270	72	246	0.005	47
51	278	9	235	0.005	47
51	278	9	235	0.005	47
143	270	93	214	0.005	46.6
51	278	9	235	0.005	47
14	157	13	142	0.005	47.4
51	278	9	235	0.005	47
99	267	70	242	0.005	47
76	274	51	232	0.005	46.6
157	278	142	265	0.006	47
47	278	5	235	0.006	46.6
73	270	24	206	0.006	46.2
73	270	48	230	0.006	46.6
9	78	4	73	0.006	46.6
200	267	185	249	0.007	46.6
36	277	2	251	0.007	47
52	278	10	235	0.008	46.2
1	76	1	82	0.008	46.6
1	86	1	87	0.008	46.6
51	278	9	235	0.008	46.2
94	278	39	213	0.009	45.8
9	98	4	94	0.009	46.2
94	278	191	376	0.009	46.6
47	278	5	235	0.009	46.2
175	278	544	639	0.01	46.6
25	82	41	97	0.01	46.6
25	82	41	97	0.01	46.6
96	278	28	213	0.011	45.8
95	273	126	307	0.011	46.2
87	270	631	805	0.011	46.6
87	270	631	805	0.011	46.6

25	82	41	97	0.012	46.2
55	278	3	225	0.012	45.4
55	278	3	225	0.012	45.4
29	101	15	87	0.013	45.1
176	269	96	190	2.3	38.5
94	278	20	188	0.013	45.1
96	278	50	235	0.015	45.4
85	270	55	236	0.015	46.2
55	278	3	225	0.015	45.4
9	77	4	72	0.016	45.4
148	270	75	187	0.016	45.8
55	278	3	225	0.016	45.4
95	273	126	307	0.016	45.4
9	78	4	73	0.016	45.4
96	278	50	235	0.016	45.4
96	278	40	225	0.016	45.4
1	74	1	79	0.017	45.8
5	81	1	79	0.017	45.4
47	278	5	235	0.017	45.1
47	278	5	235	0.018	45.1
51	278	9	235	0.019	45.1
223	278	5	59	0.019	42.7
96	270	406	566	0.019	45.8
96	269	24	191	0.019	44.7
84	277	51	245	0.02	45.1
88	270	48	230	0.021	45.1
143	269	112	232	0.022	45.1
18	82	30	99	0.022	45.4
18	82	30	99	0.023	45.4
18	82	30	99	0.023	45.4
85	278	11	190	0.024	44.3
96	278	66	252	0.025	44.7
101	278	110	267	0.026	44.7
51	278	9	235	0.026	44.7
51	278	9	235	0.027	44.7
51	278	9	235	0.028	44.7
47	278	5	235	0.028	44.7
47	278	5	235	0.029	44.7
175	278	553	648	0.033	45.1
47	278	5	235	0.034	44.3
94	278	39	213	0.035	44.3

47	278	5	235	0.038	44.3
12	81	12	81	0.039	44.7
52	278	10	235	0.039	44.3
85	274	175	360	0.041	44.7
47	278	5	235	0.042	43.9
93	270	60	241	0.043	44.7
154	270	343	455	0.2	42.4
47	278	5	235	0.047	43.9
94	278	20	193	0.047	43.5
119	278	3	154	0.048	43.1
47	278	5	235	0.05	43.9
47	278	5	235	0.05	43.9
1	76	1	82	0.051	44.3
96	269	2	164	0.052	43.1
96	278	38	210	0.052	43.5
157	281	169	287	0.053	43.9
96	278	40	225	0.053	43.5
154	278	479	598	0.053	44.3
1	76	1	82	0.055	43.9
47	278	5	235	0.055	43.5
55	278	3	225	0.055	43.5
91	270	68	242	0.056	43.9
80	278	7	193	0.06	43.1
51	278	9	235	0.064	43.5
127	270	83	211	0.064	43.1
47	278	5	235	0.065	43.5
96	278	40	225	0.073	43.1
1	76	1	82	0.075	43.5
96	278	40	225	0.076	43.1
52	278	10	235	0.077	43.1
52	278	10	235	0.08	43.1
52	278	6	229	0.082	43.1
47	278	5	235	0.086	43.1
47	278	5	235	0.088	43.1
2	92	10	109	0.089	43.5
51	278	9	235	0.092	43.1
143	269	112	232	0.094	43.1
177	270	121	211	0.1	42.7
1	74	1	78	0.11	43.1
175	270	475	562	0.11	43.5
92	283	49	243	0.11	43.1

52	278	10	235	0.11	42.7
3	228	4814	5037	0.12	43.5
47	278	5	235	0.12	42.7
96	278	40	225	0.12	42.7
127	270	94	222	0.12	42.4
55	278	3	225	0.13	42.4
8	97	4	109	0.13	42.7
85	274	72	263	0.14	42.4
96	278	470	642	0.14	43.1
164	270	19	115	0.15	40.8
88	270	31	203	0.15	42.4
99	269	48	207	0.15	42.4
89	281	99	289	0.15	42.7
49	283	14	243	0.15	42.7
47	278	5	235	0.16	42.4
96	278	40	225	0.17	42
45	273	9	221	0.17	42
9	94	4	89	0.18	42.4
47	278	5	235	0.18	42
47	278	5	235	0.18	42
89	281	99	289	0.2	42.4
166	270	195	308	0.21	42
47	278	5	235	0.21	42
154	278	135	254	0.22	42
177	272	121	213	0.23	41.6
96	278	182	339	0.24	42
96	278	50	235	0.24	41.6
175	278	547	642	0.25	42.4
47	278	5	235	0.25	41.6
83	283	69	256	0.27	42
145	278	86	213	0.27	41.6
159	279	153	262	0.27	41.6
47	278	5	235	0.28	41.6
47	278	5	235	0.3	41.6
47	278	5	235	0.3	41.6
96	278	13	185	0.33	40.8
96	278	40	225	0.33	41.2
87	283	53	252	0.35	41.6
175	278	548	643	0.4	41.6
229	278	539	587	0.4	41.6
9	94	4	89	0.44	41.2

62	269	149	350	0.47	41.2
47	278	5	235	0.47	40.8
96	273	42	215	0.49	40.4
32	278	3	263	0.49	40.8
90	278	48	224	0.53	40.4
143	278	116	241	0.55	40.4
96	278	40	225	0.57	40.4
96	278	57	229	0.63	40.4
96	278	40	225	0.66	40.4
96	278	40	225	0.67	40.4
99	270	187	349	0.69	40.8
96	278	40	225	0.72	40
106	281	70	231	0.74	40.8
39	267	19	247	0.76	40.4
96	278	57	229	0.85	40
167	278	140	250	0.87	40
1	82	2	87	0.87	40.4
200	281	215	296	0.9	40
93	278	54	229	0.93	40
177	278	20	113	0.94	38.5
5	106	1	93	1.1	40
93	278	54	229	1.1	39.7
177	278	20	113	1.1	38.1
4	82	55	136	1.1	40
45	273	9	221	1.1	39.7
177	272	121	213	1.2	39.3
5	82	9	91	1.2	40
136	270	11	143	1.2	40
45	273	10	222	1.3	39.3
83	279	34	245	1.4	39.3
83	278	43	229	1.4	39.3
96	278	50	235	1.5	39.3
47	269	25	250	1.5	39.7
9	73	10	75	1.5	39.7
86	278	27	222	1.5	39.3
145	280	21	144	1.5	38.5
96	278	40	225	1.5	39.3
96	278	39	211	1.5	39.3
96	278	57	229	1.6	39.3
177	270	122	212	1.6	38.9
96	278	17	202	1.7	38.9

96	278	50	235	1.7	39.3
96	278	50	235	1.7	38.9
177	278	20	113	1.8	37.7
83	278	43	229	1.8	38.9
47	269	25	250	1.8	39.3
164	268	256	350	1.9	39.3
95	273	48	222	1.9	38.9
47	278	5	235	2	38.9
47	269	25	250	2	39.3
1	80	11	93	2.1	39.3
96	269	22	185	2.2	38.5
9	82	6	85	2.4	38.9
96	278	57	229	2.4	38.5
47	274	12	230	2.4	38.5
94	278	25	196	2.4	38.5
83	278	4	190	2.5	38.5
177	270	122	212	2.5	38.5
45	273	9	221	2.5	38.5
40	273	1	223	2.5	38.5
94	274	41	207	2.6	38.5
6	78	3	75	2.7	38.5
36	106	7	89	3	38.9
1	80	11	93	3	38.9
177	270	121	211	3	38.1
164	269	186	286	3	38.5
209	278	522	586	3	38.9
177	270	122	212	3.1	38.1
80	278	14	223	3.1	38.1
47	273	7	222	3.1	38.1
177	270	121	211	3.2	38.1
177	270	121	211	3.3	38.1
209	278	522	586	3.3	38.9
93	268	179	350	3.3	38.5
31	82	40	89	3.4	38.5
113	263	41	184	3.6	38.5
47	273	7	222	3.6	38.1
47	273	7	222	3.6	38.1
93	272	179	355	3.8	38.5
144	270	86	211	3.9	37.7
93	279	85	284	3.9	38.1
83	278	43	229	3.9	38.1

224	268	307	350	4.1	38.1
45	273	9	221	4.3	37.7
224	268	307	350	4.3	38.1
224	268	307	350	4.4	38.1
15	98	12	98	4.4	38.1
47	273	7	222	4.5	37.7
45	273	9	222	4.5	37.7
177	278	20	113	4.6	36.6
186	270	130	211	4.7	37.7
41	273	3	221	5	37.7
111	270	90	244	5	38.1
80	278	14	223	5.1	37.7
80	278	14	223	5.2	37.7
96	269	22	185	5.4	37.4
9	82	6	85	5.5	37.7
164	278	164	281	5.5	37.7
80	278	14	223	5.6	37.4
198	270	143	211	5.9	37.4
93	278	10	185	6	37
80	284	14	229	6.3	37.4
47	273	7	222	6.4	37.4
91	245	50	192	6.6	37.4
47	273	7	222	6.6	37.4
91	250	126	273	6.8	37.4
201	270	145	210	7.3	37
89	273	33	211	7.3	37
220	278	512	586	7.4	37.7
199	274	16	85	7.4	35.4
240	269	179	208	7.4	37.7
119	269	55	205	7.5	37
47	273	7	222	7.5	37
177	270	121	211	7.9	37
207	272	278	354	8.1	37.4
9	96	5	94	8.3	37
198	270	143	211	8.3	37
80	278	14	223	8.6	37
186	270	130	211	8.7	37
133	270	91	220	8.9	37.4
34	80	66	111	9	37.4
101	221	291	425	9.5	37.4
95	278	38	224	9.5	36.6

80	278	14	223	9.7	37
80	278	14	223	9.7	36.6
93	278	31	235	9.8	36.6
112	209	74	175	9.9	37.4
