# Supplementary material for: The Anti-sigma Factor RsiV Is a Bacterial Receptor for Lysozyme: Co-crystal Structure Determination and Demonstration That Binding of Lysozyme to RsiV Is Required for σV Activation
Source: PLoS Genet. 2016 Sep 7;12(9):e1006287. doi: 10.1371/journal.pgen.1006287 (PMC5014341; doi:10.1371/journal.pgen.1006287)
Supplement: S2 Table — (PDF) [file pgen.1006287.s013.pdf]

Table S2. Relative Expression in Response to Lysozyme exposure or IPTG Induction[1]

Strain/Condition:	Fold Induction:	RsiV molecules/cell	Lysozyme molecules/cell
PY79 Lysozyme 0	1	220	0
PY79 Lysozyme 1.25	99.41	16695	52640
PY79 IPTG	1	220	
JLH 402 IPTG	44.11	7419	

[1]qRT experiment, standardized to *rpoB*.
