# Supplementary material for: The Anti-sigma Factor RsiV Is a Bacterial Receptor for Lysozyme: Co-crystal Structure Determination and Demonstration That Binding of Lysozyme to RsiV Is Required for σV Activation
Source: PLoS Genet. 2016 Sep 7;12(9):e1006287. doi: 10.1371/journal.pgen.1006287 (PMC5014341; doi:10.1371/journal.pgen.1006287)
Supplement: S3 Table — (PDF) [file pgen.1006287.s014.pdf]

Table S3 Oligonucleotides

Primer	Sequence 5' to 3'	Description of use
CDEP3092	GCATCTTGGTACACACAAAGCCGTTATATTA CAATTGACAAAAAGAATG	Clone S169W
CDEP3093	TTGTGTGTACCAAGATGCTTGTGTTGTTTCA ATATTGCGCCG	Clone S169W
CDEP3108	GCCGCAGGCTATATGGGCGTTACAGAGTTT ACAATTCCAACCGGC	Clone P259A
CDEP3109	CCATATAGCCTGCGGCGACTTCATATTCGT CAAATGAGATCACAAGTTTATGGTC	Clone P259A
CDEP3110	GGCGCAATGGGCGTTACAGAGTTTACAATT CCAACCGGCGTCATTTCAAAC	Clone Y261A
CDEP3111	TAACGCCCATTGCGCCGGGGGCGACTTCAT ATTCGTCAAATGAGATCAC	Clone Y261A
CDEP3098	GTTGCTGCAGGAGCTATGGGCGTTACAGAG TTTACAATTCCAACCGGC	Clone P259A, Y261A
CDEP3099	CATAGCTCCTGCAGCAACTTCATATTCGTCA AATGAGATCACAAGTTTATGGTCTTCAGT	Clone P259A, Y261A
CDEP3116	AACAAAGCAGGGGCATCAGCATCTACAGTG ATTACGAGACGGTCAC	Clone K137A, H139A
CDEP3117	GAGTGCCCCTGCTTTGTTTTTGGATGTGGA CTGTATAAACTCTTTATACAGCTGCTG	Clone K137A, H139A
CDEP3163	TAGAATTCGAGCTCCCGGGACAAGCAATGA TATCACCG	Clone *oatA* onto pMAD
CDEP3136	TAGAATTCGAGCTCCCGGGACAAGCAATGA TATCACCG	Adds 1 kb for P259A and Y261A mutants
CDEP1107	TGTCAAAGATCCCTGTCATCGGCA	qRT PCR *rsiV*
CDEP1108	TTAGAAAGCCCGGACAAAGCAGGT	qRT PCR *rsiV*
CDEP1017	CAAACCGTGGCGCATGGTTAGAAT	qRT PCR *rpoB*
CDEP1018	CGGCAACTTACGTGTGCGATCAAT	qRT PCR *rpoB*
